# Molecular glue degrader function of SPOP inhibitors enhances STING-dependent immunotherapy efficacy in melanoma models

**DOI:** 10.1172/JCI191772

**Published:** 2025-10-28

**Authors:** Zhichuan Zhu, Xin Zhou, Max Xu, Jianfeng Chen, Kevin C. Robertson, Gatphan Atassi, Mark G. Woodcock, Allie C. Mills, Laura E. Herring, Gianpietro Dotti, Pengda Liu

**Affiliations:** 1 Lineberger Comprehensive Cancer Center,; 2 Department of Biochemistry and Biophysics,; 3 Department of Microbiology and Immunology,; 4 Department of Pharmacology,; 5 Division of Oncology, Department of Medicine, and; 6 UNC Metabolomics and Proteomics Core, Department of Pharmacology, The University of North Carolina at Chapel Hill, Chapel Hill, North Carolina, USA.

**Keywords:** Cell biology, Oncology, Cellular immune response, Skin cancer, Ubiquitin-proteosome system

## Abstract

The E3 ligase SPOP plays a context-dependent role in cancer by targeting specific cellular proteins for degradation, thereby influencing cell behavior. However, its role in tumor immunity remains largely unexplored. In this study, we revealed that SPOP targeted the innate immune sensor STING for degradation in a CK1γ phosphorylation-dependent manner to promote melanoma growth. Stabilization of STING by escaping SPOP-mediated degradation enhanced antitumor immunity by increasing IFN-β production and ISG expression. Notably, small-molecule SPOP inhibitors not only blocked STING recognition by SPOP, but also acted as molecular glues, redirecting SPOP to target neosubstrates such as CBX4 for degradation. This CBX4 degradation led to increased DNA damage, which in turn activated STING and amplified innate immune responses. In a xenografted melanoma B16 tumor model, single-cell RNA-seq analysis demonstrated that SPOP inhibition induced the infiltration of immune cells associated with anti–PD-1 responses. Consequently, SPOP inhibitors synergized with immune checkpoint blockade to suppress B16 tumor growth in syngeneic murine models and enhanced the efficacy of CAR.CD19-T cell therapy. Our findings highlight a molecular glue degrader property of SPOP inhibitors, with potential implications for other E3 ligase–targeting small molecules designed to disrupt protein–protein interactions.

## Abstract 2

SPOP inhibitors act as dual-function molecular glue degraders that stabilize and activate STING, boosting anti-melanoma immunity and enhancing responses to checkpoint and CAR-T therapies.

## Graphical abstract

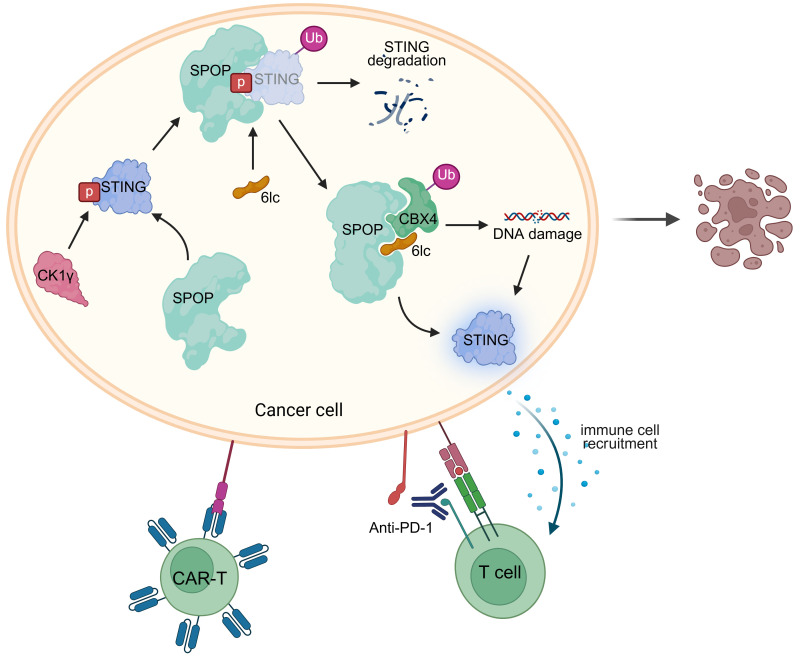

## Introduction

The ubiquitin–proteasome pathway is a major mechanism for regulated protein turnover. Among E1, E2, and E3 enzymes (1), E3 ubiquitin ligases confer substrate specificity by recruiting target proteins for ubiquitination and subsequent degradation. SPOP (speckle-type POZ protein), together with Rbx1 and Cullin 3, forms a Cullin-Ring E3 ligase complex, with SPOP serving as the substrate recognition subunit. SPOP targets diverse proteins for ubiquitination and degradation, including transcription modulators such as SRC3 (2), DEK (3), ATF2 (4), ERG (5, 6), EWS:FLI1 (7), and BRD4 (8, 9); enzymes such as TRIM24 (3) and PTEN (10); hormone receptors such as AR (11); apoptotic regulators such as Daxx (10); and cell cycle proteins such as Cdc20 (12) and cyclin E (13). Beyond degradation, SPOP mediates nondegradative ubiquitination, such as HIPK2 activation (14), K63-linked 53BP1 ubiquitination to impair DNA repair (15), and LMNB2 priming for WDR26-mediated degradation (16). Additionally, SPOP can function independently of its E3 ligase activity, for example, by binding and stabilizing ACE2 to facilitate SARS-CoV-2 infection (17).

The pathological role of SPOP in cancer is context dependent. In prostate cancer, SPOP mutations occur in approximately 10% of patients, where it acts as a tumor suppressor by degrading oncogenic transcription factors, including ERG (5, 6), DEK (3), and TRIM24 (3), and by mediating poly(ADP-ribose) polymerase inhibitor–induced tumor suppression via stimulator of interferon gene (STING) stabilization (18). In Ewing sarcoma, SPOP similarly suppresses tumors by targeting the EWS:FLI1 oncofusion protein (7). In contrast, SPOP exhibits oncogenic activity in kidney cancer by negatively regulating PTEN (10) and LATS1 (19). While SPOP’s regulation of intrinsic cellular programs is well documented, its role in immunity and the tumor microenvironment is less clear. SPOP has been reported to inhibit Toll-like receptor signaling (20) by modulating MyD88 ubiquitination (21) or degradation (22), yet it can also promote an immune-permissive environment by degrading IRF1 (23) or PD-L1 (24), enhancing immune checkpoint blockade (ICB) efficacy and chemosensitivity (25). It is unknown whether and how SPOP controls innate immunity and subsequent immune cell infiltrates in solid tumors.

Here, we demonstrate that SPOP acts as an oncogene in melanoma by targeting the innate immune sensor STING for ubiquitination and degradation. Loss of SPOP suppresses B16 tumor growth in a manner dependent on host immunity and tumor-intrinsic STING. Small-molecule SPOP inhibitors 6b and 6lc function as molecular glue degraders, recruiting CBX4 to mediate SPOP degradation, which induces DNA damage and activates STING. In B16 xenografts, scRNA-seq revealed that SPOP inhibitor–mediated STING stabilization enhances immune cell infiltration and potentiates anti–PD-1 responses, improving the efficacy of both ICB and CAR T cell therapies. Together, these findings identify a molecular glue mechanism for SPOP inhibitors and support their potential to sensitize tumors to immunotherapy.

## Results

### An intact immune microenvironment is crucial for suppressing B16 tumor growth following SPOP depletion.

While SPOP’s roles in prostate and kidney cancers are well established, its function in melanoma remains unclear. Similar to human renal cell carcinoma (RCC) 786-o and A498 cells, depletion of endogenous SPOP reduced colony formation in human melanoma A2058 and HMCB cells (Supplemental Figure 1, A and B; supplemental material available online with this article; https://doi.org/10.1172/JCI191772DS1). Likewise, SPOP knockdown in mouse RCC Renca and melanoma B16 cells impaired in vitro growth (Supplemental Figure 1, C–H). In RCC, SPOP exerts oncogenic activity by targeting the tumor suppressor PTEN (10), and analysis of The Cancer Genome Atlas revealed similar patterns of SPOP and PTEN alterations in KIRC and SKCM (Supplemental Figure 1I). These results suggest that SPOP may function as an oncogene in melanoma, analogous to its role in RCC.

**Figure 1:**
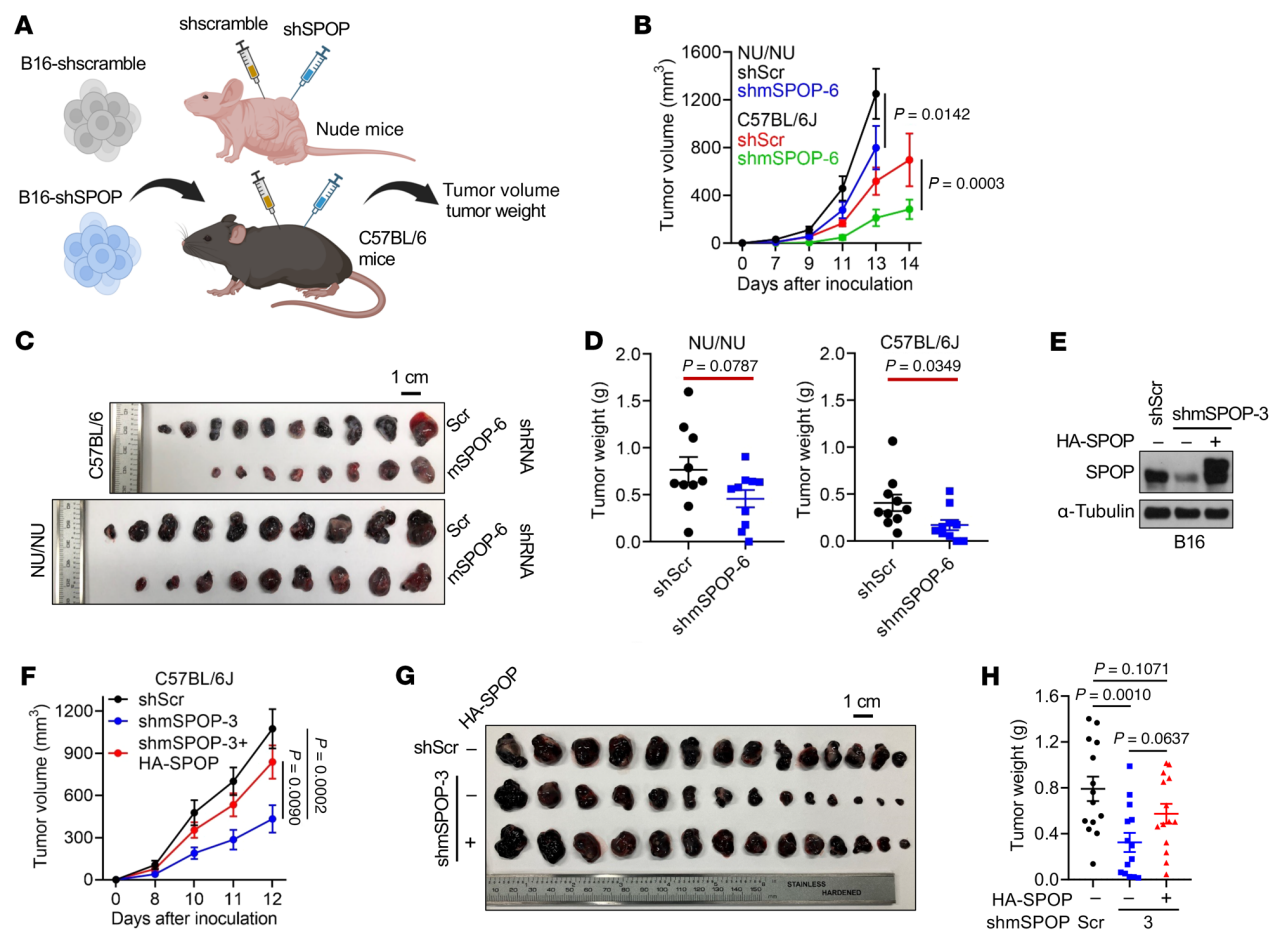
SPOP depletion suppresses B16 tumor growth depending on tumor immune environment. (**A**) Xenograft schema. Tumor volume and weight were measured in nude and C57BL/6J mice injected with B16 cells expressing shScr and shmSPOP-6. (**B**) Tumor volume measurements over time for xenograft of indicated B16 cell lines. Data are shown as mean ± SEM, *n* = 10. (**C** and **D**) Isolated tumors (**C**) from **B** and tumor weight (**D**). Scale bar: 1 cm. Data are shown as mean ± SEM, *n* = 10. (**E**) IB analyses of control, SPOP-depleted, and reconstituted B16 cells. (**F**) Tumor volume measurements over time for xenograft of B16 cells in **E**. Data are shown as mean ± SEM, *n* = 14. (**G** and **H**) Isolated tumors (**G**)from **F** and tumor weight (**H**). Scale bar: 1 cm. Data are shown as mean ± SEM, *n* = 14. Two-way ANOVA followed by Tukey’s multiple-comparison test (**B** and **F**), 2-tailed unpaired Student’s *t* test (**D**), or 1-way ANOVA followed by Fisher’s LSD multiple-comparison test (H). Representative experiments shown in figures were repeated at least 2 times independently with similar results.

**Figure 2:**
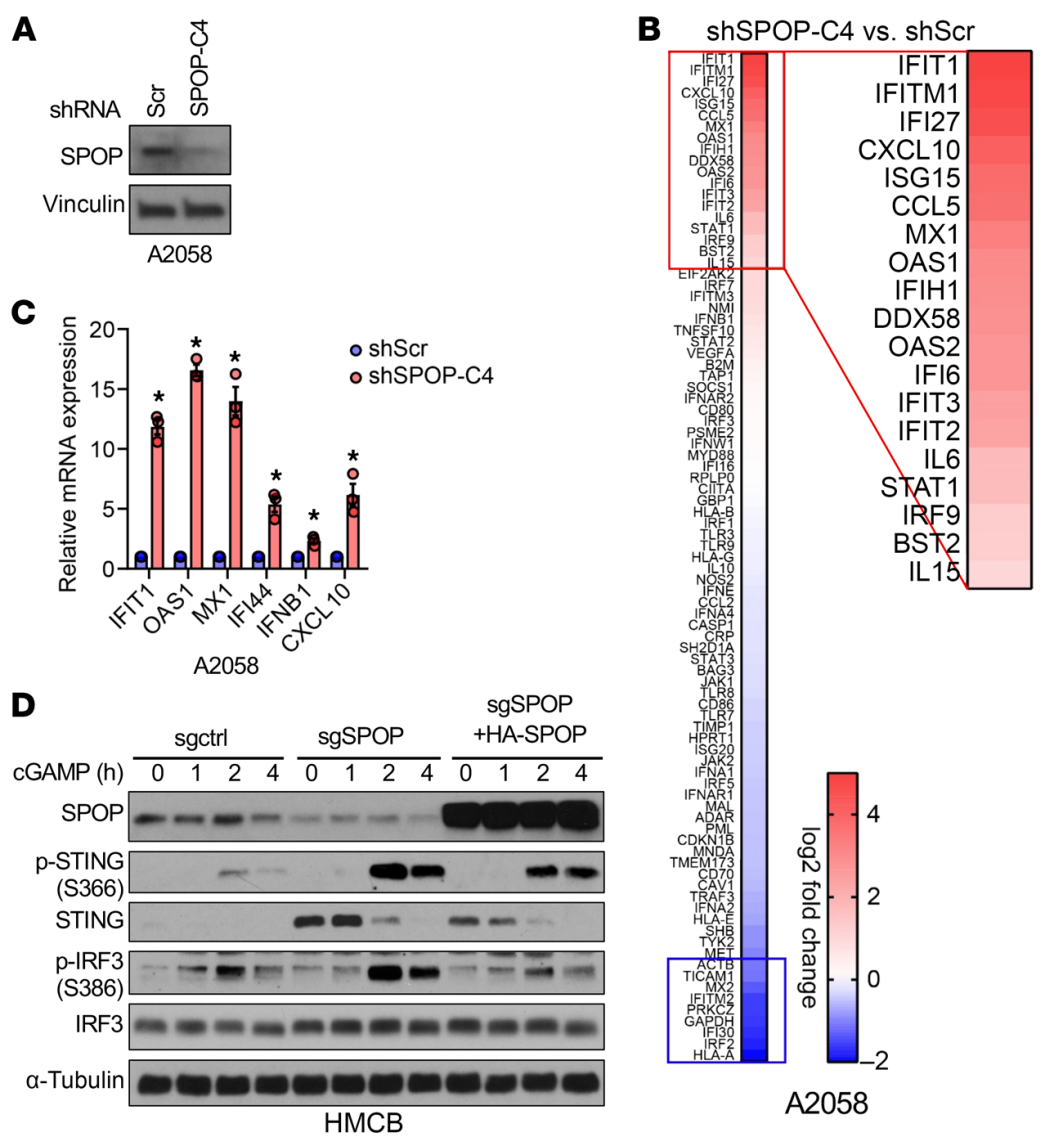
SPOP depletion potentiates type I interferon response. (**A**) IB analyses of control and SPOP-depleted A2058 cells. (**B**) RNA expression profiling heatmap of genes in human type I interferon response in A2058 cells from **A**. (**C**) RT-PCR analyses of mRNA changes in A2058 cells from **A**. Data are shown as mean ± SD, *n* = 3. Two-tailed unpaired Student’s *t* test. *P < 0.05. (**D**) IB analysis of indicated HMCB cells treated with 5 μg/mL 2′3′-cGAMP for indicated hours.

To assess SPOP’s role in tumor immunity, control or SPOP-depleted B16 cells were xenografted into immune-deficient nude mice or immune-competent C57BL/6 mice (Figure 1A). Consistent with in vitro data (Supplemental Figure 1E), SPOP depletion slightly reduced tumor growth in nude mice but markedly suppressed tumor growth in C57BL/6 mice (Figure 1, B–D), suggesting that host T cell immunity is required for SPOP depletion-mediated tumor suppression. Reexpression of SPOP largely rescued tumor growth in C57BL/6 mice, ruling out shRNA off-target effects (Figure 1, E–H). Cytokine profiling of SPOP-depleted human melanoma A2058 cells revealed increased expression of interferon-stimulated genes (ISGs), including IFIT1, CXCL10, and MX1, which was validated by RT-PCR (Figure 2, A–C). This finding was supported by xenografted SPOP-depleted B16 tumors, where SPOP loss led to increased CCL5 and CXCL10 expression (Supplemental Figure 1J). mRNA profiling of SPOP-depleted B16 cells also revealed upregulated ISGs (Supplemental Figure 1K). Given that type I interferons and ISGs mediate tumor innate immune activation and recruit immune infiltrates (26, 27), these findings indicate that SPOP depletion enhances tumor innate immunity to suppress melanoma growth. In this study, we focus on melanoma and RCC to determine whether SPOP regulates tumor immunity.

### SPOP depletion enhances cellular responses to DNA stimulation.

Since cytosolic DNA-sensing pathways drive ISG expression (28), we tested whether SPOP depletion alters responses to DNA stimulation. DNA sensing is a ubiquitous innate immune pathway in both immune and tumor cells, initiated when cyclic GMP-AMP synthase (cGAS) detects cytosolic DNA and produces 2′3′-cyclic GMP-AMP (2′3′-cGAMP), which activates STING to trigger TBK1/IRF3-dependent IFN-β and ISG expression (29–32). SPOP depletion markedly enhanced ISD90-induced STING activation, as shown by increased pSTING, an effect reversed by SPOP reexpression in HMCB melanoma cells (Figure 2D). Similar results were observed in RCC 786-o cells, where SPOP loss augmented STING signaling, increased IFN-β transcription, and upregulated multiple ISGs, including CCL5, CXCL10, OAS1, IFIT1, and IFI44 (Supplemental Figure 1, L–R). SPOP depletion also potentiated 2′3′-cGAMP– and diABZI-induced (33) STING activation and ISG production (Supplemental Figure 1, S–Z). Importantly, reintroducing SPOP largely reversed these effects (Supplemental Figure 1, Z1 and Z2). Collectively, these findings indicate that SPOP depletion sensitizes cells to cytosolic DNA stimulation by enhancing cGAS/STING signaling.

### SPOP earmarks STING for ubiquitination and degradation.

**Figure 3:**
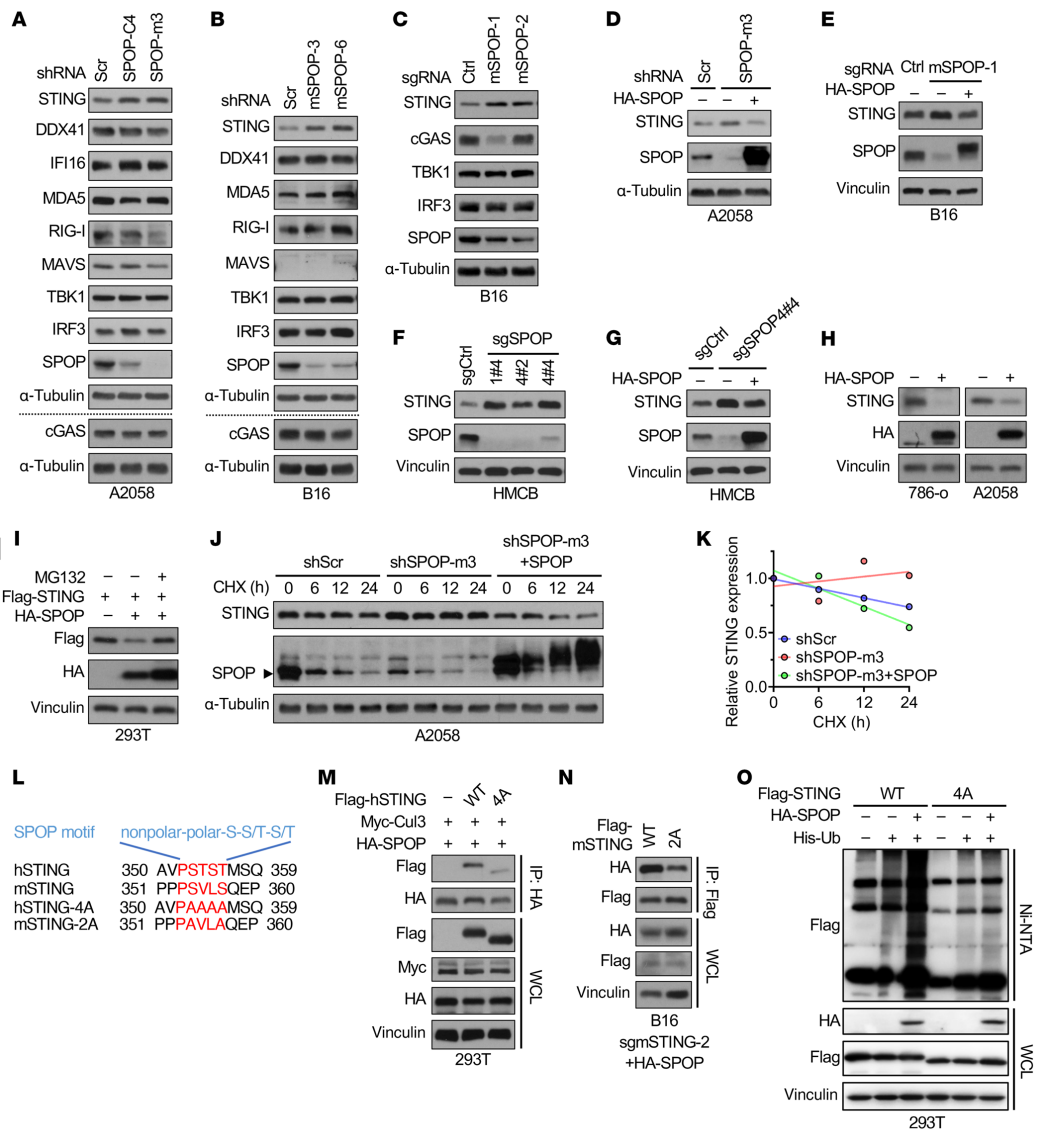
SPOP targets STING for ubiquitination and degradation. (**A**–**C**) IB analyses of indicated cells depleted of SPOP by shRNAs or sgRNAs. (**D**–**G**) IB analyses of indicated cells depleted of SPOP and rescued by stably expressing shRNA/sgRNA-resistant SPOP. (**H**) IB analyses of indicated cells transfected with HA-SPOP construct. (**I**) IB analyses of 293T cells treated with 10 μM MG132 overnight after transfecting with indicated constructs for 36 h. (**J**) IB analysis of control and SPOP-depleted A2058 cells treated with 100 μg/mL of cycloheximide (CHX) for indicated periods. (**K**) Quantification of relative STING grayscale values in **J**. (**L**) Schematic illustration of potential SPOP-binding motifs in human and mouse STING and corresponding mutations. (**M**) IB analyses of HA-IP (immunoprecipitants) and whole-cell lysates (WCL) derived from 293T cells transfected with indicated constructs. (**N**) IB analyses of Flag-IP and WCL derived from B16 cells stably expressing indicated molecules by lentivirus infection. (**O**) IB analyses of WCL and Ni-NTA pull-down products derived from 293T cells transfected with the indicated constructs. Cells in **L**–**N** were treated with 10 μM MG132 overnight before collection. Representative experiments shown in figures were repeated at least 2 times independently with similar results.

The cytosolic DNA-sensing pathway primarily involves cGAS, STING, TBK1, and IRF3 (32). To determine how SPOP depletion enhances DNA sensing, we silenced endogenous SPOP using multiple independent shRNAs/sgRNAs (shSPOP/sgSPOP). SPOP loss consistently increased STING protein levels, but not those of cGAS, TBK1, or IRF3, across human melanoma (A2058, HMCB, and MeWo), mouse melanoma (B16), human RCC (A498, 786-o, and UMRC6), mouse RCC (Renca), and HEK293 cells (Figure 3, A–C and F, and Supplemental Figure 2, A–H). Other DNA sensors, including DDX41 and IFI16, were minimally affected. SPOP depletion did not alter STING mRNA levels (Supplemental Figure 2I), suggesting posttranscriptional regulation. Reexpression of shSPOP/sgSPOP-resistant SPOP restored STING to baseline, confirming specificity (Figure 3, D, E, and G, and Supplemental Figure 2, J–L). Conversely, ectopic SPOP expression reduced endogenous and exogenous STING, which was reversible by proteasome inhibition (Figure 3, H and I). Cycloheximide chase assays further demonstrated that SPOP depletion stabilized STING, extending its half-life, which was reversed by SPOP reexpression (Figure 3, J and K, and Supplemental Figure 2, N–Q).

SPOP recognizes the degron motif Φ-Π-S-S/T-S/T (Φ, nonpolar; Π, polar) (6, 8). Sequence analysis identified the potential degron PSTST in human STING (Figure 3L). Mutation of these residues (S353A/T354A/S355A/T356A [4A-STING]) impaired SPOP binding (Figure 3M). Similarly, in mouse STING, mutation of PSVLS serines (S354A/S357A [2A-mSTING]) reduced interaction (Figure 3N). Moreover, SPOP efficiently ubiquitinated WT-STING but not 4A-STING (Figure 3O). Together, these results demonstrate that SPOP directly recognizes the PSTST degron to ubiquitinate and degrade STING.

### CK1γ generates a phospho-degron in STING for SPOP recognition.

**Figure 4:**
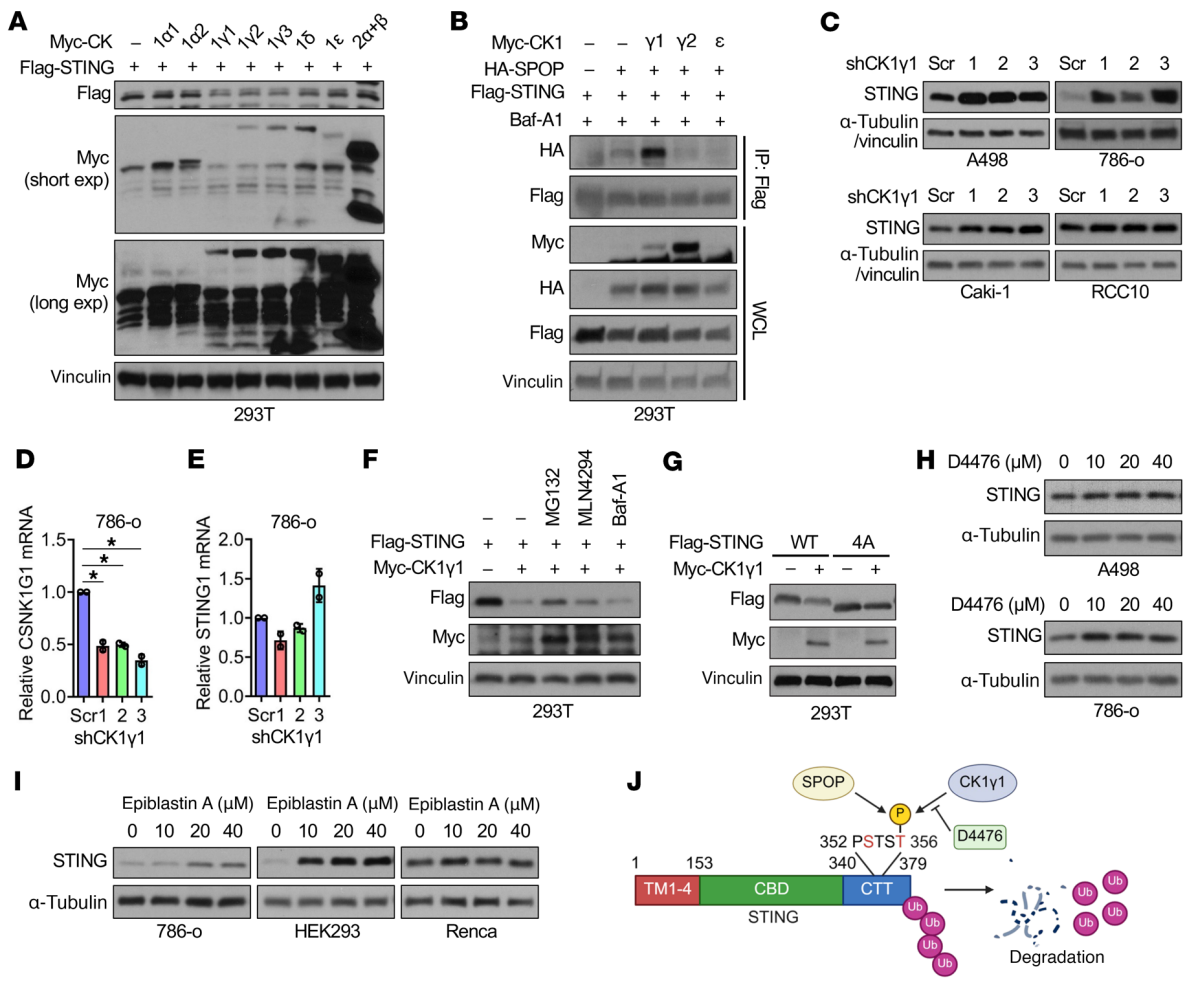
CK1γ1 phosphorylates STING for SPOP-mediated ubiquitination and degradation. (**A**) IB analyses of 293T cells cotransfected with constructs of Flag-STING and Myc-tagged casein kinases. (**B**) IB analyses of Flag-IP and WCL derived from 293T cells transfected with indicated constructs and overnight treated with 20 nM Baf-A1. (**C**) IB analyses of indicated cells depleted of CK1γ1. (**D** and **E**) RT-PCR analyses of mRNA changes in 786-o cells depleted of CK1γ1. Data are shown as mean ± SD, *n* = 2. One-way ANOVA followed by Dunnett’s multiple-comparison test. *P < 0.05, compared with shScr. (**F**) IB analyses of 293T cells treated overnight with 10 μM MG132, 1 μM MLN4294, and 20 nM Baf-A1 after transfecting with indicated constructs for 36 h. (**G**) IB analyses of 293T cells transfected with indicated constructs. (**H** and **I**) IB analyses of indicated cells treated with indicated doses of D4476 (**H**) and epiblastin A (**I**) for 24 h. (**J**) Schematic of STING degradation triggered by SPOP and CK1γ1. TM, transmembrane domain; CBD, cyclic dinucleotide–binding domain; CTT, C-terminal tail; p, phosphorylation. Representative experiments shown in figures were repeated at least 2 times independently with similar results.

Multiple S/T residues in the canonical SPOP Φ-Π-S-S/T-S/T degron can be phosphorylated to enhance SPOP binding (6–8, 17). Testing CK1 and CK2 isoforms revealed that CK1γ, specifically CK1γ1, promotes STING recognition by SPOP (Figure 4, A and B). CK1γ1 depletion in RCC cells (A498, 786-o, Caki-1, and RCC10) led to STING protein accumulation without affecting STING mRNA (Figure 4, C–E), indicating regulation at the protein level. CK1γ1-induced STING degradation was partially blocked by the proteasome inhibitor MG132 or the cullin neddylation inhibitor MLN4924, but not by the lysosomal inhibitor Baf-A1 (Figure 4F), and required the intact STING degron, as 4A-STING was resistant (Figure 4G). Pharmacological CK1 inhibition (D4476 or epiblastin A) similarly stabilized STING by reducing CK1γ1-mediated phosphorylation and SPOP binding (Figure 4, H and I, and Supplemental Figure 2R). These results indicate that CK1γ1 phosphorylates the STING PSTST motif to prime it for SPOP-mediated ubiquitination and degradation (Figure 4J).

### Evading SPOP-mediated degradation enhances STING activation in innate immunity.

**Figure 5:**
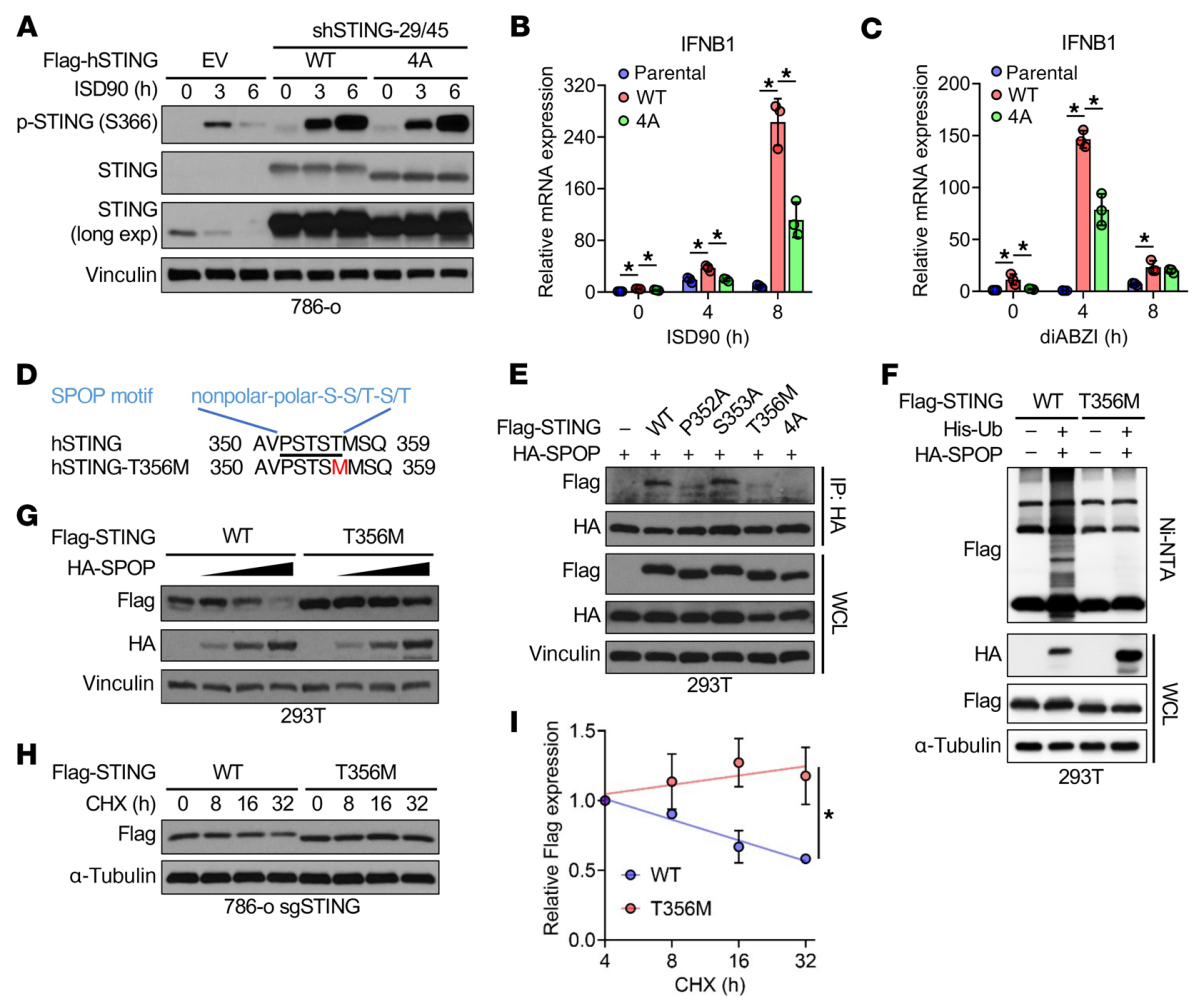
Evading SPOP-mediated degradation enhances STING activation in innate immunity. (**A**) IB analyses of indicated 786-o stable cell lines treated with 5 μg/mL of ISD90 for indicated periods. EV, empty vector. (**B** and **C**) RT-PCR analyses of indicated 786-o stable cell lines treated with 5 μg/mL of ISD90 (**B**) or 3 μM diABZI (**C**) for indicated periods. Data are shown as mean ± SD, *n* = 3. (**D**) Schematic illustration of patient STING-T356M mutation in the SPOP-binding motif. (**E**) IB analyses of HA-IP and WCL derived from 293T cells transfected with indicated constructs. (**F**) IB analyses of WCL and Ni-NTA pull-down products derived from 293T cells transfected with the indicated constructs. Cells in **E** and **F** were treated with 10 μM MG132 overnight before collection. (**G**) IB analyses of 293T cells transfected with fixed dose of STING constructs and increased dose of SPOP construct. (**H**) IB analysis of Flag-STING-WT– and -T356M–reconstituting 786-o cells treated with 100 μg/mL of CHX for indicated periods. (**I**) Quantification of relative Flag grayscale values in **H**. Data are shown as mean ± SEM, *n* = 2. One-way ANOVA followed by Tukey’s multiple-comparison test (**B** and **C**) or 2-way ANOVA (**I**). *P < 0.05. Representative experiments shown in figures were repeated at least 2 times independently with similar results.

We next asked whether STING stabilization by evading SPOP-mediated degradation enhances innate immune activation. Reexpression of WT- or 4A-STING in STING-depleted 786-o cells showed comparable ISD90-induced STING activation (Figure 5A), but RT-PCR revealed that 4A-STING induced significantly lower IFN-β and ISG (CCL5 and CXCL10) expression than WT-STING after ISD90 or diABZI stimulation (Figure 5, B and C, and Supplemental Figure 3, A–D). This suggested that loss of phosphorylation within the degron impairs STING activation. Consistent with prior reports that TAK1 phosphorylates STING at S355 to facilitate ER-to-ERGIC (ER-Golgi intermediate compartment) trafficking (34), S355F-STING failed to rescue STING activation in depleted cells (Supplemental Figure 3E).

**Figure 6:**
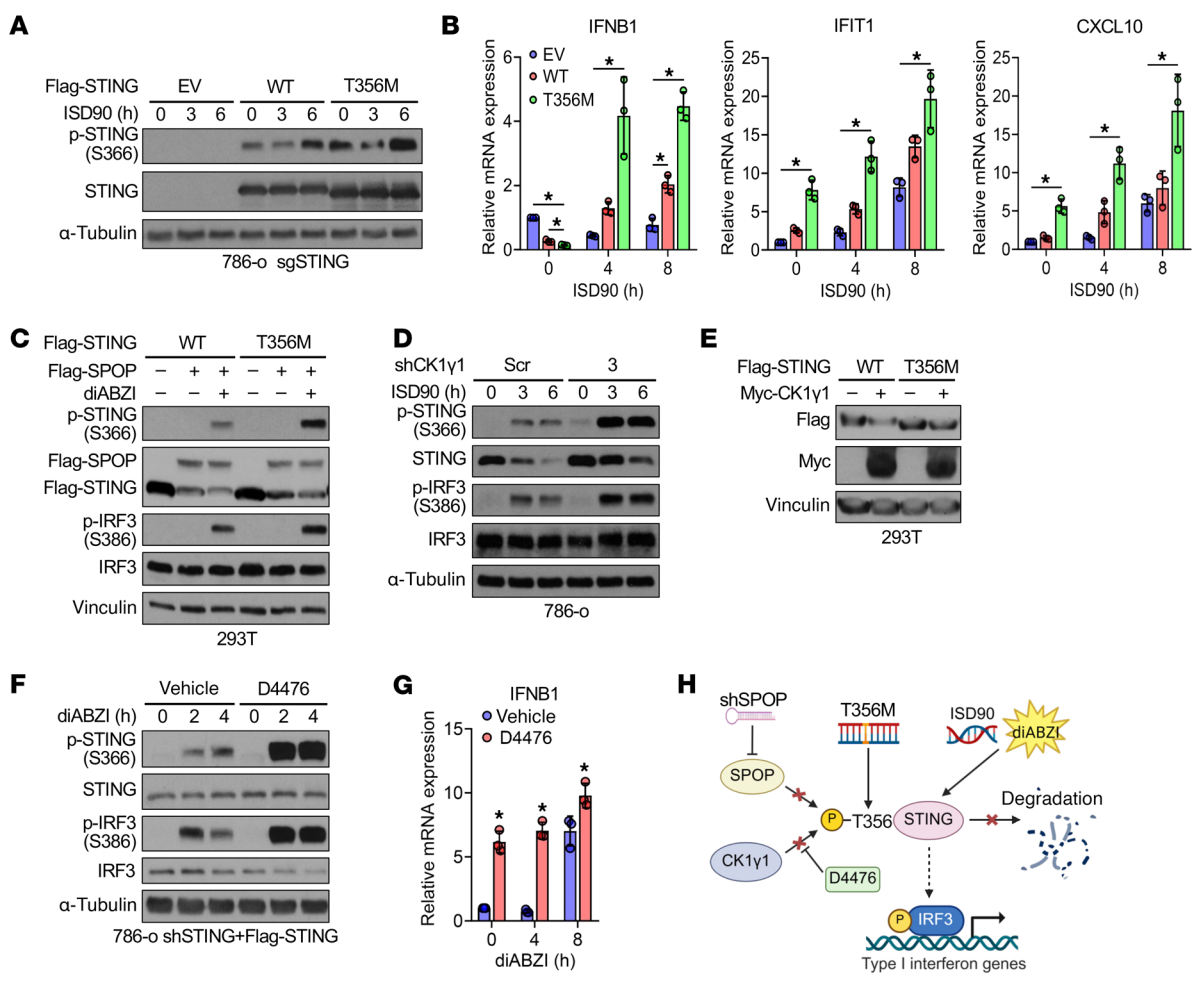
The STING-T356M mutant exhibits an enhanced ability to respond to dsDNA, owing to its evasion of SPOP-mediated degradation. (**A** and **B**) IB (**A**) and RT-PCR (**B**) analyses of indicated 786-o stable cell lines treated with 5 μg/mL of ISD90 for indicated periods. Data are shown as mean ± SD, *n* = 3. (**C**) IB analyses of 293T cells transfected with indicated constructs and treated with 3 μM diABZI for 4 h. (**D**) IB analyses of control and CK1γ1-depleted 786-o cells treated with 5 μg/mL of ISD90 for indicated periods. (**E**) IB analyses of 293T cells transfected with indicated constructs. (**F**) IB analyses of STING-reconstituted 786-o cells treated first with 40 μM D4476 for 24 h and then with 3 μM diABZI for indicated periods. (**G**) RT-PCR analyses of IFNB1 mRNA in 786-o cells treated first with 40 μM D4476 for 24 h and then with 3 μM diABZI for indicated periods. (**H**) Schematic of STING stabilization resulting from SPOP depletion, CK1γ1 inhibition, and STING-T356M mutation and increased sensitivity to DNA and STING agonist for type I interferon signaling activation. One-way ANOVA followed by Tukey’s multiple-comparison test (**B**) or 2-tailed unpaired Student’s *t* test (**G**). *P < 0.05. Representative experiments shown in figures were repeated at least 2 times independently with similar results.

To identify mutations that evade SPOP without disrupting activation, we found a T356M-STING mutation in a gastric cancer patient (Catalogue of Somatic Mutations in Cancer database; https://www.sanger.ac.uk/tool/cosmic/) that disrupted SPOP binding (Figure 5, D and E) and reduced SPOP-mediated ubiquitination (Figure 5F), extending STING half-life (Figure 5, G–I). T356M-STING enhanced ISD90- or diABZI-induced STING activation (Figure 6, A–C, and Supplemental Figure 3F). Similarly, CK1γ1 depletion or pharmacological inhibition (D4476) stabilized STING, boosting diABZI-induced activation and downstream IFN-β and ISG expression (CCL5, CXCL10, OAS1, IFIT1, and IFI44) (Figure 6, D–G, and Supplemental Figure 3, G–M). These results indicate that evasion of SPOP recognition stabilizes STING, enhancing its activation (Figure 6H).

### Pharmacological SPOP inhibition disrupts STING binding to SPOP and enhances STING activation.

**Figure 7:**
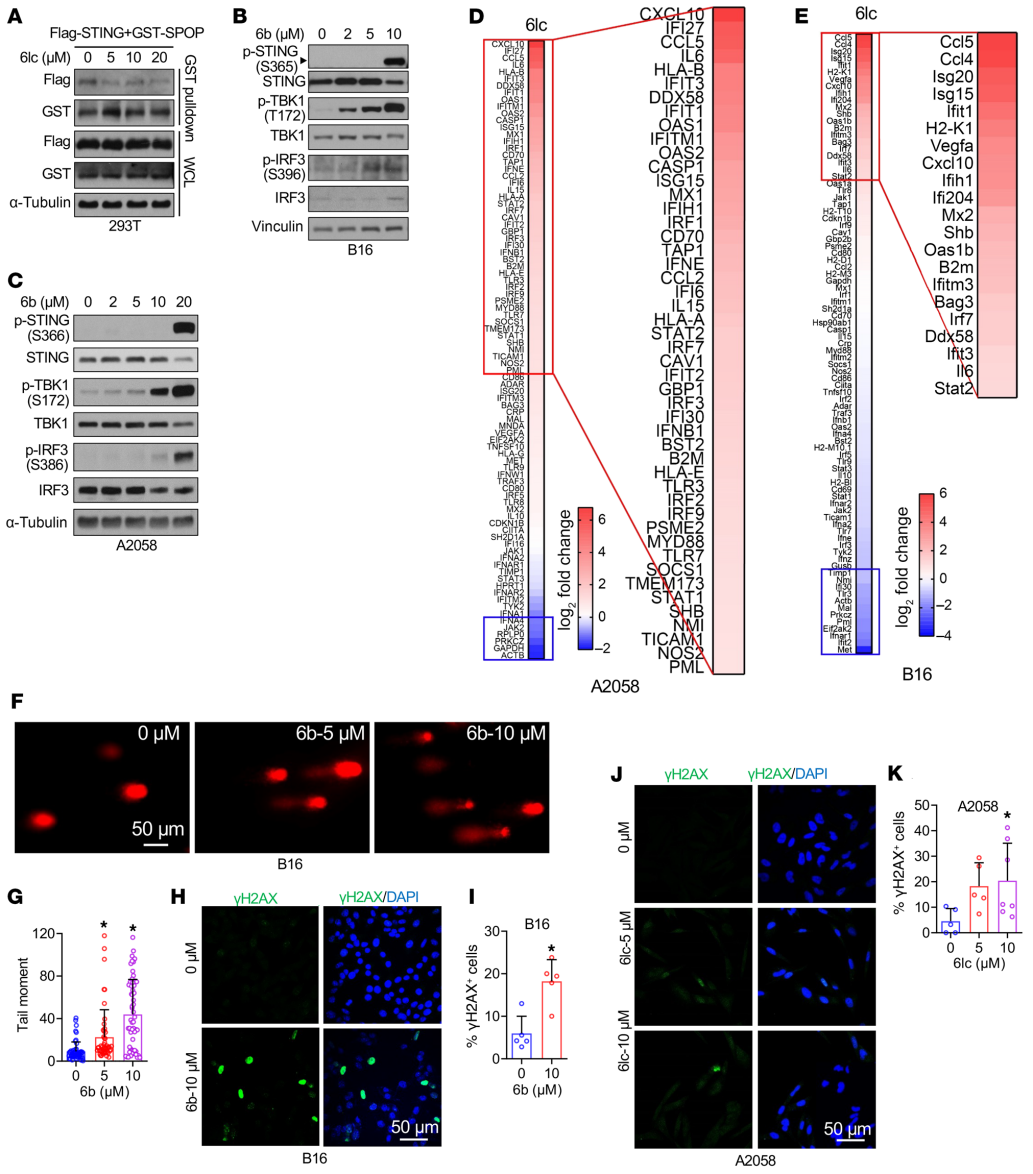
Pharmacological SPOP inhibition induces DNA damage to trigger STING activation. (**A**) IB analyses of WCL and glutathione *S*-transferase (GST) pull-down products derived from 293T cells transfected with indicated constructs and treated with indicated dose of 6lc and 10 μM MG132 for 12 h. (**B** and **C**) IB analyses of B16 and A2058 cells treated with indicated dose of 6b for 24 h. (**D** and **E**) RNA expression profiling heatmap of genes in type I interferon response in A2058 cells (**D**) and B16 cells (**E**) treated with 10 μM 6lc for 24 h. (**F** and **G**) B16 cells were treated with indicated dose of 6lc for 24 h and examined using alkaline lysis method to detect single-strand breaks. Microscopy images of representative comets (**F**) and tail moment quantification (**G**) are shown. Scale bar: 50 μm. Data are shown as mean ± SD; 0 μM, *n* = 54; 5 μM, *n* = 50; 10 μM, *n* = 56. Unpaired *t* test. *P < 0.05, compared with 0 μM. (**H**–**K**) B16 and A2058 cells were treated with indicated dose of 6b or 6lc for 24 h before immunofluorescence of γH2AX (**H** and **J**) and quantification of percentages of γH2AX positive cells (**I** and **K**). Scale bars: 50 μm. Data are shown as mean ± SD, *n* = 5–7. *P < 0.05 compared with 0 μM (unpaired *t* test). One-way ANOVA followed by Dunnett’s multiple-comparison test (**G**), Fisher’s LSD test (**K**), or 2-tailed unpaired Student’s *t* test (**I**). Representative experiments shown in figures were repeated at least 2 times independently with similar results.

Since E3 ubiquitin ligases do not exert catalytic activities but only serve to bridge E2 enzymes to specific substrates (35), a few E3 ligase inhibitors have been developed to block specific E3 ligases from binding to substrates, including Apcin (36), which blocks Cdc20/substrate interactions; Skpin (37), which blocks Skp2/p27 interactions (38); and Nutlin (39), which blocks Mdm2/p53 binding and DCAF1 inhibitors (40). Interestingly, the small molecule SPOP inhibitor SPOP-IN-6b (6b) was developed for treating kidney cancer where SPOP exerts an oncogenic function (41), and it was further upgraded to SPOP-i-6lc (6lc) (42) through medicinal chemistry optimization. Consistent with the previous study (41), we observed that 6b disrupted SPOP interactions with PTEN (Supplemental Figure 4A). We found that 6b similarly disrupted STING binding to SPOP (Supplemental Figure 4B), and 6lc was also able to do so (Figure 7A), further supporting STING as a SPOP substrate.

Treatment with 6b (Figure 7, B and C) or 6lc (Supplemental Figure 4, C and D) disrupted SPOP recognition and stabilized STING in cells. This effect was SPOP dependent (Supplemental Figure 4, E and F), ruling out off-target effects. STING stabilization was dose dependent, but at higher 6b/6lc doses, STING levels slightly decreased while activation markers pTBK1 and pIRF3 increased (Figure 7, B and C, and Supplemental Figure 4, C and D), consistent with lysosomal degradation of activated STING (43). Cytokine profiling in 6lc-treated A2058 and B16 cells (Figure 7, D and E) showed a broader and stronger ISG induction compared with SPOP genetic depletion (Figure 2B), indicating that pharmacological inhibition more effectively activates STING.

### Pharmacological SPOP inhibition induces DNA damage to trigger STING activation.

**Figure 8:**
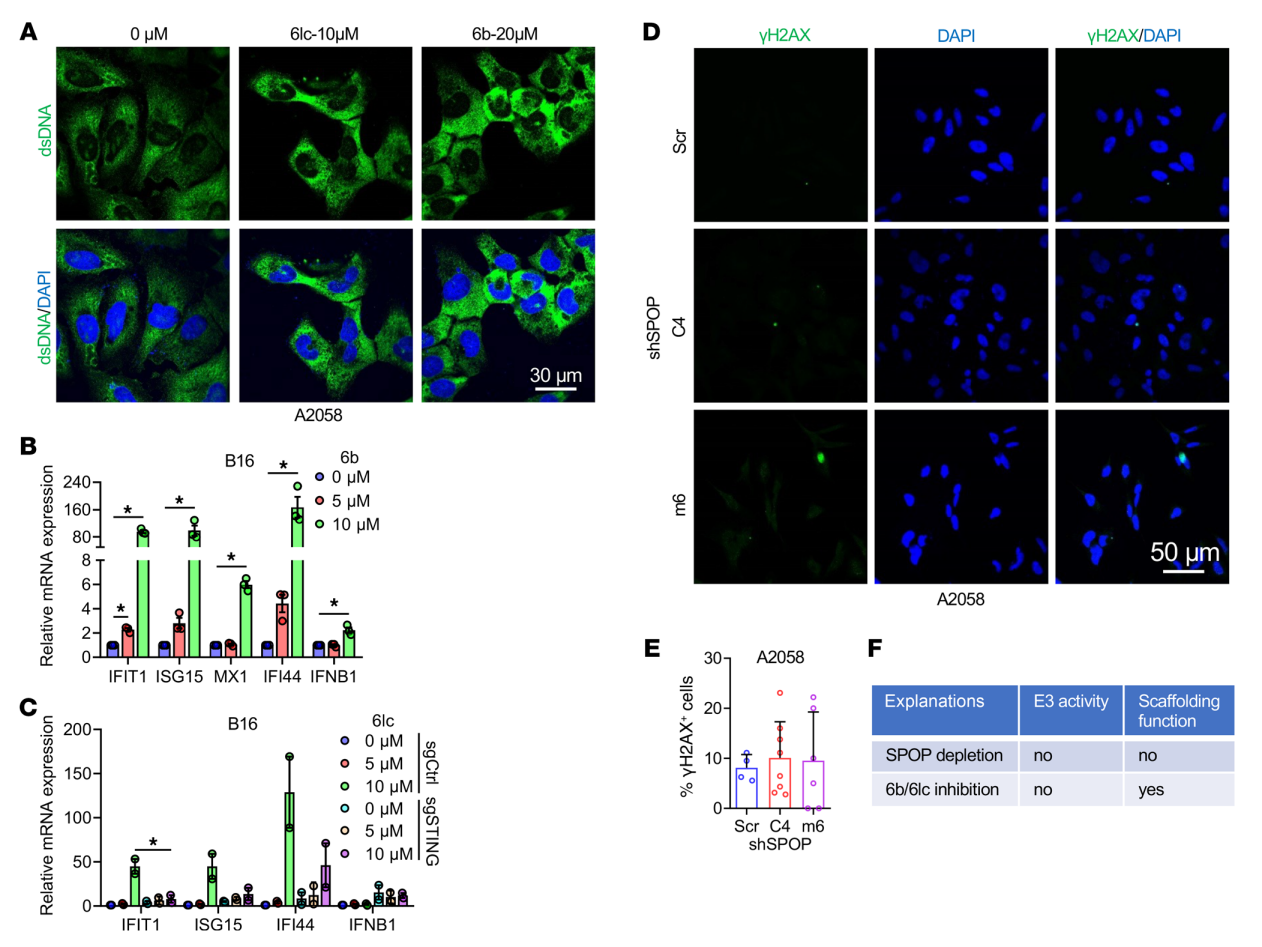
Genetic SPOP depletion mildly induces DNA damage. (**A**) Immunofluorescence of dsDNA in A2058 cells treated with indicated dose of 6b or 6lc for 24 h. Scale bar: 30 μm. (**B**) RT-PCR analyses of mRNA changes in B16 cells treated with indicated dose of 6b for 24 h. Data are shown as mean ± SD, *n* = 3. (**C**) RT-PCR analyses of mRNA changes in control and STING knockout B16 cells treated with indicated dose of 6lc for 24 h. Data are shown as mean ± SEM, *n* = 2. (**D** and **E**) Immunofluorescence of γH2AX in control and SPOP-depleted A2058 cells and quantification of percentages of γH2AX positive cells. Scale bar: 50 μm. Data are shown as mean ± SD, *n* = 4–8. Unpaired *t* test determined no statistical significance between any groups. (**F**) Schematic illustration of the impact of SPOP depletion and 6b/6lc treatment on the function of SPOP protein. One-way ANOVA followed by Fisher’s LSD test (**E**) or Tukey’s multiple-comparison test (**B** and **C**). *P < 0.05. Representative experiments shown in figures were repeated at least 2 times independently with similar results.

Canonical STING activators include viral/bacterial DNA, apoptotic cells, and damaged genomic or mitochondrial DNA (32). Treatment with 6b significantly increased genomic DNA damage, as shown by comet assays (44) (Figure 7, F and G) and γH2AX foci (Figure 7, H and I); 6lc had similar effects (Figure 7, J and K, and Supplemental Figure 4, G and H). DNA damage led to cytosolic dsDNA accumulation (Figure 8A and Supplemental Figure 4I), activating STING and inducing ISGs (Figure 8B and Supplemental Figure 4J). Although 6lc also caused mitochondrial DNA leakage (Supplemental Figure 4K), ethidium bromide–mediated depletion of mitochondrial DNA (45) did not affect STING activation (Supplemental Figure 4, L and M), indicating genomic DNA as the primary activator. Cytosolic dsRNA was unaffected (Supplemental Figure 4, N and O). Knockdown of cGAS or STING abolished 6lc-induced STING activation and ISG induction (Figure 8C and Supplemental Figure 4, P and R). Notably, SPOP genetic depletion did not increase DNA damage (Figure 8, D and E), suggesting that pharmacological inhibition, which preserves SPOP scaffolding while blocking E3 ligase activity (Figure 8F), uniquely induces DNA damage to activate cGAS/STING.

### SPOP inhibitors glue neosubstrates for SPOP-mediated ubiquitination and degradation to accumulate DNA damage.

**Figure 9:**
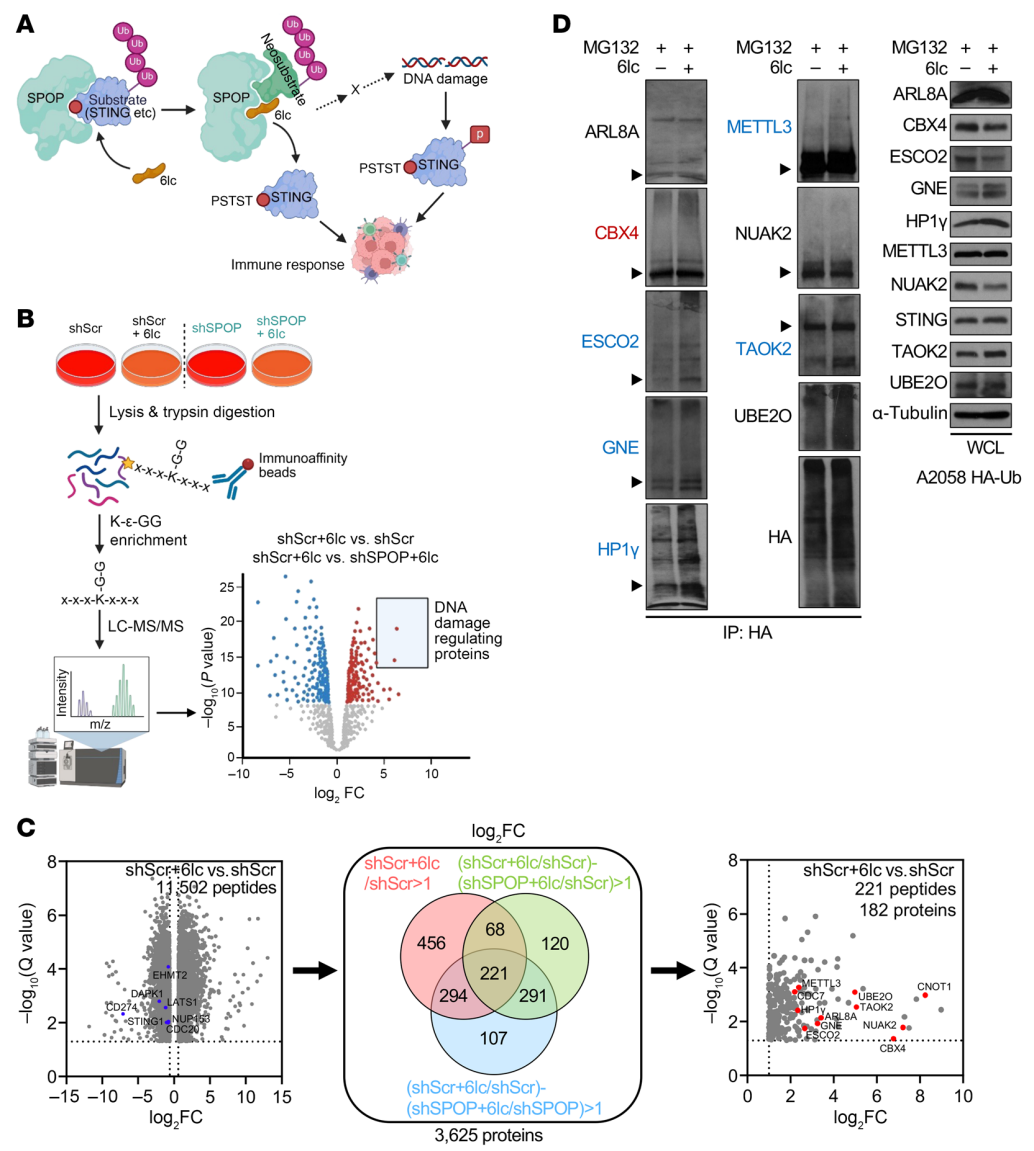
Systematic profiling of the protein degradation landscape induced by SPOP inhibition. (**A**) Schematic diagram of the hypothesis that 6lc binding to SPOP not only disrupts SPOP interactions with its bona fide substrates, resulting in STING accumulation, but also recruits neosubstrates to SPOP for regulation, which triggers DNA damage, STING activation, and immune response. (**B**) Workflow of detecting 6lc-induced protein ubiquitination. Control and SPOP-depleted A2058 cells were lysed after 12 h of treatment with 10 μM 6lc. Ubiquitinated peptides with diglycine tag resulting from trypsin digestion were enriched by K-ε-GG immunoaffinity beads, followed by quantitative LC-MS/MS analysis. Candidates regulating DNA damage were selected from 6lc-induced SPOP-dependent ubiquitinated proteins for validation. (**C**) Selection of candidates from all ubiquitinated proteins significantly changed upon 6lc treatment. Left volcano plot shows K-ε-GG peptides significantly changed (*q* value < 0.05, log_2_ fold change < −0.6 or > 0.6) in shScr cells after 6lc treatment. Hits in blue are peptides of SPOP substrates downregulated after 6lc treatment. Middle Venn diagram shows among 11,502 peptides belonging to 3,625 proteins, 221 peptides belonging to 182 proteins were at least 2-fold more enriched in shScr+6lc than in shScr, in shScr+6lc (vs. shScr) than in shSPOP+6lc (vs. shScr), and in shScr+6lc (vs. shScr) than in shSPOP+6lc (vs. shSPOP). In the right volcano plot, top hits in red with DNA damage–regulating function were selected for validation. (**D**) IB analyses of HA-IP and WCL derived from HA-Ub–expressing A2058 cells treated with 10 μM 6lc and 10 μM MG132 for 12 h. Arrowheads indicate positions of full-length proteins. Representative experiments shown in figures were repeated at least 2 times independently with similar results.

The distinct DNA damage–inducing effect of SPOP inhibitors versus genetic depletion suggested that 6b/6lc not only block SPOP’s interaction with STING, but also recruit neosubstrates for degradation (Figure 9A), similar to how lenalidomide acts as a molecular glue for CRBN (46–48). In addition, different lenalidomide derivatives recruit distinct neosubstrates for CRBN binding and degradation (49), supporting the role of E3 ligase inhibitors in controlling E3 substrate specificity. We similarly hypothesize that without 6b or 6lc, SPOP targets STING for ubiquitination and degradation. While 6b/6lc binding to SPOP may, on the one hand, block STING binding to SPOP to stabilize STING, it may, on the other hand, recruit neosubstrates to SPOP for degradation, through which 6b/6lc accumulates DNA damage to activate STING. Consistently, 6b/6lc treatment increased ubiquitinated proteins (Supplemental Figure 5A). To identify neosubstrates, we performed quantitative mass spectrometry with K-ε-GG enrichment, comparing 6lc-treated and untreated A2058 cells, using SPOP-depleted cells as controls (Figure 9B). Among 3,625 proteins with increased ubiquitination (log_2_ fold change ≥ 1), 182 showed SPOP-dependent increases (Figure 9C), enriched in chromosome organization and DNA damage response pathways (Supplemental Figure 5B). Focusing on DNA damage regulators, 6lc enhanced ubiquitination of chromobox protein homolog 4 (CBX4), ESCO2, GNE, HP1γ, METTL3, and TAOK2 (Figure 9D).

**Figure 10:**
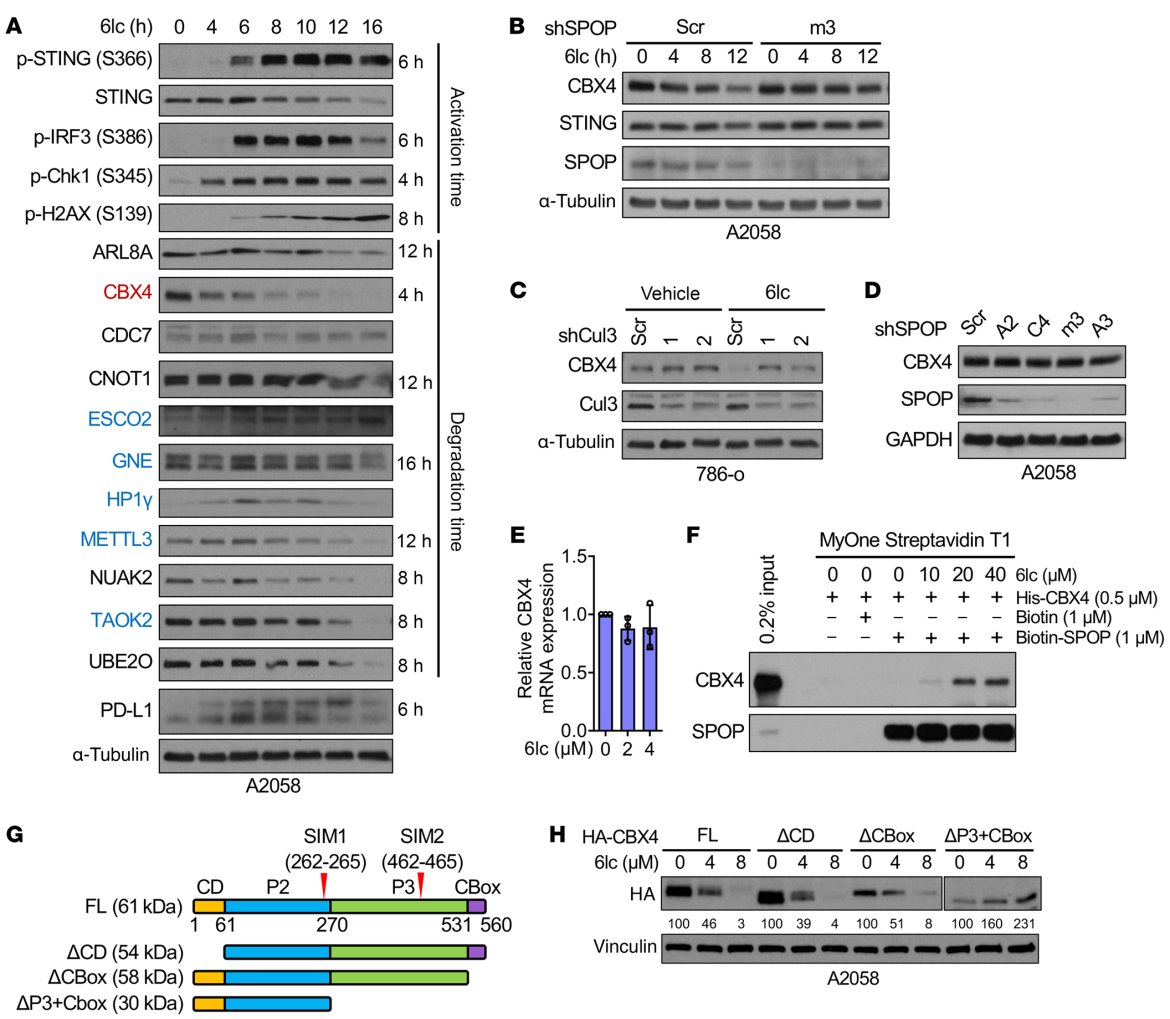
CBX4 is a neosubstrate for SPOP/6lc to control DNA damage. (**A**) IB analyses of A2058 cells treated with 20 μM 6lc for indicated periods. On the right side are starting time points of protein level changes. (**B**) IB analyses of control and SPOP-depleted A2058 cells treated with 20 μM 6lc for indicated periods. (**C**) IB analyses of control and CUL3-depleted 786-o cells treated with 20 μM 6lc for 12 h. (**D**) IB analyses of control and SPOP-depleted A2058 cells. (**E**) RT-PCR analyses of A2058 cells treated with 20 μM 6lc for indicated periods. Data are shown as mean ± SD, *n* = 3. No statistical significance between any groups (2-tailed unpaired Student’s *t* test). (**F**) In vitro streptavidin pull-down assay using indicated dose of compounds and purified proteins. (**G**) Schematic of CBX4 backbone, SUMO-interacting motifs SIM1 and SIM2, and truncations used in **H**. Full-length CBX4 (FL) consists of a chromodomain (CD), the 2 intrinsically disordered domains P2 and P3, and a CBox domain. (**H**) IB analyses of HA-CBX4-FL and truncations in A2058 cells upon 20 μM 6lc treatment for indicated periods. Quantification of relative HA grayscale values is shown. Representative experiments shown in figures were repeated at least 2 times independently with similar results.

We further hypothesized that SPOP/6lc-induced degradation of the true hit(s) would cause DNA damage prior to STING activation. Time-course analysis revealed only CBX4 degradation preceded STING activation in 6lc-treated A2058 and 786-o cells (Figure 10A and Supplemental Figure 5, C and D). 6lc promoted CBX4 K48-linked ubiquitination and proteasomal degradation in a SPOP/Cul3-dependent manner (Figure 10, B and C, and Supplemental Figure 5F). CBX4 protein was not affected by SPOP depletion (Figure 10D), indicating CBX4 is not a natural SPOP substrate, nor was it affected by 6lc (Figure 10E), ruling out transcriptional regulation. In vitro pulldown confirmed that SPOP binds CBX4 only in the presence of 6lc (Figure 10F and Supplemental Figure 5, G and H). The CBX4-K114R mutant resisted 6lc-induced ubiquitination and degradation (Supplemental Figure 5, I and J).

CBX4, a SUMO E3 ligase in PRC1 (50), uses its chromodomain and SUMO-interacting domains (SIMs) for function (Figure 10G). Truncation of its C-terminal region abolished 6lc-mediated degradation (Figure 10H), suggesting this region contains the neodegron. Together, these results support CBX4 as a bona fide neosubstrate for SPOP/6lc, revealing that 6lc functions as a molecular glue that recruits CBX4 to SPOP for degradation, which in turn induces DNA damage.

### CBX4 is a neosubstrate for SPOP/6lc to control DNA damage response.

**Figure 11:**
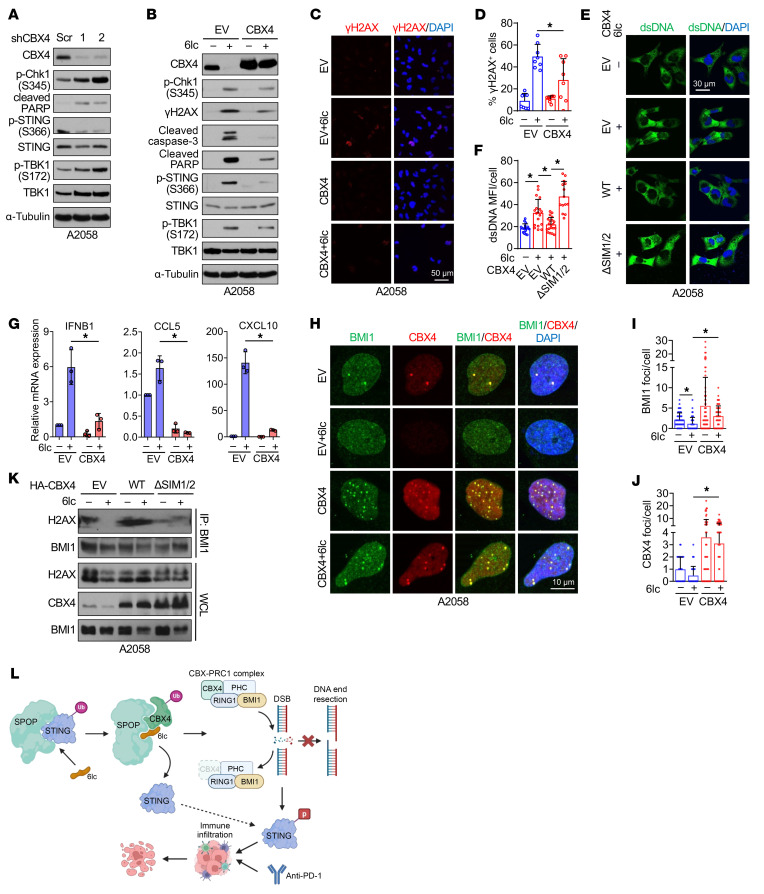
6lc triggers DNA damage through the CBX4/BMI1/H2A axis. (**A**) IB analyses of control and CBX4-depleted A2058 cells. (**B**) IB analyses in control and CBX4-overexpressing A2058 cells treated with 20 μM 6lc for 12 h. (**C**, **E**, **G**, and **H**) γH2AX immunofluorescence (**C**), dsDNA immunofluorescence (**E**), RT-PCR analyses (**G**), and BMI1/CBX4 immunofluorescence (**H**) in control and CBX4-overexpressing A2058 cells treated with 20 μM 6lc for 12 h. Scale bars: 50 μm (**C**), 30 μm (**E**), 10 μm (**H**). (**D** and **F**) Quantifications of rH2Ax foci (**D**) from **C** and dsDNA MFI cells (**F**) from **E**. (**I** and **J**) Quantifications of BMI1 (**I**) or CBX4 (**J**) foci from **H**. Data are shown as mean ± SD; *n* = 8 (**D**), *n* = 14–21 (**F**), *n* = 3 (**G**), and *n* = 55–65 (**I** and **J**). (**K**) IB analyses of BMI1-IP and WCL derived from A2058 cells overexpressing CBX4-WT and -ΔSIM1/2. (**L**) A schematic diagram of 6lc-mediated CBX4 degradation, DNA damage, and STING activation. DSB, double-strand break; PHC, polyhomeotic homolog. Detailed information is given in Results. One-way ANOVA followed by Tukey’s multiple-comparison test (**D**, **F**, and **G**) or Kruskal-Wallis test followed by Dunn’s test (**I** and **J**). *P < 0.05. Representative experiments shown in figures were repeated at least 2 times independently with similar results.

We next tested whether SPOP/6lc-induced CBX4 degradation triggers DNA damage. CBX4 depletion increased DNA damage, as shown by elevated pChk1 levels and γH2AX signals, as well as cytosolic dsDNA/cGAS foci (Figure 11A and Supplemental Figure 6, A–C). Ectopic CBX4 partially rescued 6lc-induced CBX4 degradation, DNA damage, and cytosolic dsDNA accumulation (Figure 11, B–F, and Supplemental Figure 6, D and E), reducing STING activation and ISG production (IFN-β, CCL5, and CXCL10; Figure 11G). Conversely, CBX4-depleted cells were resistant to 6lc-induced DNA damage and dsDNA accumulation (Supplemental Figure 6, F–H), supporting CBX4 as a key SPOP/6lc neosubstrate that mediates STING activation.

CBX4, besides promoting cancer progression (51) and metastasis (52), maintains genome stability via SUMOylation of BMI1 (53), which recruits BMI1 to DNA damage foci (54) for repair (55). Consistently, 6lc reduced CBX4 and BMI1 foci, while ectopic CBX4 restored BMI1 foci and H2AX interactions (Figure 11, H–K). A SUMO E3 ligase–deficient CBX4 mutant (ΔSIM1/2) failed to rescue BMI1 recruitment (Figure 11K and Supplemental Figure 6, I–K). These data support a model in which 6lc both stabilizes STING by blocking SPOP binding and glues CBX4 to SPOP for degradation, impairing BMI1-mediated DNA repair. The resulting DNA damage activates STING, promoting tumor innate immunity and enhancing immune cell infiltration to improve therapeutic responses (Figure 11L).

### SPOP inhibition enhances the antitumor effects of checkpoint blockades and CAR T cells.

**Figure 12:**
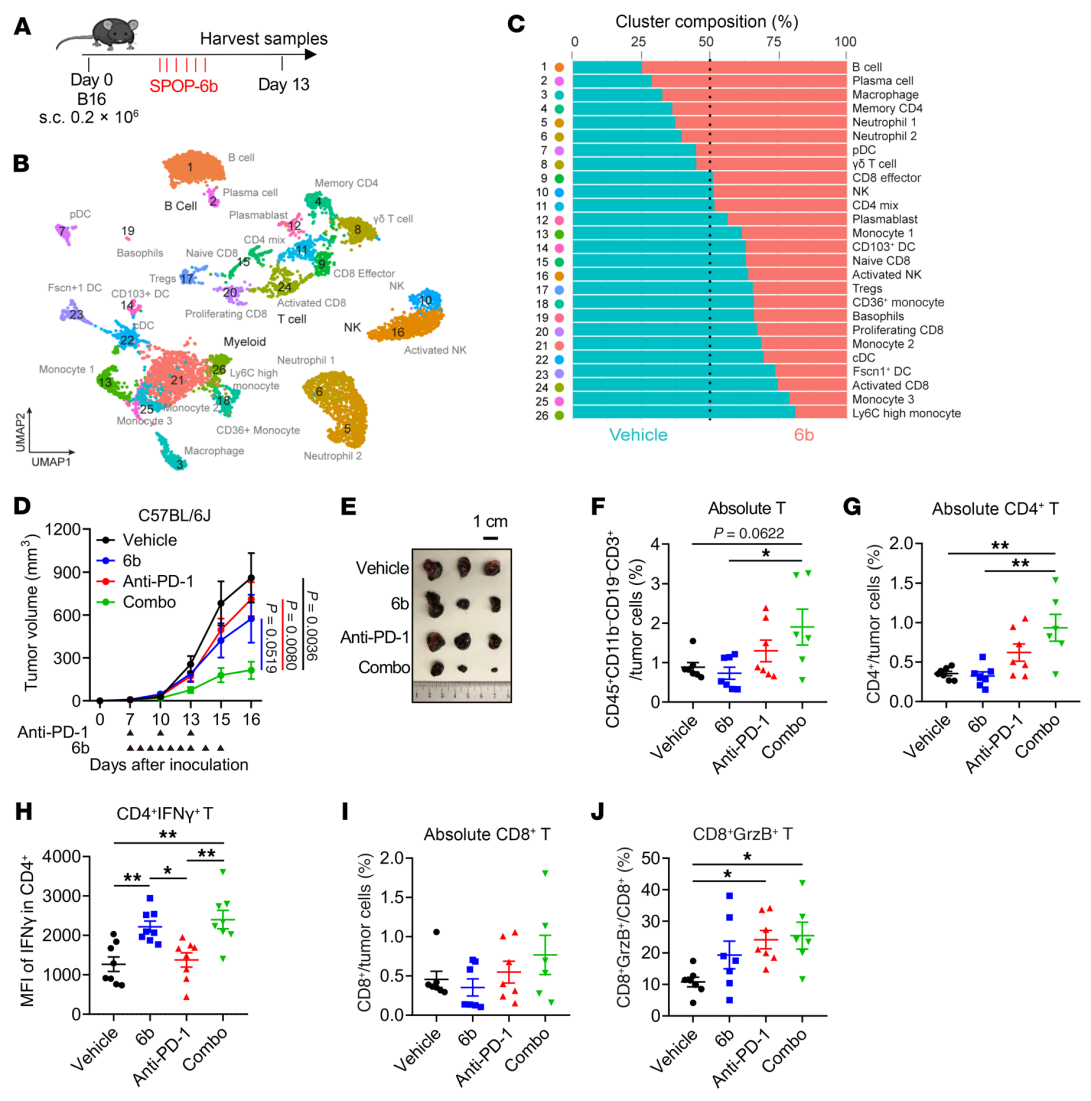
SPOP inhibition enhances immunotherapy effects in murine models. (**A**) Schematic of the syngeneic B16 melanoma model in which tumor-bearing mice were treated with/without 6b for scRNA-seq analysis. (**B**) UMAP plot of cells profiled from 2 groups; clusters are annotated based on expression patterns of characteristic genes. (**C**) Composition of each cluster from **A**. (**D**) Tumor volume measurements at indicated days after cell inoculation. Arrowheads indicate treatment schedule of indicated agents. Data are shown as mean ± SEM. Vehicle, 6b, and anti–PD-1: *n* = 9; Combo: *n* = 8. (**E**) Representative images of tumors isolated from **D**. Scale bar: 1 cm. (**F**–**J**) The absolute percentages of T cells (**F**), CD4^+^ T cells (**G**), MFI of IFN-γ in CD4^+^ cells (**H**), CD8^+^ T cells (**I**), and percentage of Granzyme B^+^ (GrzB^+^) cells in CD8^+^ T cells (**J**) in implanted B16 tumors from mice treated with indicated agents were analyzed by flow cytometry. Data are shown as mean ± SEM; *n* = 8 (**H**); vehicle, 6b, and anti–PD-1: *n * = 7 (**F**, **G**, **I**, and **J**); Combo: *n* =  6 (**F**, **G**, **I**, and **J**). Two-way (**D**) or one-way ANOVA (**F**–**J**) followed by Tukey’s multiple-comparison test. *P < 0.05, **P < 0.01.

ICBs are pivotal in metastatic melanoma (56, 57), but response rates are influenced by tumor-infiltrating lymphocytes and the tumor microenvironment (58). Since SPOP inhibition stabilizes and activates STING, triggering IFN and ISG production, which could promote immune cell infiltration (59), we evaluated whether SPOP inhibitors enhance ICB efficacy in melanoma models. Using the anti–PD-1–resistant B16 C57BL/6 model (60, 61), mice were treated daily with the SPOP inhibitor 6b and tumors collected on day 13 for scRNA-seq of FACS-sorted CD45^+^ cells (Figure 12A). The 6b treatment increased B cells (cluster 1), plasma cells (cluster 2), macrophages (cluster 3), and memory CD4^+^ T cells (cluster 4), which are associated with anti–PD-1 response (Figure 12, B and C, and Supplemental Figure 7, A and B) (62–64). CD8^+^ populations were largely unchanged, but PD-L1 expression was upregulated in plasma cells, naive CD8^+^ T cells, macrophages, and CD36^+^ monocytes (Supplemental Figure 7C).

Functionally, 6b or anti–PD-1 alone showed limited efficacy, whereas the combination significantly reduced tumor growth with minimal toxicity (Figure 12, D and E, and Supplemental Figure 8A). FACS and IHC analyses confirmed enrichment of intratumor CD4^+^ T cells, particularly IFN-γ^+^ activated cells (Figure 12, F–H, and Supplemental Figure 8B), and increased activated CD8^+^ (GrzB^+^) T cells (Figure 12, I and J). Although macrophages increased, 6b shifted polarization from M1 to M2 (Supplemental Figure 8, C–G), suggesting macrophage changes do not mediate the antitumor effect. Similar results were observed using the SPOP inhibitor 6lc (Supplemental Figure 8, H and I).

**Figure 13:**
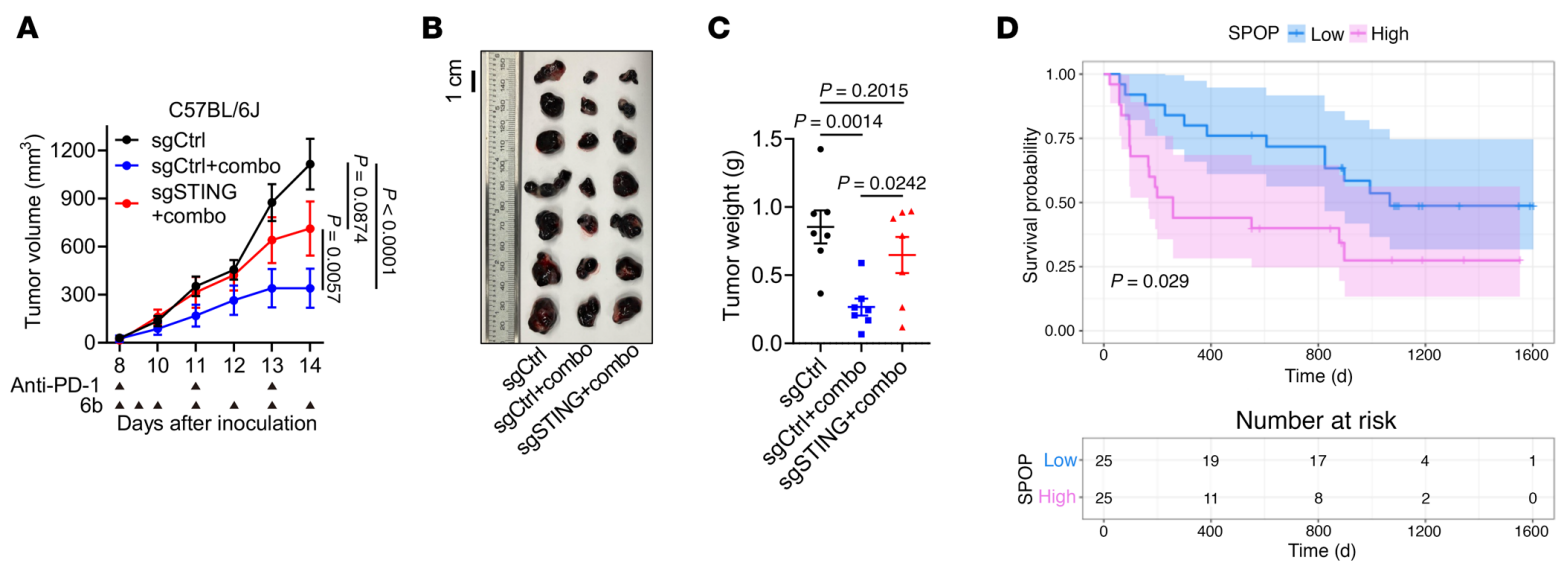
The enhanced immunotherapeutic effects of SPOP inhibition depend on tumor-intrinsic STING. (**A**) Tumor volume measurements at indicated days after cell inoculation. Arrowheads indicate treatment schedule of indicated agents. Data are shown as mean ± SEM, *n* = 7. (**B** and **C**) Representative images of tumors (**B**) isolated from **A** and tumor weight (**C**). Scale bar: 1 cm. (**D**) Kaplan-Meier survival curve of anti–PD-1–treated melanoma patients with high or low expression of SPOP mRNA. The image is based on the SPOP-Melanoma-PRJEB23709_anti-PD-1-None-None-0.5-survival dataset in the Tumor Immunotherapy Gene Expression Resource database (http://tiger.canceromics.org/#/). Two-way ANOVA followed by Tukey’s multiple-comparison test (**A**) or 1-way ANOVA followed by Fisher’s LSD test (**C**).

Importantly, the combination of 6b and anti–PD-1 markedly inhibited WT-B16 tumor growth, but not STING-depleted tumors (Figure 13, A–C), indicating that 6b’s antitumor effect depends on SPOP/STING signaling. Analysis of the Tumor Immunotherapy Gene Expression Resource further showed that melanoma patients with high SPOP expression had poorer responses to anti–PD-1 therapy (Figure 13D), likely due to reduced STING levels and lower tumor-infiltrating immune cells.

**Figure 14:**
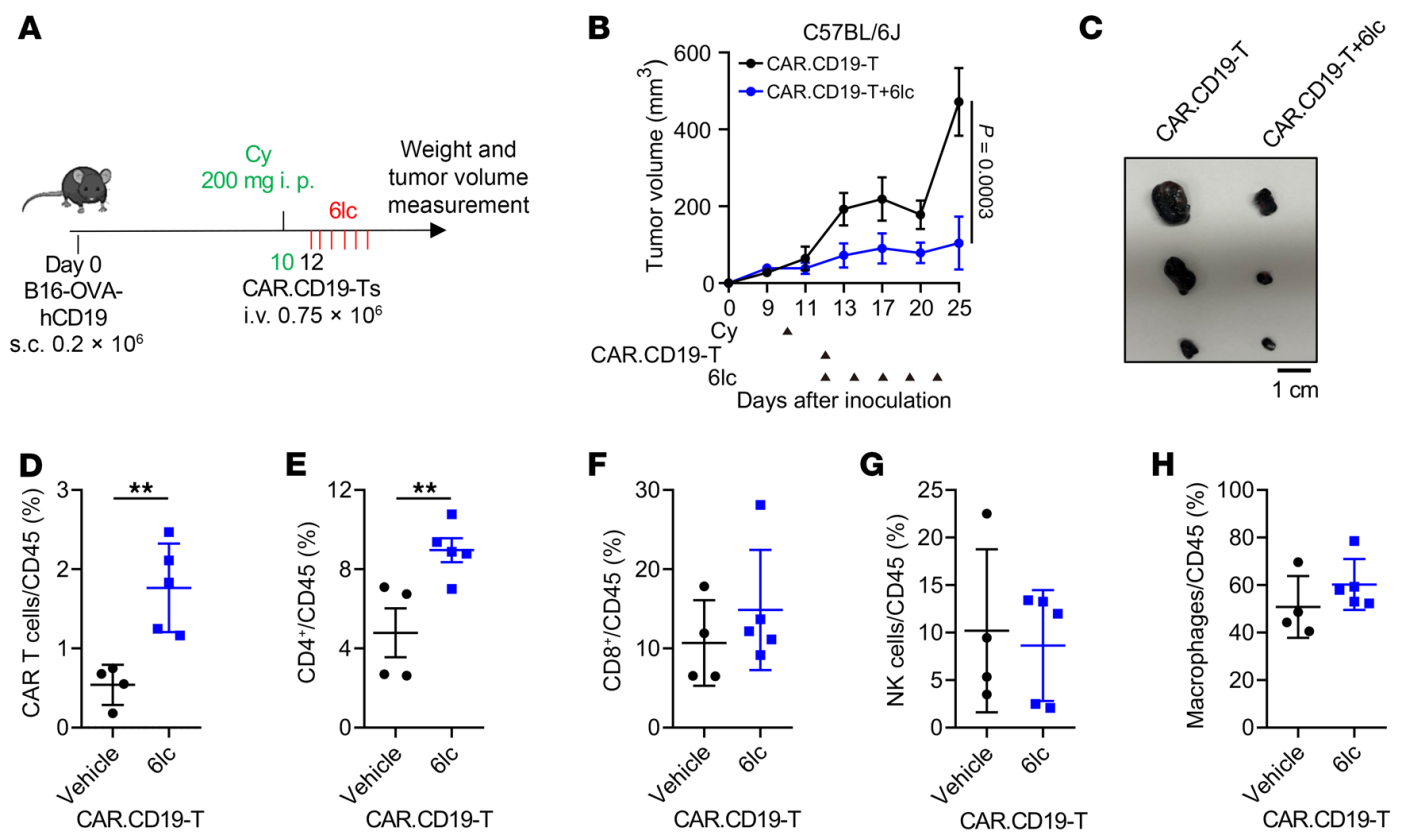
SPOP inhibition enhances CAR T cell effects in xenografted B16 melanoma models. (**A**) Schematic of the B16-OVA-hCD19 melanoma model in which tumor-bearing mice were lymphodepleted with cyclophosphamide (Cy) and then treated with CD19-CAR T cells intravenously and following treatment with/without 6lc, 5 times every 2–3 days. (**B**) Measurement of the tumor volume at indicated days after cell inoculation. Arrowheads indicate treatment schedule of indicated cells and agents. Data are shown as mean ± SEM. CAR.CD19-T: *n* = 4; CAR.CD19-T+6lc, *n* = 5 (2-way ANOVA followed by Tukey’s multiple-comparison test). (**C**) Representative images of tumors isolated from **B**. Scale bar: 1 cm. (**D**–**H**) Percentages of CAR T (**D**), CD4^+^ (**E**), CD8^+^ (**F**), NK (**G**), macrophages (**H**) in CD45^+^ cells from B16-OVA-hCD19 tumors in **B**. Data are shown as mean ± SEM. Vehicle: *n* = 4; 6lc: *n* = 5. Two-tailed unpaired Student’s *t* test. **P < 0.01.

Using the B16-OVA-hCD19 model (61, 65–67), we tested CAR.CD19-T cell therapy with or without 6lc (5 doses, 20 mg/kg) (Figure 14A). The combination of 6lc and CAR.CD19-T cells markedly improved tumor control (Figure 14, B and C) and increased infiltration of both CAR T and CD4^+^ T cells (Figure 14, D–H). These results indicate that SPOP inhibition enhances the efficacy of both ICB and CAR T therapies by promoting CD4^+^ T cell–mediated antitumor immunity, highlighting its potential to boost diverse immunotherapy approaches.

## Discussion

We identified the E3 ligase SPOP as a suppressor of tumor immunity by promoting ubiquitination and degradation of the innate immune sensor STING in melanoma and RCC. Pharmacological SPOP inhibitors 6b and 6lc not only disrupt the SPOP/STING interaction, but also act as molecular glues, inducing degradation of neosubstrates. Global K-ε-GG enrichment and liquid chromatography–tandem mass spectrometry (LC-MS/MS) analyses revealed CBX4 as a neosubstrate of the SPOP/6lc complex. SPOP-dependent CBX4 degradation impairs DNA repair by disrupting CBX4-mediated SUMOylation and BMI1 recruitment, leading to DNA damage and activation of the cGAS/STING pathway, which in turn stimulates innate immune responses. scRNA-seq of 6b-treated B16 xenografts showed increased infiltration of immune cells linked to anti–PD-1 responsiveness. Consistently, SPOP inhibition synergized with anti–PD-1 therapy to suppress tumor growth via enhanced CD4^+^IFN-γ^+^ T cell infiltration and further boosted CAR.CD19-T efficacy in B16-OVA tumors. These findings establish a molecular glue function for SPOP inhibitors and highlight their promise as immunotherapy adjuvants.

Targeted protein degradation is an emerging therapeutic strategy (68). Unlike proteolysis-targeting chimeras, which are rationally designed by linking ligands for a protein of interest and an E3 ligase (69), molecular glue degraders are typically discovered serendipitously. Their smaller size confers better delivery, oral bioavailability, and pharmacodynamics. Although approximately 20 molecular glues have been identified (69), most were found through random screening, as their rational design remains challenging (68). Known molecular glues primarily act through E3 ligases such as DCAF15 (70, 71), DDB1 (72–74), and β-TRCP (75), which promote E3/substrate complex formation. For instance, the β-TRCP glue enhances β-TRCP/β-catenin interaction (75). Whether CRBN-, DCAF15-, or DDB1-associated glues also disrupt native substrate binding remains unclear. Our findings reveal that SPOP inhibitors 6b and 6lc act through a distinct mechanism, simultaneously blocking endogenous substrate binding while recruiting neosubstrates. As SPOP is a cullin 3 E3 ligase, unlike the cullin 1/4 ligases targeted previously (35), these compounds expand the landscape of molecular glue degraders.

The STING agonist 2′3′-cGAMP has been shown to improve anti–PD-1 efficacy in B16 melanoma models (76). Melanoma is generally immune cold, and predictors of anti–PD-1 response include BRCA2 mutations and the IPRES transcriptional signature, rather than mutation burden (77). Combination therapies enhancing CD8^+^ T cell infiltration or PD-L1 expression improve anti–PD-1 efficacy (78).

Enhancing CD8^+^ T cell infiltration and tumor PD-L1 expression typically augments anti–PD-1 responses. Here, pharmacological SPOP inhibition stabilizes and activates tumor STING, driving infiltration of CD4^+^, but not CD8^+^, T cells, thereby improving anti–PD-1 efficacy in B16 melanoma. Although SPOP inhibition increases M2 rather than M1 macrophages, this immunosuppressive shift is counterbalanced by enhanced effector T cell infiltration. While CD4^+^ T cells are traditionally considered helpers for cytotoxic T lymphocyte activation, they can also produce effector cytokines, such as IFN-γ, to directly mediate tumor cell killing (79). This mechanism appears to underlie the antitumor effects of 6b/6lc observed in our study. Additionally, CD4^+^ T cells can drive humoral immune responses by promoting B cell differentiation and maturation into affinity-matured, class-switched plasma cells (80, 81), consistent with our scRNA-seq analysis showing increased B and plasma cell populations following 6b treatment. The capacity of CD4^+^ T cells to suppress tumors independently of CD8^+^ T cells through inflammatory cell death has been reported previously (82). Moreover, in a B16-OVA tumor model, SPOP inhibition similarly enhances CD4^+^ CAR.CD19-T cell tumor infiltration, resulting in improved tumor control. Collectively, these findings suggest that SPOP inhibition augments CD4^+^ T cell–mediated antitumor immunity and support further evaluation of SPOP inhibitors in clinical settings.

## Methods

### Sex as a biological variable.

Only female mice were used in murine melanoma models to ensure data reproducibility. Sex was not considered as a biological variable, as melanoma occurs in both sexes in humans.

### Cell culture and transfection.

Human RCC cell lines 786-o (from Qing Zhang, UT Southwestern, Dallas, Texas), A498, Caki-1 (ATCC), RCC10, and UMRC6; mouse RCC line Renca (from William Kim, The University of North Carolina at Chapel Hill); human kidney cell lines HEK293 and HEK293T (ATCC); human melanoma lines A2058, HMCB, and MeWo (from Deborah DeRyckere, Emory University, Atlanta, Georgia); and mouse melanoma lines B16 and B16-OVA (generated in-house) were cultured in DMEM with 10% FBS, 100 U/mL penicillin, and 100 μg/mL streptomycin.

Cells were transfected using Lipofectamine 3000 (L3000150, Thermo Fisher Scientific) or polyethylenimine (23966, Polysciences) as described (83, 84). Lentiviral packaging, infection, and selection were performed as previously reported (83, 84), using 200 μg/mL hygromycin (H3274, Sigma-Aldrich) or 2 μg/mL puromycin (BP2956100, Fisher BioReagents). Compounds used include 2′3′-cGAMP (tlrl-nacga23-02, InvivoGen), diABZI (28054, Cayman), D4476 (HY-10324), epiblastin A (HY-114858), 6b (HY-122615, MedChemExpress), 6lc (Tocris 7498), Bafilomycin A1 (S1413), and cycloheximide (S6611, Selleck).

### Plasmids.

Flag-STING constructs (WT, 4A, P352A, S353A, S355F, T356M) and Flag-mSTING (WT, 2A) were generated by overlap PCR and cloned into pcDNA3.0. pBabe-Flag-STING (WT, 4A, S355F) and pLenti-Flag-STING (WT, T356M) were made by subcloning respective inserts into pBabe-hygro or pLenti-hygro vectors. HA-CBX4 constructs (WT, ΔSIM1/2, ΔCD, ΔCBox) were generated by overlap PCR from CBX4 cDNA (provided by Virginia Byers Kraus, Duke University, Durham, North Carolina) and cloned into pLenti-GFP-hygro. CMV-GST-CBX4 (WT, K114R) and pET-28a-CBX4 were cloned into CMV-GST and pET-28a vectors, respectively. Flag-, HA-, and GST-SPOP were cloned into pcDNA3-Flag, pcDNA3-HA, and CMV-GST vectors. His-SUMO-avi-SPOP (aa 28–359) was cloned into pExp-His-Sumo-TEV. Flag-cGAS (85); HA-Ub, His-Ub-WT, and K48-Ub (85, 86); Myc-CUL3 and CK1/CK2 (7); and Myc-PTEN (87) were described previously. pRSET-6xTR-TUBE was from Addgene (catalog 110313).

### Primers.

The following primers were used: STING-BamHI-F: GACACCGACTCTAGAGGATCCATGCCCCACTCCAGCCTGCA; STING-SalI-Flag-R: ATCCAGAGGTTGATTGTCGACTCACTTGTCGTCATCGTCTTTGTAGTCAGAGAAATCCGTGCGGAGAG; mSTING-BglII-F: GCATAGATCTATGCCATACTCCAACCTGCA; mSTING-SalI-Flag-R: GCATGTCGACTCACTTGTCGTCATCGTCTTTGTAGTCGATGAGGTCAGTGCGGAGTG; STING-4A-F: AGACCTCAGCGGTGCCCGCTGCCGCCGCGATGTCCCAAGAGCCTGA; STING-4A-R: TCAGGCTCTTGGGACATCGCGGCGGCAGCGGGCACCGCTGAGGTCT; STING-P352A-F: TGAAGACCTCAGCGGTGGCCAGTACCTCCACGATG; STING-P352A-R: CATCGTGGAGGTACTGGCCACCGCTGAGGTCTTCA; STING-S353A-F: AGACCTCAGCGGTGCCCGCTACCTCCACGATGTCCC; STING-S353A-R: GGGACATCGTGGAGGTAGCGGGCACCGCTGAGGTCT; STING-S355F-F: AGCGGTGCCCAGTACCTTCACGATGTCCCAAGAGC; STING-S355F-R: GCTCTTGGGACATCGTGAAGGTACTGGGCACCGCT; STING-T356M-F: GGTGCCCAGTACCTCCATGATGTCCCAAGAGCCTG; STING-T356M-R: CAGGCTCTTGGGACATCATGGAGGTACTGGGCACC; mSTING-2A-F: CAGTGGCACCTCCTCCCGCCGTACTGGCCCAAGAGCCAAGACTC; mSTING-2A-R: GAGTCTTGGCTCTTGGGCCAGTACGGCGGGAGGAGGTGCCACTG; SPOP-BamHI-F: GCATGGATCCATGTCAAGGGTTCCAAGTCC; SPOP-SalI-R: GCATGTCGACTTAGGATTGCTTCAGGCGTT; BstBI-Avi-tag-SPOP-F: GCATTTCGAAGGCCTGAATGACATCTTTGAGGCCCAGAAGATCGAGTGGCATGAGAAGGTAGTGAAATTCTCCTA; XhoI-SPOP-R: GCATCTCGAGTTATGCTGAAGCCAGAGAGC; CBX4-BglII-F: GCATAGATCTGAGCTGCCAGCTGTTGG; CBX4-SalI-R: GCATGTCGACCTACACCGTCACGTACTCC; CBX4-delSIM1-F: AGAACAAGAACGGACGCATGAGCAAATACATGGA; CBX4-delSIM1-R: TCCATGTATTTGCTCATGCGTCCGTTCTTGTTCT; CBX4-delSIM2-F: CCCTCCCGCAGCCCGAGGACTCAGACCTGGATGA; CBX4-delSIM2-R: TCATCCAGGTCTGAGTCCTCGGGCTGCGGGAGGG; CBX4-delCD(1-60)-BglII-F: GCATAGATCTGAACGGCAGGAGCAGCTGAT; CBX4-delCBox(531-560)-SalI-R: GCATGTCGACCAGCGACTCTGCAGGTTCGT; CBX4-delCBox+P3(270-560)-SalI-R: GCATGTCGACACCGCCTGCATGCCGTTCTCCATGTATTTGCTCATCACGA; CBX4-K114R-F: TTTGGGCGCGCAGGGGAGGGGCCAGGGGCATCAGT; and CBX4-K114R-R: ACTGATGCCCCTGGCCCCTCCCCTGCGCGCCCAAA.

RT-PCR primers are as follows: CBX4-F: ACCGTGCCAAGCTGGATTT; CBX4-R: AGGTCGTACATTTTGGGGTCG; CCL5-F: TGCCCACATCAAGGAGTATTT; CCL5-R: CTTTCGGGTGACAAAGACG; CSNK1G1-F: CCCACAGGTGTATTACTTTGGAC; CSNK1G1-R: GTAAATGTTCGGTCACAGAGGT; CXCL10-F: GGCCATCAAGAATTTACTGAAAGCA; CXCL10-R: TCTGTGTGGTCCATCCTTGGAA; mCXCL10-F: CCAAGTGCTGCCGTCATTTTC; mCXCL10-R: GGCTCGCAGGGATGATTTCAA; mDLOOP1-F: CCCTTCCCCATTTGGTCT; mDLOOP1-R: TGGTTTCACGGAGGATGG; mDLOOP2-F: CCCTTCCCCATTTGGTCT; mDLOOP2-R: TGGTTTCACGGAGGATGG; mGAPDH-F: AGGTCGGTGTGAACGGATTTG; mGAPDH-R: GGGGTCGTTGATGGCAACA; IFI44-F: TTTTCGATGCGAAGATTCACTGG; IFI44-R: CCTGATGCGTTACATGCCCTT; mIFI44-F: ATGCTCCAACTGACTGCTCG; mIFI44-R: ACAGCAATGCCTCTTGTCTTT; IFIT1-F: AGAAGCAGGCAATCACAGAAAA; IFIT1-R: CTGAAACCGACCATAGTGGAAAT; mIFIT1-F: ATCGCGTAGACAAAGCTCTTC; mIFIT1-R: GTTTCGGGATGTCCTCAGTTG; IFNB1-F: ATGACCAACAAGTGTCTCCTCC; IFNB1-R: GGAATCCAAGCAAGTTGTAGCTC; mIFNB1-F: AGCTCCAAGAAAGGACGAACA; mIFNB1-R: AGCTCCAAGAAAGGACGAACA; mISG15-F: GGTGTCCGTGACTAACTCCAT; mISG15-R: CTGTACCACTAGCATCACTGTG; mMX1-F: GACCATAGGGGTCTTGACCAA; mMX1-R: AGACTTGCTCTTTCTGAAAAGCC; OAS1-F: TGTCCAAGGTGGTAAAGGGTG; OAS1-R: CCGGCGATTTAACTGATCCTG; mPLOG1-F: GATGAATGGGCCTACCTTGA; mPLOG1-R: TGGGGTCCTGTTTCTACAGC; SPOP-F: GCCCTCTGCAGTAACCTGTC; SPOP-R: GTCTCCAAGACATCCGAAGC; STING1-F: CACTTGGATGCTTGCCCTC; STING1-R: GCCACGTTGAAATTCCCTTTTT; mTERT-F: CTAGCTCATGTGTCAAGACCCTCTT; mTERT-R: GCCAGCACGTTTCTCTCGTT; U6-qPCR-F: CTCGCTTCGGCAGCACA; and U6-qPCR-R: AACGCTTCACGAATTTGCGT.

### shRNAs, sgRNAs, and ISD90.

shRNAs were constructed by inserting synthesized oligos into pLKO-puro or pLKO-hygro vector. Primers are as follows: shScr: AACAGTCGCGTTTGCGACTGG; shSPOP-A2: CACAGATCAAGGTAGTGAAAT; shSPOP-A3: CAAGGTAGTGAAATTCTCCTA; shSPOP-C4: CAAACGCCTGAAGCAATCCTA; shSPOP-C6: CTCCTACATGTGGACCATCAA; shmSPOP-3: TGTGGACCATCAATAACTTTA; shmSPOP-4: GGAGAGTCAGCGAGCTTATAG; shmSPOP-6: CGCTTGAAGCAATCCTAAGAT; shSTING-29: GCAGAGCTATTTCCTTCCACA; shSTING-45: GTCCAGGACTTGACATCTTAA; shCSNK1G1-1: TGACCGAACATTTACTTTGAA; shCSNK1G1-2: GATGGCAACCTACCTTCGATA; shCSNK1G1-3: GAACCTCATTTACCGAGATGT; shCUL3-1: TTCAGGCTTTACAACGTTTAT; shCUL3-2: CGTGTGCCAAATGGTTTGAAA; shCBX4-1: GCCCTTCTTTGGGAATATAAT; and shCBX4-2: GCCTCAGAGTTCTAGTATTAT.

sgRNAs were constructed by inserting synthesized oligos into lentiCRISPRv2-puro vector. Primers are as follows: sgSPOP-1: CCTCTGCAGTAACCTGTCCG; sgSPOP-4: TGTCCAAAGAGTGAAGTTC; sgSPOP-11: CCAGTAACAGGTAAAGTGAC; sgSPOP-12: TGTTTGCGAGTAAACCCCAA; sgmSPOP-1: TTCGTGCAAGGCAAAGACTG; sgSTING-1B: GCTGGGACTGCTGTTAAACG; sgmSTING-2: TGCCTCAGATGAGGTCAGTG; sgmSTING-3: TCTTCAGAGCTTGACTCCAG; sgcGAS: GGCCGCCCGTCCGCGCAACT; and ISD90: TACAGATCTACTAGTGATCTATGACTGATCTGTACATGATCTACATACAGATCTACTAGTGATCTATGACTGATCTGTACATGATCTACA.

### Immunoblots and immunoprecipitations.

Cells were lysed in EBC buffer (50 mM Tris, pH 7.5, 120 mM NaCl, and 0.5% NP-40) or RIPA buffer (50 mM Tris, pH 7.5, 150 mM NaCl, 1% Triton X-100, 1% sodium deoxycholate, and 0.1% SDS) supplemented with protease and phosphatase inhibitors (K1008 and K1015, Apexbio). Protein concentrations were measured using Bio-Rad protein assay reagent on a NanoDrop OneC (Thermo Fisher Scientific). Equal amounts of lysates were resolved by SDS-PAGE and immunoblotted with indicated antibodies. For immunoprecipitation, 1 mg of lysate was incubated with the indicated antibody (1–2 μg) for 3–4 h at 4°C, followed by 1 h with 10 μL Protein A/G XPure Agarose Resin (P5030-5, UBPBio). Lysates with tagged proteins were incubated with tag-specific agarose-conjugated antibodies. For endogenous IPs, antibody incubation was performed overnight. Immunocomplexes were washed 5 times with NETN buffer (20 mM Tris, pH 8.0, 100 mM NaCl, 1 mM EDTA, and 0.5% NP-40) before SDS-PAGE and immunoblotting. Antibodies used for IB, IP, immunofluorescence, and FACS are listed in Supplemental Table 1.

### In-cell ubiquitination assays.

The 293T cells were transfected with His–Ub-WT or -K48 only and other indicated constructs and treated with 10 μM MG132 (S2619, Selleck) overnight. Cells were lysed in buffer A (6 M guanidine-HCl, 0.1 M Na_2_HPO_4_/NaH_2_PO_4_, and 10 mM imidazole, pH 8.0) and sonicated. Supernatants were incubated with nickel-nitrilotriacetic acid (Ni-NTA) resin for 3 h at room temperature. Ni-NTA pull-down products were washed twice with buffer A, twice with buffer A/TI (25% buffer A and 75% buffer TI), and once with buffer TI (25 mM Tris-HCl and 20 mM imidazole, pH 6.8). Products were resolved by SDS-PAGE and immunoblotted with indicated antibodies.

### Colony formation assays.

Cells (500/well) were seeded in 6- or 24-well plates and cultured at 37°C with 5% CO_2_ for 7–15 days until visible colonies formed. Colonies were washed with PBS, fixed in methanol for 30 min, and stained with 0.5% crystal violet for 30 min, followed by washing and air-drying. Colony numbers were manually counted, and data represent 3 independent experiments.

### RNA extraction and RT-qPCR.

RNA was extracted using the RNA Miniprep Super Kit (BS584, BioBasic), and concentration and purity were assessed using the NanoDrop OneC. cDNA was synthesized using the iScript kit (170-8891, Bio-Rad), and RT-qPCR was performed with iTaq SYBR Green Supermix (172-5124, Bio-Rad) on a QuantStudio 6 Flex system (Thermo Fisher Scientific). RT² Profiler PCR Arrays for mouse (PAMM-016Z) and human (PAHS-016Z) Type I Interferon Response (Qiagen) were used for RNA profiling. mRNA levels were normalized to GAPDH or U6 snRNA, and relative expression was calculated by the comparative Ct method.

### Cytosolic DNA isolation and qPCR.

B16-OVA cells were treated with or without 10 μM 6lc for 24 h. Genomic DNA was extracted from half of the cells using QuickExtract DNA Extraction Solution (QE09050, Bioresearch Technologies). Mitochondria-free cytosolic fractions were isolated from the remaining cells using a mitochondria isolation kit (89874, Thermo Fisher Scientific) per the manufacturer’s instructions. Briefly, cell pellets were sequentially treated with reagents A, B, and C, and cytosolic fractions were obtained by centrifugation at 12,000*g* for 15 min. DNA from whole cells and cytosolic fractions was purified using the DNA Clean & Concentrator-5 (D4013, Zymo Research) and quantified using the NanoDrop OneC. Levels of nuclear and mitochondrial genes in whole-cell DNA were normalized to DNA concentration, and cytosolic DNA levels were further normalized to whole-cell DNA.

### Generation of murine CAR T cells.

Murine T cells were isolated from splenocytes obtained from C57BL/6J mice and stimulated on plates coated with 1 mg/mL of mCD3 and 1 mg/mL of mCD28 mAbs, in complete RPMI 1640 for 48 h. Activated murine T cells were transduced with retroviral supernatants using retronectin-coated plates (Takara Bio) with the same protocol used to transduce human T cells with human IL-7/15 (10 ng/mL), as previously described (88). After removal from retronectin plates, T cells were expanded in complete RPMI 1640 medium with human IL-7/15 (10 ng/mL), changing medium every 2 days. On days 7–9, cells were collected and used for functional assays in vivo.

### Mouse xenograft assays.

B16 cells were transduced with lentiviruses expressing shScr, shmSPOP-6, shmSPOP-3, or shmSPOP-3+HA-SPOP. Two days later, 5-week-old female nude or C57BL/6J mice (The Jackson Laboratory; *n* = 5 per group, 10 injections total) were subcutaneously inoculated with 1 × 10^5^ B16 cells. Tumor dimensions were measured using calipers, and volumes were calculated as V = L × W² × 0.5. Mice were euthanized when the largest tumor reached 2,000 mm³, and tumors were excised and weighed.

For combination therapy studies, 1 × 10^5^ B16 cells (parental, sgCtrl, or sgSTING) were injected subcutaneously into the right flank of 5-week-old female C57BL/6J mice. When tumors became palpable (~day 7), mice were randomized into 4 treatment groups. Compound 6b (8 mg/mL in 10% DMSO, 40% PEG300, 5% Tween 80, and 45% saline) was administered intraperitoneally at 60 mg/kg daily, 6lc at 20 mg/kg daily, and anti–PD-1 antibody (BE0273, BioXCell) at 250 μg intraperitoneally every 3 days. Tumor growth was monitored as above, and tumors were collected at endpoint for flow cytometry analysis of infiltrating immune cells.

### Comet assay.

Single-strand DNA breaks were assessed using a Comet assay as previously described (44). B16 cells were treated with 10 μM 6b for 24 h. Low-gelling agarose (0.5% and 1.5%; A4018, Sigma) was prepared, and slides were precoated with 1.5% agarose. Cells (10^4^ per slide) were mixed with 0.5% agarose, layered onto precoated slides, and gelled at 4°C for 2 min. Slides were lysed overnight at 4°C in lysis solution (2.5 M NaCl, 100 mM EDTA, 10 mM Tris-HCl, pH 7.5, 200 mM NaOH, 1% Triton X-100, and 10% DMSO) in the dark and then equilibrated in electrophoresis solution (300 mM NaOH and mM EDTA, pH 13) and subjected to electrophoresis at 25 V and 300 mA for 25 min. Slides were neutralized with 0.4 M Tris-HCl (pH 8.0), stained with propidium iodide (10 μg/mL), and washed with water. At least 50 comet images per condition were captured at ×20 magnification (Olympus IX51). Tail moment was quantified as follows: tail length × tail intensity/comet intensity.

### K-ε-GG peptide enrichment and LC-MS/MS.

A2058 cells stably expressing shScramble or shSPOP-C4 were treated with or without 10 μM 6lc for 12 h (*n* = 3 per group). Cells were washed with PBS, lysed in heated 5% SDS/50 mM triethylammonium bicarbonate buffer (pH 7.55) with 5 mM Tris(2-carboxyethyl)phosphine at 95°C, sonicated, and alkylated with 15 mM methyl methanethiosulfonate for 30 min. Proteins were quantified using the Bio-Rad assay and digested using S-Trap Midi columns (UNC Metabolomics and Proteomics Core). Peptides were quantified with the Pierce fluorometric assay; 820 μg per sample was processed, and a pooled aliquot was used for quality control. Approximately 800 μg per sample underwent K-ε-GG enrichment using the PTMScan HS Ubiquitin Remnant Motif Kit (59322, Cell Signaling Technology); 2% input was reserved for proteome analysis. Samples were desalted and analyzed by LC-MS/MS (Ultimate3000-Exploris480; proteome: 130 min DIA; K-ε-GG: 100 min DIA). Data were analyzed in Spectronaut (v17.1) using the UniProt Human (reviewed in January 2023) and MaxQuant contaminant databases. Single-peptide identifications were excluded from proteome data; imputation and cross-run normalization were applied. For K-ε-GG data, digly-Lys was set as a variable modification, cross-run normalization was enabled, and no imputation was performed. Statistical analyses were conducted in Spectronaut.

### Flow cytometry.

To analyze tumor-infiltrating immune cells, B16 tumors were digested using a tumor dissociation kit, mouse (130-096-730, Miltenyi Biotec) and gentleMACS Dissociator (Miltenyi Biotec) according to the manufacturer’s protocol. Single-cell suspension was incubated with corresponding fluorophore-conjugated antibodies and isotype controls. Samples were acquired on a Symphony A3 or Fortessa flow cytometer from BD Biosciences. Data were analyzed using FlowJo 10.8.1.

### scRNA-seq analysis.

The B16-bearing mice were harvested at day 14. The scRNA-seq was done as previously described (61). In brief, tumor-infiltrating CD45^+^ cells were enriched through positive selection via anti-CD45 biotinylated antibody–and streptavidin-labeled microbeads and Miltenyi MACS LS columns. Then PE-CD45^+^ cells were sorted on a Sony XYZ instrument, and 10,000 cells were loaded in 10x Genomics Chromium Single Cell 3′ inlets (1 inlet per sample). Barcoding and library preparation were performed following the manufacturer’s instructions with the 10x Genomics Chromium GEM-X Single Cell 3′ kit (v4). Sequencing was performed on an Illumina NextSeq 2000 at the UNC High-Throughput Sequencing Facility. Sequencing reads were mapped to mm10, and unique molecular identifier counts were collapsed based on the 10x Genomics Cell Ranger software (version 8.0.1). Resulting datasets were analyzed via the Seurat package (v5.1.0) in R (v4.3.1). Raw counts were processed following standard quality control measures, and low-quality cells were excluded, including dead and suspected doublets. The minimum number of principal components needed to represent the data was calculated using a JackStraw plot, and clustering was performed at a resolution of 0.7.

### Immunofluorescence.

Cells plated onto glass coverslips were fixed with 4% paraformaldehyde in PBS for 20 min at room temperature and permeabilized with 0.2% Triton X-100 for 20 min at room temperature. Cells were incubated with blocking buffer (5% BSA and 0.1% Triton X-100 in PBS) for 1 h, incubated with primary antibodies at 4°C overnight, incubated with secondary antibodies at room temperature for 1 h, and mounted with ProLong Gold antifade reagent (P36931, Invitrogen). Fluorescent signals were observed with an Olympus FV1000 confocal microscope at ×60 or ×100 magnification.

### IHC analysis.

Freshly isolated B16 tumors from C57BL/6 mice were fixed in 10% neutral-buffered formalin for 48 h, transferred to cassettes, stored in 70% ethanol, embedded in paraffin, and sectioned into 5 consecutive 6 μm slices. For IHC, slides were deparaffinized in xylene (2 × 10 min), rehydrated through graded ethanol (100%, 95%, 85%, 70%), and rinsed in TBST (15 min) followed by TBS (5 min). Endogenous peroxidase activity was quenched with 1% hydrogen peroxide in methanol (10 min). Antigen retrieval was performed by microwaving slides in 0.01 M sodium citrate buffer (pH 6.0, 0.05% Tween 20) for 5 min at full power and 10 min at 50% power, then cooling for 30 min. After TBS washes (3 × 3 min), sections were blocked in buffer (10 mM Tris-HCl, 0.1 M MgCl_2_, 0.5% Tween 20, 1% BSA, and 10% goat serum) for 1 h at room temperature. Primary antibodies diluted in 2% BSA/PBS were applied overnight at 4°C. Slides were washed and incubated with a biotinylated secondary antibody (1:400; Vector Labs) for 1 h and then with avidin-biotin complex (ABC reagent, Vector Labs) for 45 min. Chromogenic detection was performed with freshly prepared DAB substrate (Vector Labs) for optimized times (CD3ε, 3 min; CD8α, 5 min; FoxP3, 3 min; STING, 1 min; PD-L1, 4 min). Reactions were stopped in running tap water. Slides were counterstained with diluted Harris hematoxylin (2 min), dehydrated through graded ethanols and xylene, and mounted with Permount (Electron Microscopy Sciences).

### Protein purification.

His-CBX4 and His-SUMO-avi-SPOP (28–359 aa) were expressed in *E*. *coli* BL21 (DE3) CodonPlus-RIL cells grown in Luria broth with kanamycin (50 μg/mL) (CBX4), ampicillin (150 μg/mL) (SPOP), and chloramphenicol (30 μg/mL) at 37°C to OD_600_ = 0.8, followed by induction with 0.6 mM IPTG at 16°C for 18 h. Cells were lysed in buffer (50 mM HEPES, pH 7.5, 200 mM NaCl, 20 mM imidazole, pH 8.0, 5 mM BME, and 0.001% PMSF) by sonication, and lysates were clarified at 23,916*g* for 45 min. Proteins were purified using Ni-NTA resin (R-202-100, GoldBio) and dialyzed (3.5 kDa cutoff) overnight (50 mM HEPES, pH 7.5, 200 mM NaCl, and 2 mM DTT). His-CBX4 was stored after dialysis. Avi-SPOP was cleaved from SUMO using ULP1 (1:50) during dialysis, further purified by size-exclusion chromatography (20 mM HEPES, pH 8.0, 200 mM NaCl, and 1 mM DTT), and biotinylated with biotin maleimide.

### Streptavidin pulldown.

Dynabeads MyOne Streptavidin T1 (10 μL; 65602, Thermo Fisher Scientific) were washed twice with NETN buffer before use. Beads were incubated with 1 μM biotin-SPOP or D-(+)-biotin (ALX-460-002-G001, Enzo Life Sciences) in 100 μL buffer for 1 h and washed once with NETN to remove unbound biotin. The beads were then incubated with the indicated concentrations of SPOP inhibitors for 30 min, followed by incubation with 0.5 μM CBX4 for 1 h. After 4 washes with NETN buffer, bound proteins were eluted, separated by SDS-PAGE, and immunoblotted with the indicated antibodies.

### Statistics.

Statistical analyses were performed using GraphPad Prism 8. Two-group comparisons were conducted using 2-tailed unpaired Student’s *t* tests. For 3 or more groups, normally distributed data were analyzed by 1- or 2-way ANOVA with Dunnett’s, Tukey’s, Fisher’s least significant difference (LSD), or Bonferroni’s post hoc tests as appropriate; nonnormally distributed data were analyzed using Kruskal-Wallis with Dunn’s test. Data are shown as mean ± SD from representative experiments repeated at least twice or as mean ± SEM from at least 2 independent experiments or biological replicates. *P* values of less than 0.05 were considered significant.

### Study approval.

All mouse studies were reviewed and approved by the UNC IACUC (22-056, 23-192, and 25-017.0). Experiments were conducted in the Genetic Medicine Animal Facility at UNC-Chapel Hill, an Office of Laboratory Animal Welfare–assured and AAALAC-accredited facility, following IACUC-approved protocols and in compliance with the Guide for the Care and Use of Laboratory Animals (National Research Council, 2011).

### Data availability.

All reported data values are available in the Supporting Data Values file. scRNA-seq data supporting the findings in this study have been deposited in the Gene Expression Omnibus (GSE280269). All other data supporting the findings in this study are available from the corresponding authors upon reasonable request.

## Author contributions

Conceptualization, project administration, and supervision: GD and PL. Methodology: ZZ, XZ, GD, PL, MGW, and LEH. Investigation: ZZ, XZ, MX, JC, KCR, and ACM. Visualization: ZZ, XZ, GA, MGW, LEH, GD, and PL. Funding acquisition: PL. Writing, original draft: ZZ, XZ, and PL.

## Funding support

This work is the result of NIH funding, in whole or in part, and is subject to the NIH Public Access Policy. Through acceptance of this federal funding, the NIH has been given a right to make the work publicly available in PubMed Central.

- NIH grant R01CA244825 (to PL).
- Department of Defense, Congressionally Directed Medical Research Program, Kidney Cancer Research Program, Idea Development Award HT9425-24-1-0644 (to PL).
- The University of North Carolina at Chapel Hill University Cancer Research Fund (to PL).
- National Cancer Institute (NCI) Center Core Support Grant CA16086.

## Supplementary Material

Supplemental data

Unedited blot and gel images

Supporting data values

## Version 1 10/28/2025

In-Press Preview

## Version 2 12/15/2025

Electronic publication
