# Supplementary material for: Molecular glue degrader function of SPOP inhibitors enhances STING-dependent immunotherapy efficacy in melanoma models
Source: J Clin Invest. 2025 Oct 28;135(24):e191772. doi: 10.1172/JCI191772 (PMC12700557; doi:10.1172/JCI191772)
Supplement: Supplemental data [file jci-135-191772-s247.pdf]

Supplemental information

Molecular glue degrader function of SPOP inhibitors enhances STING-dependent immunotherapy efficacy in melanoma models

Zhichuan Zhu, Xin Zhou, Max Xu, Jianfeng Chen, Kevin C. Robertson, Gatphan Atassi, Mark G. Woodcock, Allie C. Mills, Laura E. Herring, Gianpietro Dotti, and Pengda Liu

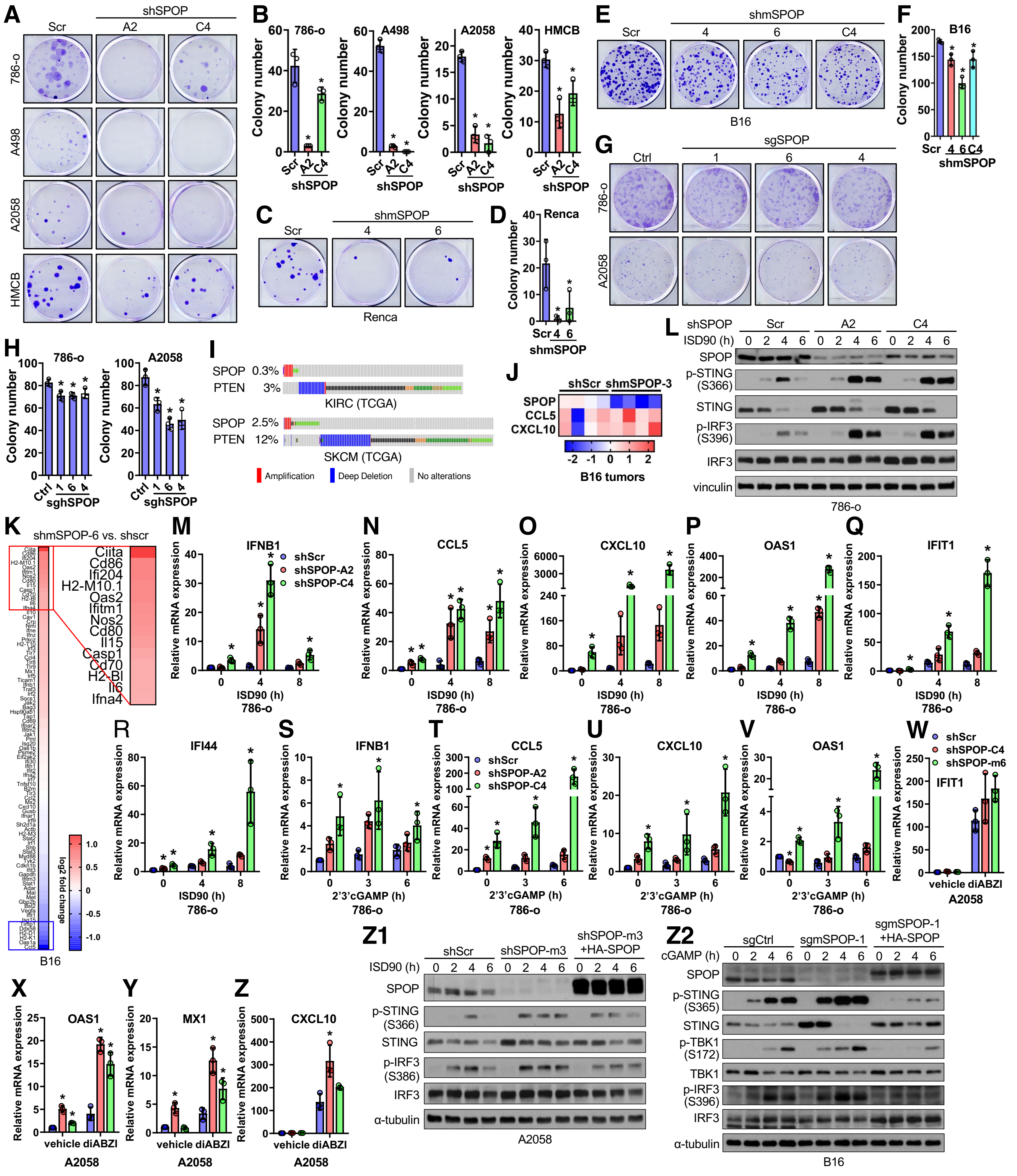

Fig. S1. SPOP depletion facilitates DNA-induced cGAS/STING activation. (**A**, **C**, **E** and **G**) Representative images for 2D colony formation assays using indicated cells with or without SPOP depletion. (**B**, **D, F** and **H**) Quantification of A, C, E and G, respectively. Error bars represent SD, n = 3. *p < 0.05 compared with shScr or sgCtrl. (**I**) Oncoprint showing genetic alteration of SPOP and PTEN in KIRC and SKCM patients from the TCGA PanCancer Atlas database. (**J**) RT-PCR analysis of indicated gene expression from indicated harvested xenografted B16 tumors from C57BL/6 mice. (K) RNA

expression profiling heatmap of genes in human type-I interferon response in SPOP depleted B16 cells. (**L**) IB analyses of control and SPOP depleted 786-o cells transfected with 5 μg/mL of ISD90 for indicated periods. (**M** to **V**) RT-PCR analyses of control and SPOP depleted 786-o cells treated with 5 μg/mL of ISD90 (M to R) or 5 μg/mL of 2'3'cGAMP (S to V) for indicated periods. Error bars represent SD, n = 3. *$p < 0.05$ compared with shScr. (**W** to **Z**) RT-PCR analyses of control and SPOP depleted A2058 cells treated with 3 μM of diABZI for indicated periods. Error bars represent SD, n = 3. *$p < 0.05$ compared with shScr. (**Z1**) IB analyses of indicated A2058 cells transfected with 5 μg/mL of ISD90 for indicated periods. (**Z2**) IB analyses of indicated B16 cells treated with 5 μg/mL of 2'3'cGAMP for indicated periods. All p values were calculated using one-way ANOVA followed by Dunnett's multiple comparison test. Representative immunoblots shown in figures were repeated at least two times independently with similar results.

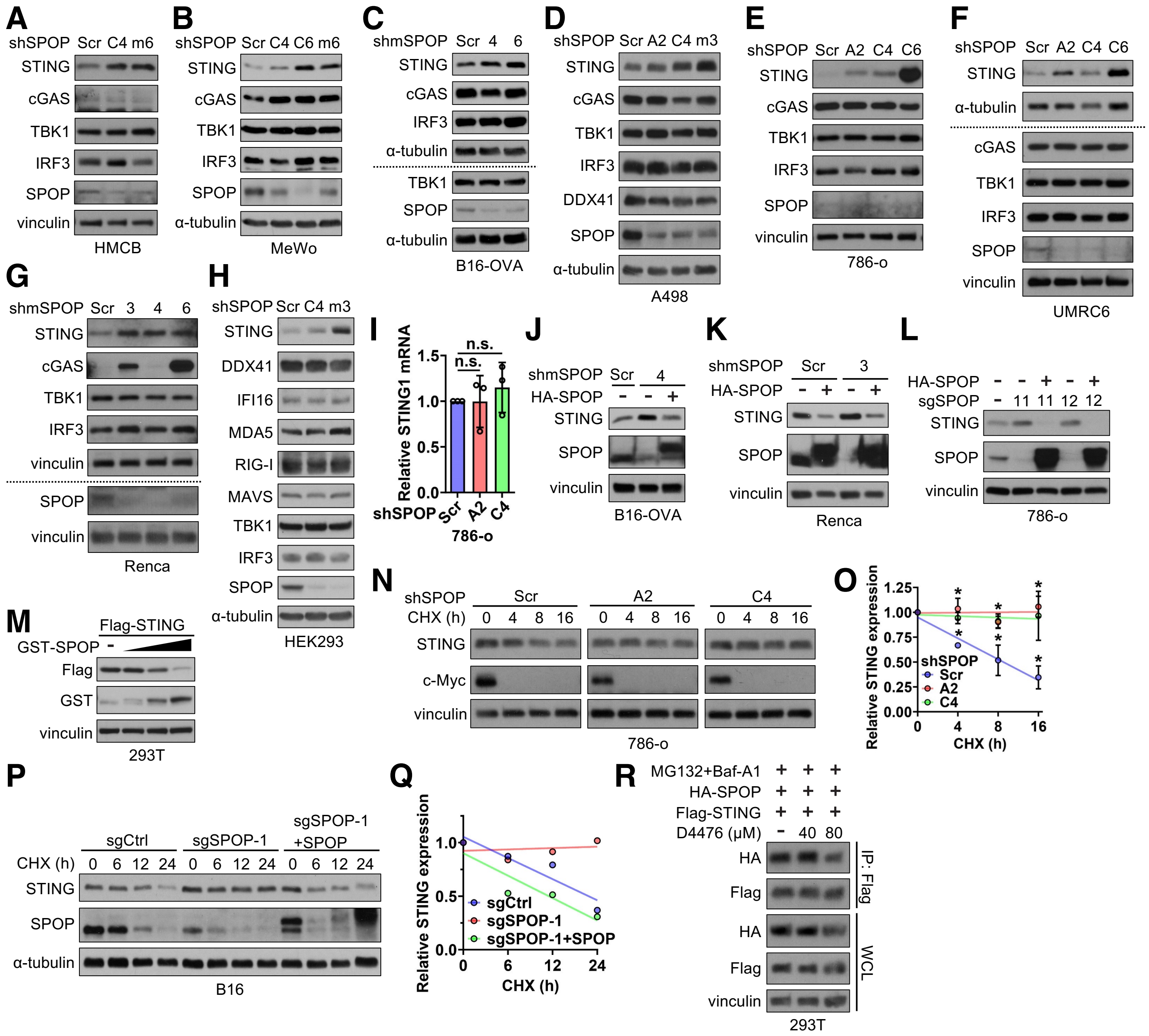

Fig. S2. SPOP targets STING for ubiquitination and degradation. (**A** to **H**) IB analyses of indicated cells depleted of SPOP. (**I**) RT-PCR analyses of mRNA changes in control and SPOP depleted 786-o cells. Error bars represent SD, n = 3. n.s. compared with shScr (one-way ANOVA followed by Dunnett's multiple comparison test). (**J** to **L**) IB analyses of indicated cells with SPOP depletion and rescue. (**M**) (**H**) IB analyses of 293T cells transfected with fixed dose of STING construct and increased dose of SPOP construct. (**N**) IB analysis of control and SPOP depleted 786-o cells treated with 100 μg/mL of cycloheximide (CHX) for indicated periods. (**O**) Quantification of relative STING grayscales in (N). Error bars represent SEM, n = 3. *p < 0.05 compared with shScr (two-tailed unpaired Student's t test). (**P**) IB analysis of control and SPOP depleted and re-expressed B16 cells treated with 100 μg/mL of cycloheximide (CHX) for indicated periods. (**Q**) Quantification of relative STING grayscales in (P). (**R**) IB analysis of WCL derived from 293T cells transfected with indicated constructs and then treated with indicated dose of D4476, 10 μM of MG132 and 20 nM of Baf-A1 for 6 hrs. Representative immunoblots shown in figures were repeated at least two times independently with similar results.

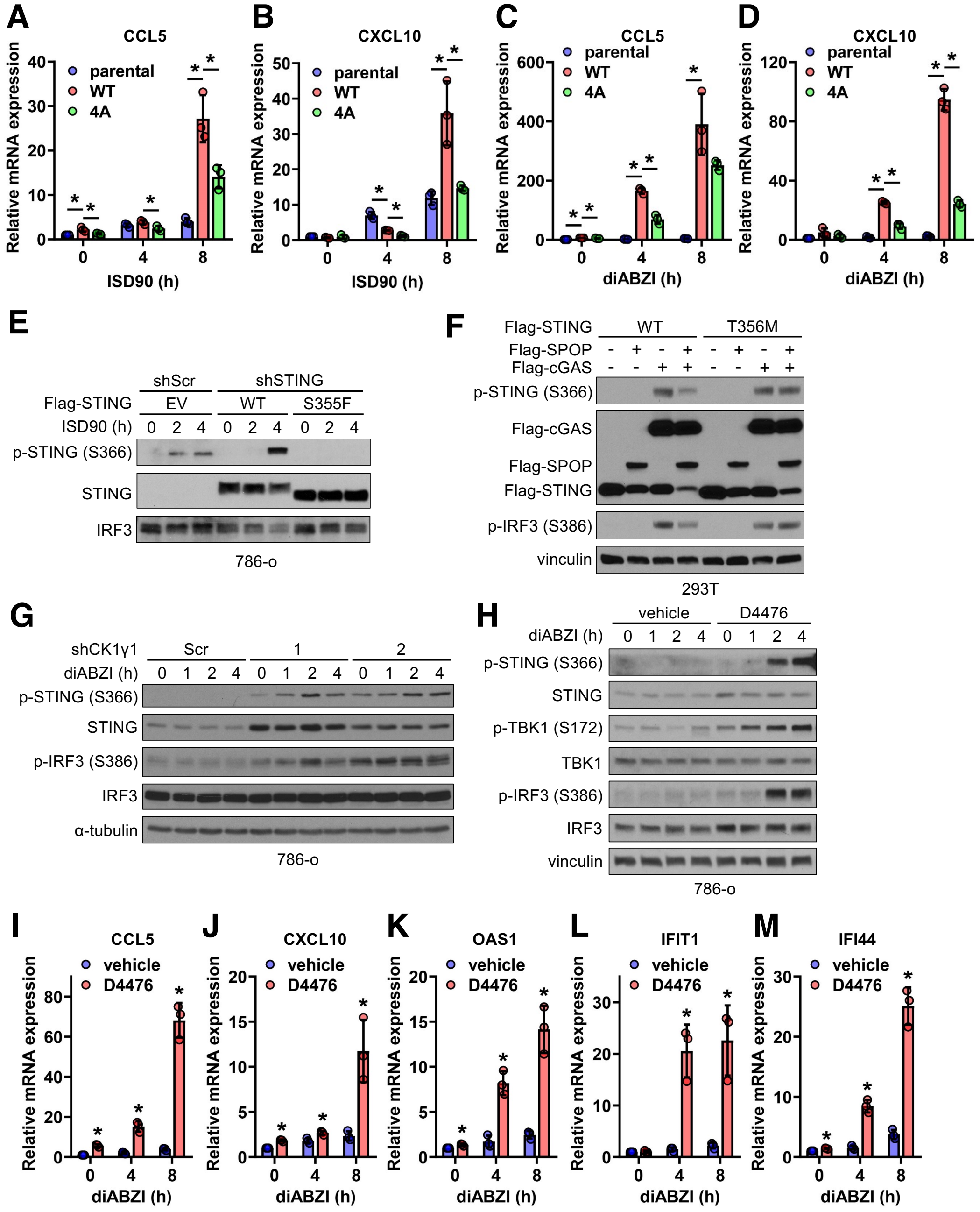

Fig. S3. CK1γ phosphorylates and primes STING for SPOP recognition. (**A** to **D**) RT-PCR analyses of indicated 786-o stable cell lines treated with 5 μg/ml of ISD90 (A and B) or 3 μM of diABZI (C and D) for indicated periods. Error bars represent SD, n = 3. (**E**) IB analyses of indicated 786-o cells treated with 5 μg/ml of ISD90 for indicated periods. (**F**) IB analyses of 293T cells transfected with indicated constructs. (**G**) IB analyses of control and CK1γ1 depleted 786-o cells treated with 3 μM of diABZI for indicated periods. (**H** to **M**) IB analyses (H) and RT-PCR analyses (I to M) of 786-o cells treated first with 40 μM of D4476 for 24 hrs and then with 3 μM of diABZI for indicated periods. Error bars represent SD, n = 3. *P* values were calculated using one-way ANOVA followed by Tukey's multiple comparison test (A-D) or two-tailed unpaired Student's t test (I-M). *p < 0.05. Representative immunoblots shown in figures were repeated at least two times independently with similar results.

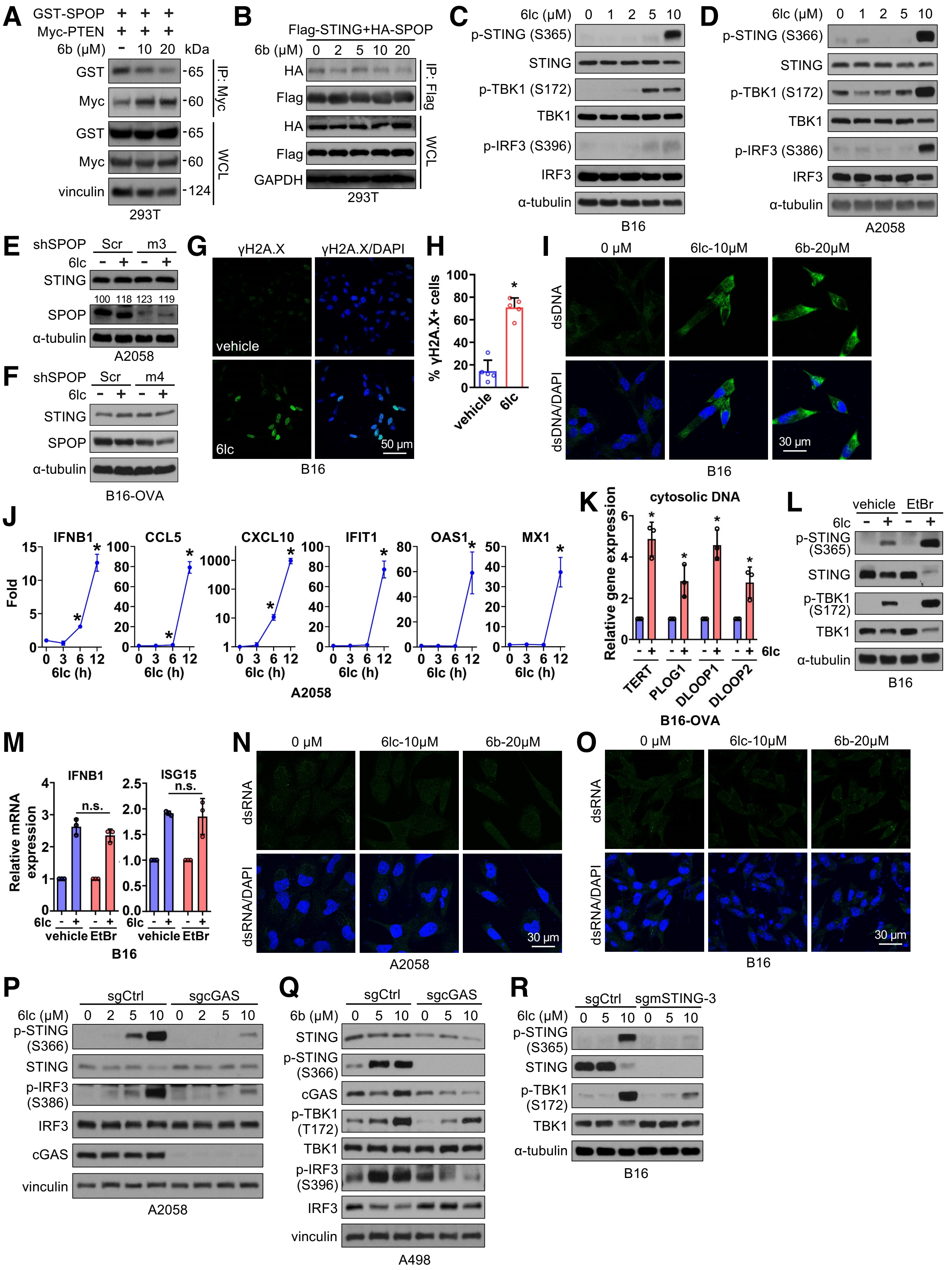
A
GST-SPOP
Myc-PTEN
6b (μM) - 10 20 kDa
GST -65
Myc -60
GST -65
Myc -60
vinculin -124
IP: Myc
WCL
293T
B
Flag-STING+HA-SPOP
6b (μM) 0 2 5 10 20
HA
Flag
HA
Flag
GAPDH
IP: Flag
WCL
293T
C
6lc (μM) 0 1 2 5 10
p-STING (S365)
STING
p-TBK1 (S172)
TBK1
p-IRF3 (S396)
IRF3
α-tubulin
B16
D
6lc (μM) 0 1 2 5 10
p-STING (S366)
STING
p-TBK1 (S172)
TBK1
p-IRF3 (S386)
IRF3
α-tubulin
A2058
E
shSPOP Scr m3
6lc - + - +
STING
100 118 123 119
SPOP
α-tubulin
A2058
F
shSPOP Scr m4
6lc - + - +
STING
SPOP
α-tubulin
B16-OVA
G
γH2A.X γH2A.X/DAPI
vehicle
6lc
50 μm
B16
H
% γH2A.X+ cells
vehicle 6lc
I
0 μM 6lc-10μM 6b-20μM
dsDNA
dsDNA/DAPI
30 μm
B16
J
IFNB1 CCL5 CXCL10 IFIT1 OAS1 MX1
Fold
6lc (h)
A2058
K
cytosolic DNA
Relative gene expression
TERT PLOG1 DLOOP1 DLOOP2
6lc
B16-OVA
L
vehicle EtBr
6lc - + - +
p-STING (S365)
STING
p-TBK1 (S172)
TBK1
α-tubulin
B16
M
IFNB1 ISG15
n.s.
Relative mRNA expression
6lc
vehicle EtBr
B16
N
0 μM 6lc-10μM 6b-20μM
dsRNA
dsRNA/DAPI
30 μm
A2058
O
0 μM 6lc-10μM 6b-20μM
dsRNA
dsRNA/DAPI
30 μm
B16
P
sgCtrl sgcGAS
6lc (μM) 0 2 5 10 0 2 5 10
p-STING (S366)
STING
p-IRF3 (S386)
IRF3
cGAS
vinculin
A2058
Q
sgCtrl sgcGAS
6b (μM) 0 5 10 0 5 10
STING
p-STING (S366)
cGAS
p-TBK1 (T172)
TBK1
p-IRF3 (S396)
IRF3
vinculin
A498
R
sgCtrl sgmSTING-3
6lc (μM) 0 5 10 0 5 10
p-STING (S365)
STING
p-TBK1 (S172)
TBK1
α-tubulin
B16

Fig. S4. SPOP inhibitor 6b or 6lc induces STING activation. (**A** and **B**) IB analysis of Myc-IP (A) or Flag-IP (B) and WCL derived from 293T cells transfected with indicated constructs and then treated with indicated dose of 6b and 10 μM of MG132 for 12 hrs. (**C** and **D**) IB analysis of indicated cells treated with indicated dose of 6lc for 24 hrs. (**E** and **F**) IB analysis of control and SPOP depleted cells treated with 10 μM of 6lc for 24 hrs. (**G** and **H**) Immunofluorescence of γH2AX in B16 cells treated with 10 μM of 6lc for 24 hrs and quantification of percentages of γH2AX positive cells. Error bars represent SD, n = 5. (**I**) Immunofluorescence of dsDNA in B16 cells treated with indicated doses of 6lc or 6b for 12 hrs. (**J**) RT-PCR analyses of expression of indicated genes from A2058 cells treated with 10 μM of 6lc for indicated periods. Error bars represent SD, n = 3. (**K**) RT-PCR analyses of expression of genomic DNA (TERT and PLOG1) and mitochondrial DNA (DLOOP1 and DLOOP2) in the cytosolic fraction of B16-OVA cells treated with 10 μM of 6lc for 24 hrs. Error bars represent SD, n = 3. (**L** and **M**) IB analyses (L) and RT-PCR (M) of B16 cells pretreated with 250 nM of EtBr for 24 hrs and treated with 20 μM of 6lc for 12 hrs. Error bars represent SD, n = 3. (**N** and **O**) Immunofluorescence of dsRNA in A2058 (N) or B16 (O) cells treated with indicated doses of 6lc or 6b for 12 hrs. (**P** and **Q**) IB analyses of indicated control and cGAS-depleted cells treated with indicated dose of 6lc or 6b for 24 hrs. (**R**) IB analyses of control and STING-depleted B16 cells treated with indicated dose of 6lc for 24 hrs. *P* values were calculated using two-tailed unpaired Student's t test (H, J and K) or one-way ANOVA followed by Tukey's multiple comparison test (M). *p < 0.05. Representative immunoblots shown in figures were repeated at least two times independently with similar results.

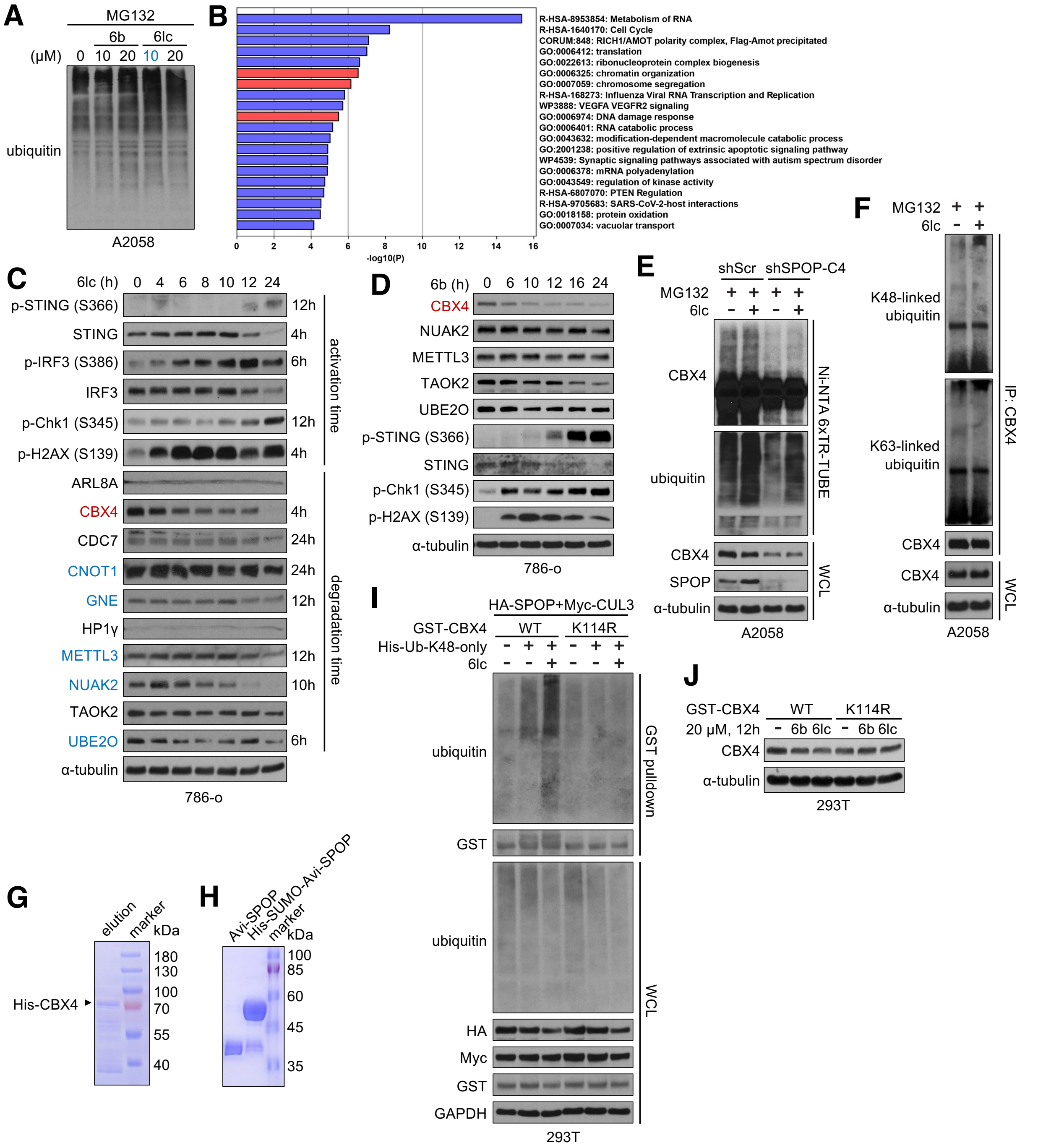

Fig. S5. SPOP inhibitor 6b or 6lc functions as a molecular glue to engage neo-substrates for SPOP-dependent degradation. (**A**) IB analysis of A2058 treated with indicated dose of 6b and 6lc and co-treated with 10 μM of MG132 for 12 hrs. (**B**) Functional analyses of SPOP-dependent 6lc-induced ubiquitinated proteins by Metascape. (**C** and **D**) IB analysis of 786-o cells treated with 20 μM of 6lc or 6b for indicated periods. (**E**) IB analyses of WCL and 6×TR-TUBE-conjugating Ni-NTA pulldown product derived from control and SPOP depleted A2058 cells treated with 10 μM of 6lc and 10 μM of MG132 for 12 hrs. (**F**) IB analyses of CBX4-IP and WCL derived from A2058 cells treated as (E). (**G** and **H**) Coomassie blue staining of purified His-CBX4 (G) and purified Avi-SPOP before and after His-SUMO removal (H). (**I**) IB analyses of WCL and GST pulldown products derived from 293T cells transfected with indicated constructs and then treated with 20 μM of 6lc and 10 μM of MG132 for 6 hrs.

(**J**) IB analyses of 293T cells transfected with indicated constructs and then treated with 20 μM of 6b or 6lc for 12 hrs. Representative immunoblots shown in figures were repeated at least two times independently with similar results.

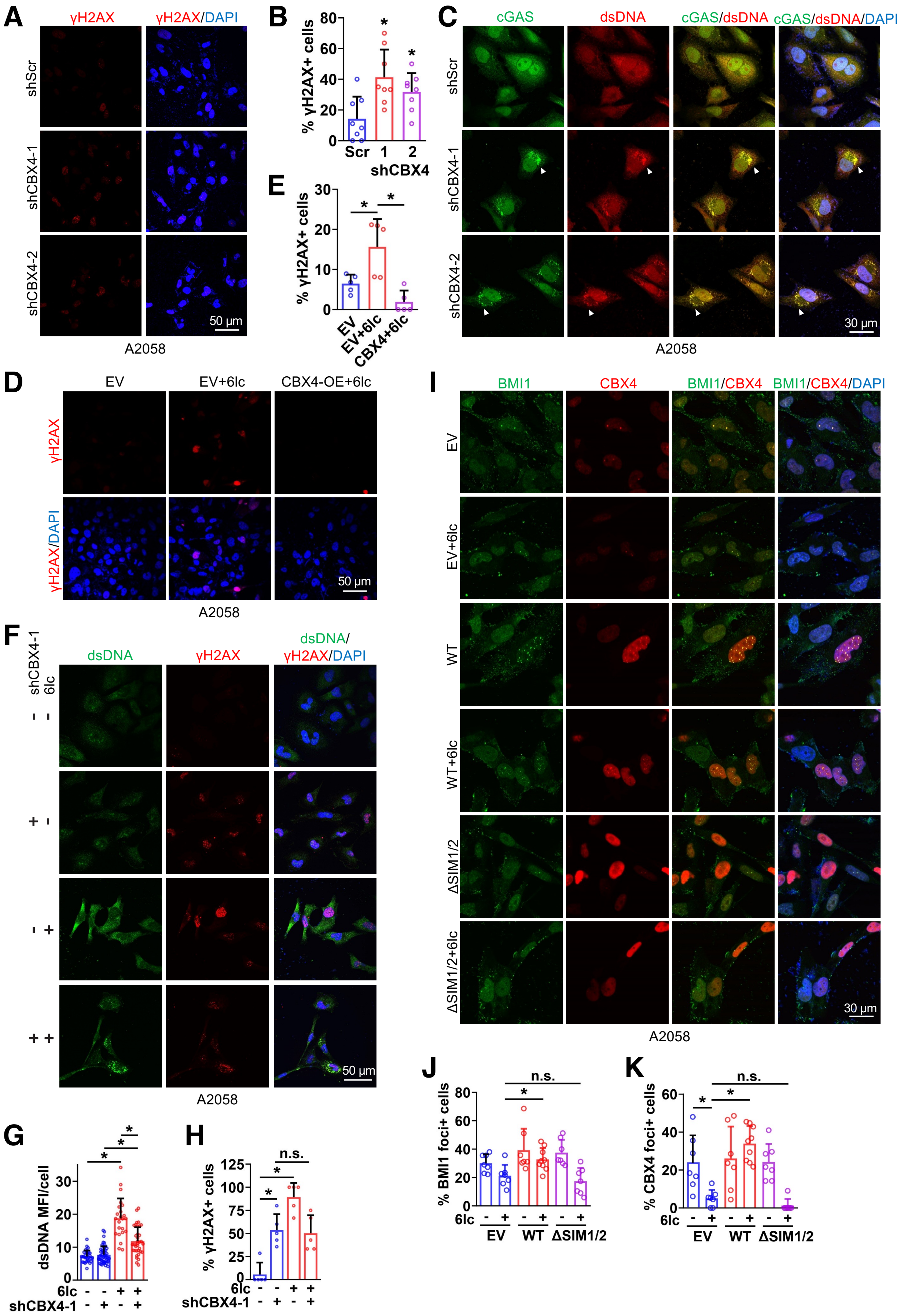

A
γH2AX
γH2AX/DAPI
shScr
shCBX4-1
shCBX4-2
50 μm
A2058
B
% γH2AX+ cells
Scr
1
2
shCBX4
C
cGAS
dsDNA
cGAS/dsDNA
cGAS/dsDNA/DAPI
30 μm
E
EV
EV+6lc
CBX4+6lc
D
EV
EV+6lc
CBX4-OE+6lc
γH2AX/DAPI
I
BMI1
CBX4
BMI1/CBX4
BMI1/CBX4/DAPI
WT
WT+6lc
ΔSIM1/2
ΔSIM1/2+6lc
F
shCBX4-1
6lc
dsDNA
dsDNA/γH2AX/DAPI
J
% BMI1 foci+ cells
n.s.
K
% CBX4 foci+ cells
G
dsDNA MFI/cell
H

Fig. S6. 6lc-induced DNA damage could be partially rescued by ectopic CBX4 expression. (**A** and **B**) Immunofluorescence of γH2AX in control and CBX4 depleted A2058 cells and quantification of percentages of γH2AX positive cells. Error bars represent SD, n = 8. (**C**) Immunofluorescence of dsDNA and cGAS in control and CBX4 depleted A2058 cells. (**D** and **E**) Immunofluorescence of γH2AX in control and CBX4-overexpressing A2058 cells treated with 10 μM 6lc for 24 hrs and quantification of percentages of γH2AX positive cells. Error bars represent SD, n = 5. (**F** to **H**) Immunofluorescence of dsDNA and γH2AX in indicated A2058 cells treated with 10 μM 6lc for 24 hrs and quantification of dsDNA MFI (n=23-48) or γH2AX positive cells (n=6-8). Error bars represent SD. (**I** to **K**) Immunofluorescence of BMI1 and CBX4 in control and CBX4-overexpressing A2058 cells treated with 10 μM 6lc for 24 hrs and quantification of percentages of BMI1 and CBX4 foci. Error bars represent SD, n = 7-9. *P* values were calculated using one-way ANOVA followed by Fisher's LSD test (B and E), Tukey's multiple comparison test (G and H) or Bonferroni multiple comparison test (J and K). *$p < 0.05$, n.s., non-significant.

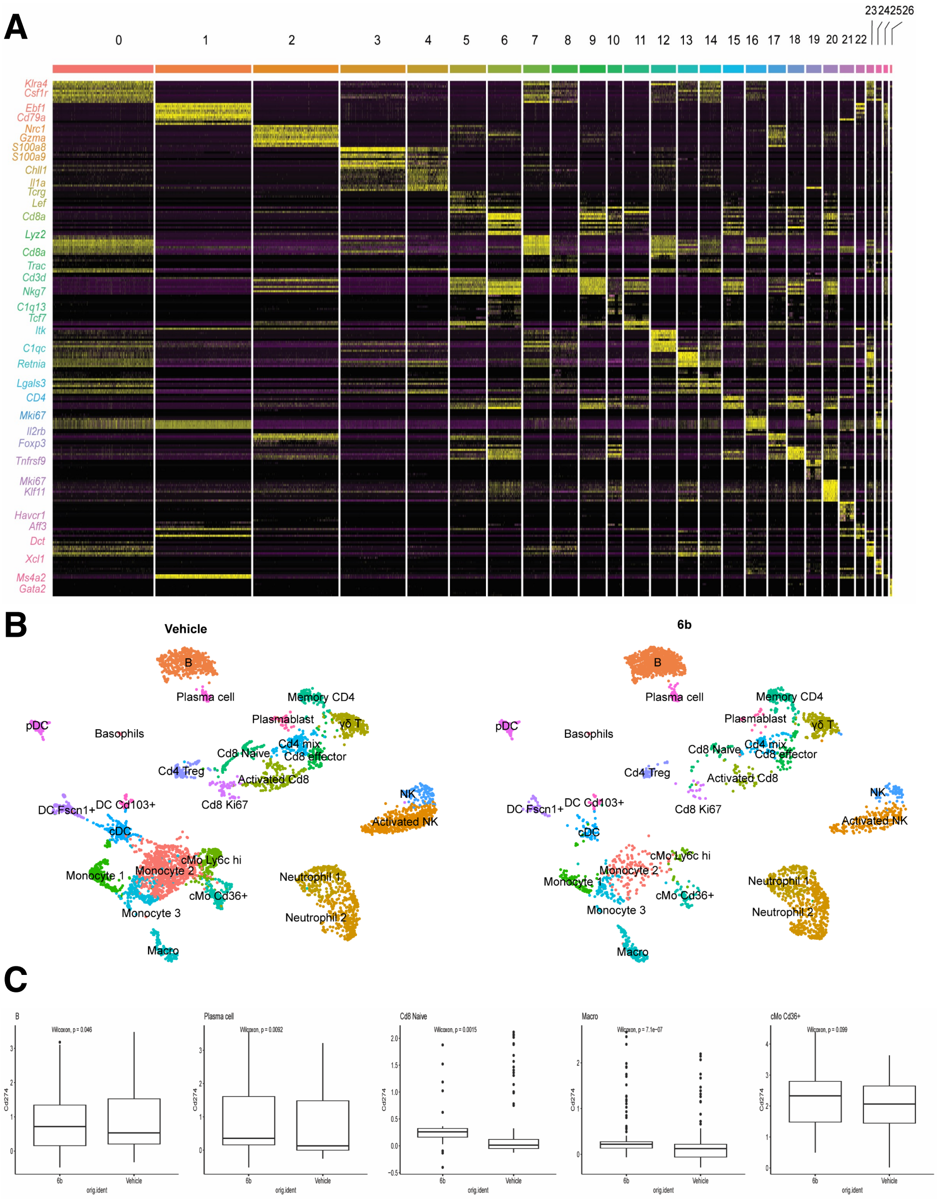

Fig. S7. Single-cell RNA seq analysis of B16 tumor immune cell population changes upon 6b treatment. (**A**) Differential gene expression analysis indicating upregulated genes in each cluster. The color of highlighted genes corresponds to cluster identity color. Key delineating genes or functional genes are highlighted. (**B**) UMAP plots represent the clustering of cells in single RNA sequence data from tumors collected from (a) each group. (**C**) PD-L1 expression analysis in cluster 1, 2, 15, 3, 18 from a single RNA sequence (Wilcoxon rank-sum test).

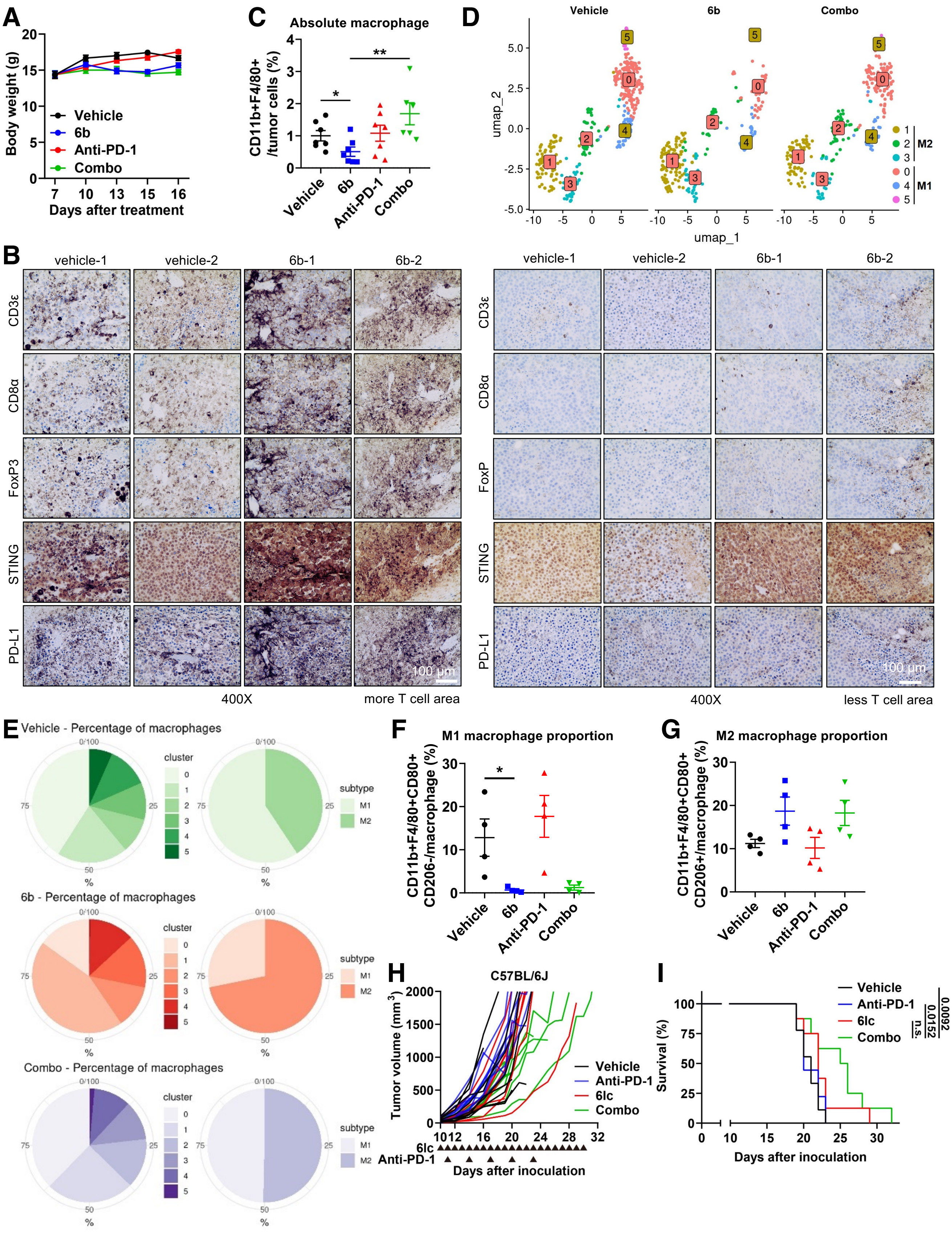
A
Body weight (g)
Days after treatment
Vehicle
6b
Anti-PD-1
Combo
C
Absolute macrophage
CD11b+F4/80+ /tumor cells (%)
Vehicle
6b
Anti-PD-1
Combo
D
Vehicle
6b
Combo
umap_2
umap_1
M2
M1
B
vehicle-1
vehicle-2
6b-1
6b-2
CD3ε
CD8α
FoxP3
STING
PD-L1
100 μm
400X
more T cell area
FoxP
less T cell area
E
Vehicle - Percentage of macrophages
6b - Percentage of macrophages
Combo - Percentage of macrophages
cluster
subtype
M1
M2
%
F
M1 macrophage proportion
CD11b+F4/80+CD80+ CD206-/macrophage (%)
G
M2 macrophage proportion
CD11b+F4/80+CD80+ CD206+/macrophage (%)
H
C57BL/6J
Tumor volume (mm3)
Vehicle
Anti-PD-1
6lc
Combo
6lc
Anti-PD-1
Days after inoculation
I
Survival (%)
Vehicle
Anti-PD-1
6lc
Combo
n.s.
0.0152
0.0092
Days after inoculation

Fig. S8. SPOP/6lc enhances immunotherapy effects in murine models. (**A**) Measurements of mouse body weights at indicated days after treatment of indicated agents. Error bars represent SEM. Vehicle, 6b and Anti-PD-1: n = 9; Combo: n = 8. p values are as indicated (two-way ANOVA followed by Tukey's multiple comparison test). (**B**) Representative IHC images for control of 6b treated B16 tumors stained with indicated antibodies. (**C**) The absolute percentages of macrophages in implanted B16 tumors from mice treated with indicated agents were analyzed by flow cytometry. Error bars represent SEM. Vehicle, 6b and Anti-PD-1: n = 7; Combo: n = 6. *$p < 0.05$, **$p < 0.01$, (one-way ANOVA followed by Tukey's multiple comparison test). (**D**) UMAP plots represent the clustering of cells (M1 or M2 as indicated) in scRNA-Seq data from tumors collected from indicated treatment group. (**E**) Pie charts from scRNA-Seq analysis revealing the percentage of tumor-infiltrating M1 and M2 macrophages from indicated treatment groups. (**F** and **G**) Percentage of M1 and M2 macrophages in macrophages in B16 tumors from mice treated with indicated agents were analyzed by flow cytometry. Error bars represent SEM, n = 4. *$p < 0.05$ (one-way ANOVA followed by Bonferroni multiple comparison test). (**H**) Individual B16-OVA tumor volume measurements at indicated days after treatment of indicated agents. Arrowheads indicate treatment schedule of indicated agents. Vehicle and Anti-PD-1: n = 9; 6lc and Combo: n = 8. (**I**) Survival curve of the B16 model in (H). Kaplan-Mayer analysis with log-rank (Mantel-Cox) test between each two groups.

Table S1. Antibodies

Antibody Name	Company	Catalog No.
CBX4	Cell Signaling Technology	30559
CD3ε	Cell Signaling Technology	99940
CD8α	Cell Signaling Technology	98941
CDC7	Cell Signaling Technology	3603
cGAS	Cell Signaling Technology	83623
cGAS (mouse specific)	Cell Signaling Technology	31659
c-Myc	Cell Signaling Technology	18583
cleaved caspase-3	Cell Signaling Technology	9661
cleaved PARP	Cell Signaling Technology	5625
CNOT1	Cell Signaling Technology	44613
FoxP3	Cell Signaling Technology	12653
HA	Cell Signaling Technology	3724
HP1γ	Cell Signaling Technology	2619
IFI16	Cell Signaling Technology	14970
IRF3	Cell Signaling Technology	4302
K48-linkage specific polyubiquitin	Cell Signaling Technology	4289
K63-linkage specific polyubiquitin	Cell Signaling Technology	5621
MAVS	Cell Signaling Technology	24930
MDA5	Cell Signaling Technology	5321
METTL3	Cell Signaling Technology	86132
Myc	Cell Signaling Technology	2278
PD-L1	Cell Signaling Technology	13684
PD-L1 (mouse specific)	Cell Signaling Technology	60475
p-Chk1 (S345)	Cell Signaling Technology	2348
p-Histone H2A.X (Ser139)	Cell Signaling Technology	9718
p-IRF-3 (Ser386)	Cell Signaling Technology	37829
p-IRF-3 (Ser396)	Cell Signaling Technology	29047
p-STING (Ser366)	Cell Signaling Technology	50907
p-TBK1 (Ser172)	Cell Signaling Technology	5483
PTEN	Cell Signaling Technology	9559
RIG-I	Cell Signaling Technology	3743
SNARK/NUAK2	Cell Signaling Technology	15452
STING	Cell Signaling Technology	13647
TBK1	Cell Signaling Technology	38066
UBE2O	Cell Signaling Technology	83393
ubiquitin	Cell Signaling Technology	3936
rabbit IgG, HRP-linked	Cell Signaling Technology	7074
mouse IgG, HRP-linked	Cell Signaling Technology	7076
Cul3	Santa Cruz Biotechnology	sc-166110
GAPDH	Santa Cruz Biotechnology	sc-47724
GST	Santa Cruz Biotechnology	sc-459
vinculin	Santa Cruz Biotechnology	sc-25336
CtIP	Sigma-Aldrich	MABE1060

Flag	Sigma-Aldrich	F1825
Flag	Sigma-Aldrich	F7425
Tubulin	Sigma-Aldrich	T5168
ARL8A	Proteintech	17060-1-AP
BMI1	Proteintech	66161-1-Ig
DDX41	Proteintech	27500-1-AP
dsRNA	Proteintech	85780-2-RR
ESCO2	Proteintech	23525-1-AP
GNE	Proteintech	25079-1-AP
HA	Proteintech	51064-2-AP
His-Tag	Proteintech	66005-1-Ig
Histone H2A.X	Proteintech	10856-1-AP
Myc tag	Proteintech	60003-2-Ig
SPOP	Proteintech	16750-1-AP
dsDNA	Novus Biologicals	NBP3-07670
APC anti-mouse CD3 Antibody	BioLegend	100236
Brilliant Violet 421 anti-mouse CD4 Antibody	BioLegend	100437
Brilliant Violet 785 anti-mouse CD8a Antibody	BioLegend	100750
APC/Cyanine7 anti-mouse/human CD11b Antibody	BioLegend	101226
PE/Cyanine7 anti-mouse CD11c Antibody	BioLegend	117317
PerCP anti-mouse CD19 Antibody	BioLegend	115531
Brilliant Violet 711 anti-mouse CD45 Antibody	BioLegend	103147
Brilliant Violet 605 anti-mouse CD80 Antibody	BioLegend	104729
Brilliant Violet 421 anti-mouse CD206 Antibody	BioLegend	141717
PE/Cyanine5 anti-mouse F4/80 Antibody	BioLegend	123111
PE anti-mouse IFN-γ Antibody	BioLegend	505808
Alexa Fluor 700 anti-human/mouse Granzyme B Recombinant Antibody	BioLegend	372221
Alexa Fluor 488 goat anti-rabbit IgG (H+L)	ThermoFisher Scientific	A11034
Alexa Fluor 594 goat anti-mouse IgG (H+L)	ThermoFisher Scientific	A11032
