# Supplementary material for: Molecular glue degrader function of SPOP inhibitors enhances STING-dependent immunotherapy efficacy in melanoma models
Source: J Clin Invest. 2025 Oct 28;135(24):e191772. doi: 10.1172/JCI191772 (PMC12700557; doi:10.1172/JCI191772)

Figure 1

Full unedited blot for Figure 1E

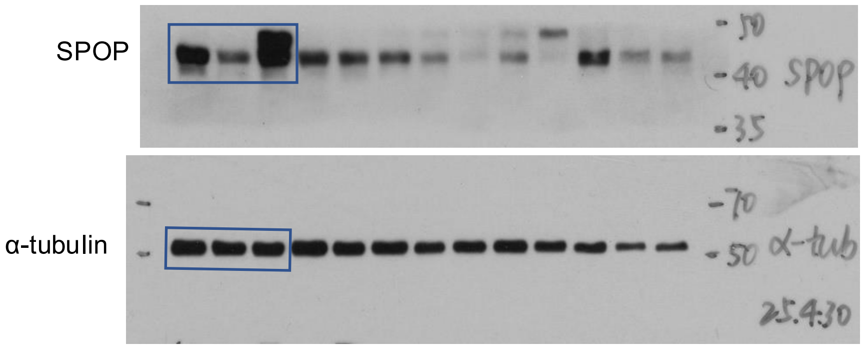

Figure 2

Full unedited blot for Figure 2A

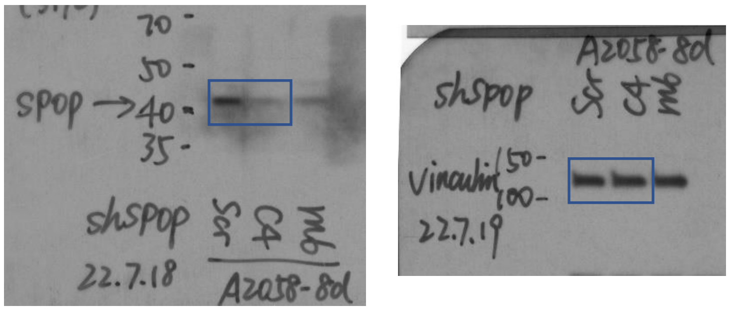

Full unedited blot for Figure 2D

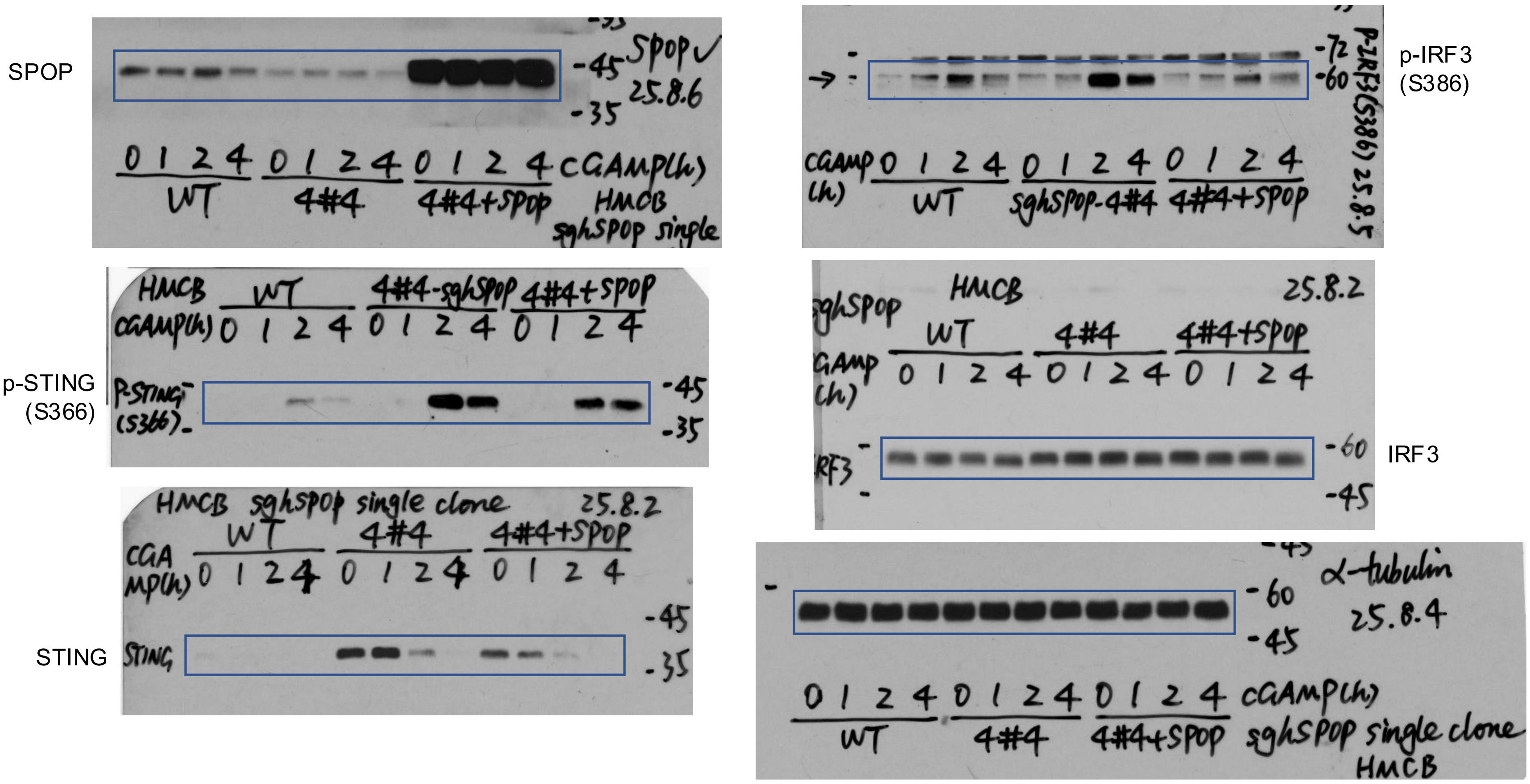

Figure 3

Full unedited blot for Figure 3A

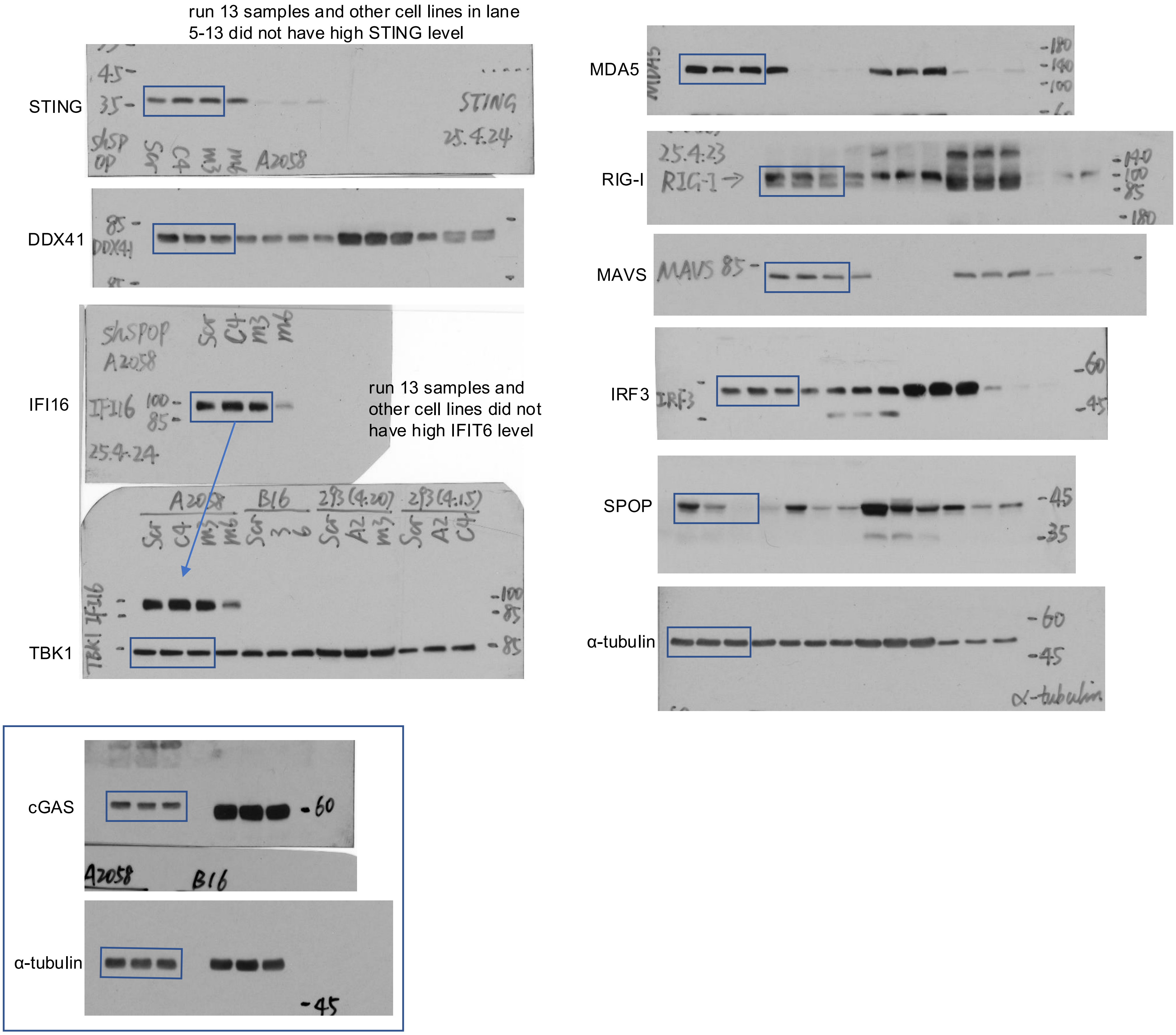

Full unedited blot for Figure 3B

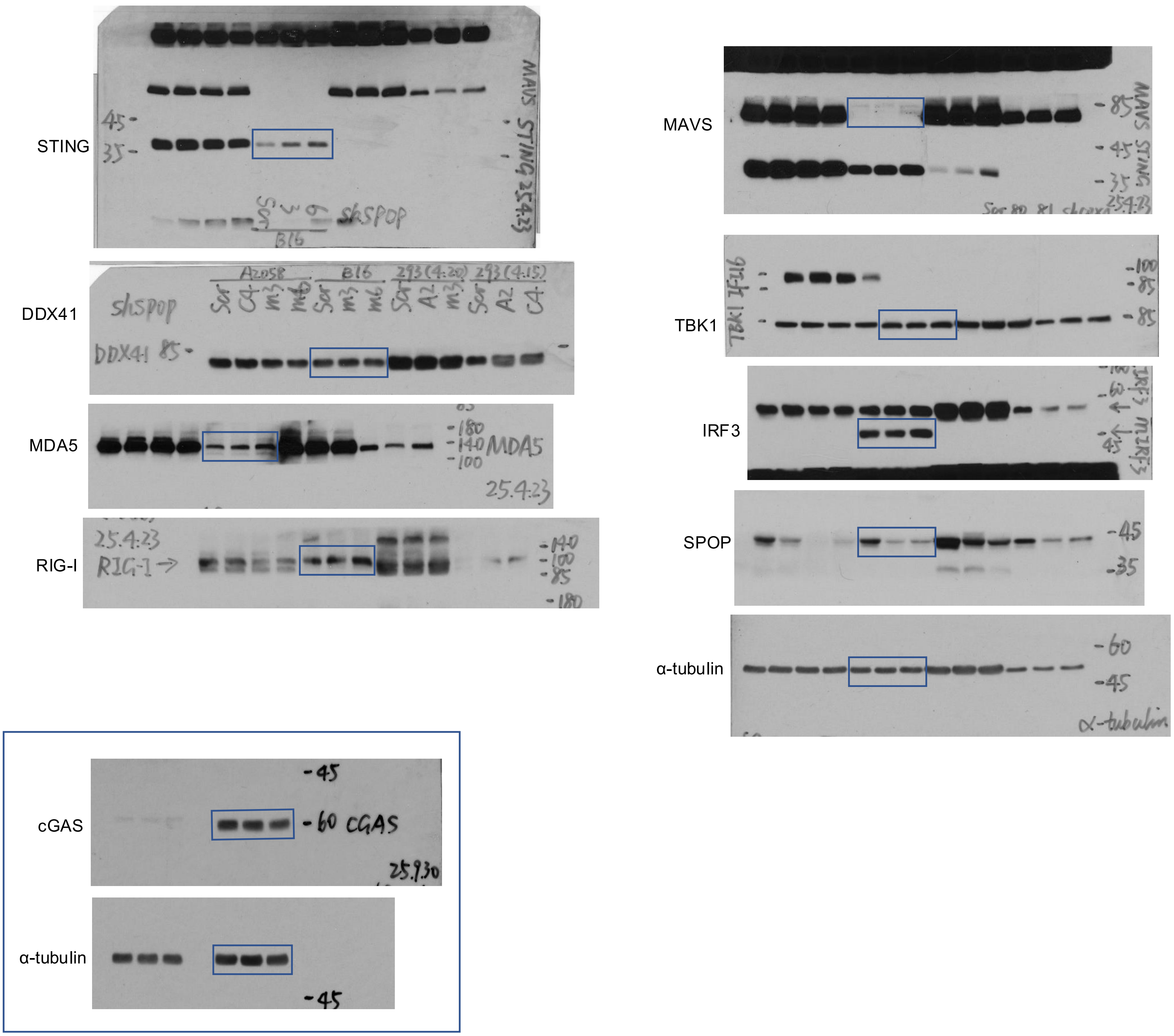

Full unedited blot for Figure 3C

STING

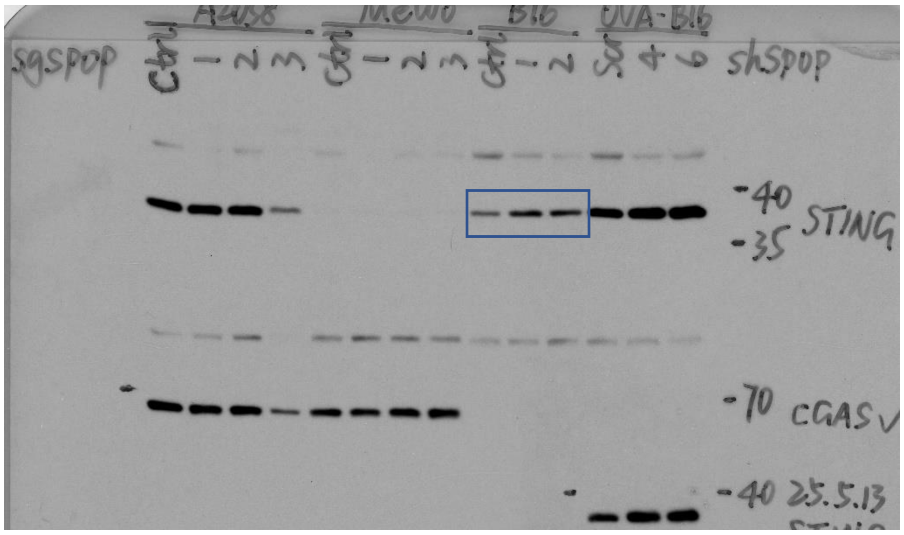

IRF3

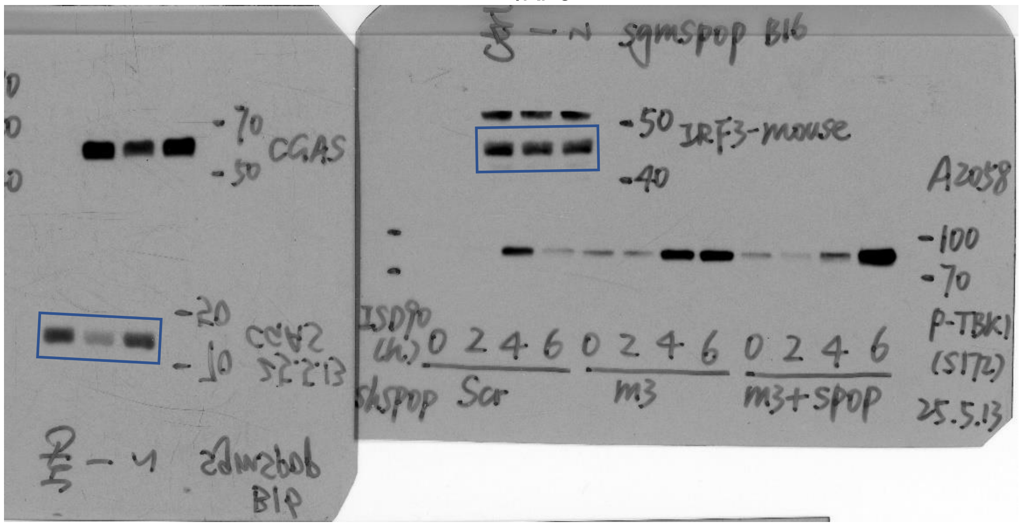

Three B16 samples were run in parallel gel on the same day for cGAS and IRF3 detection.

cGAS

SPOP

TBK1

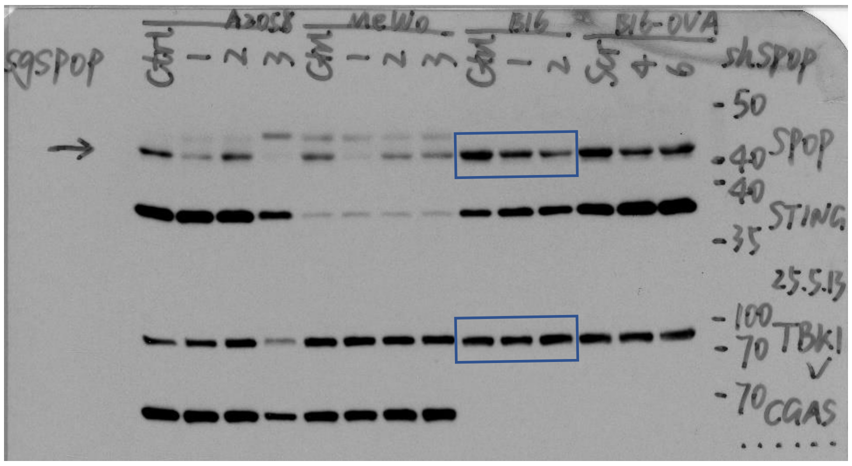

α-tubulin

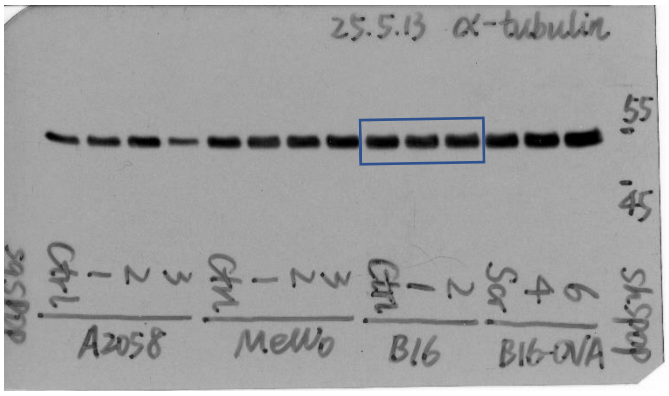

Full unedited blot for Figure 3D

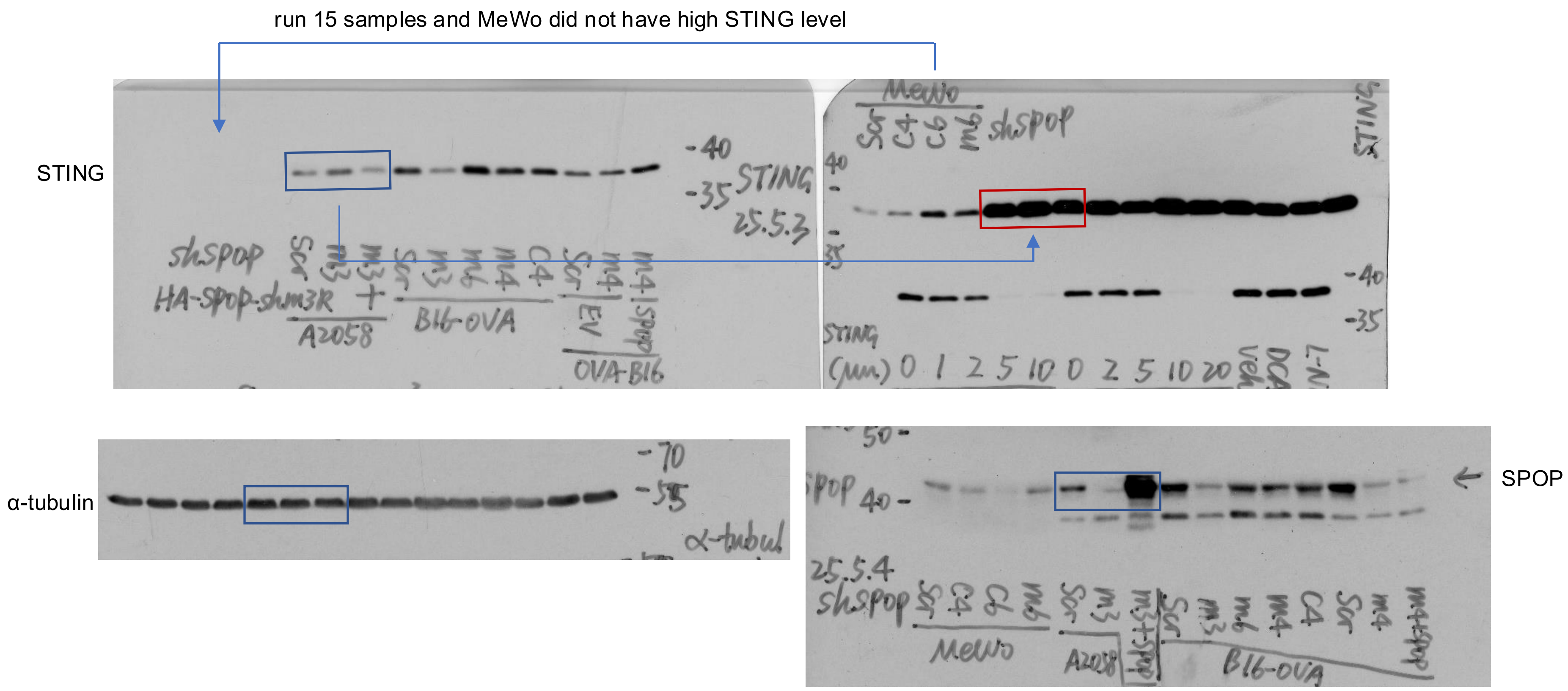

Full unedited blot for Figure 3E

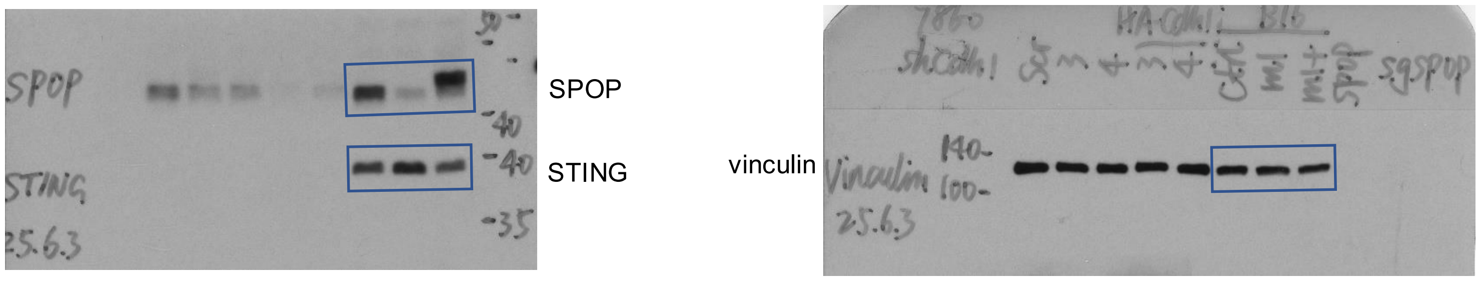

Full unedited blot for Figure 3F and 3G

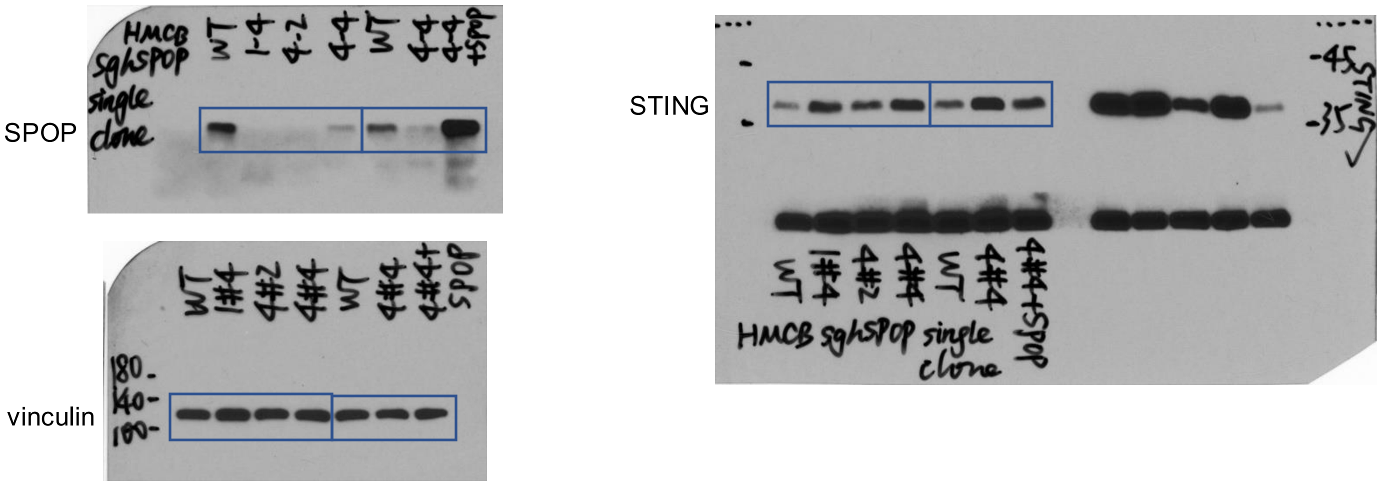

Full unedited blot for Figure 3H

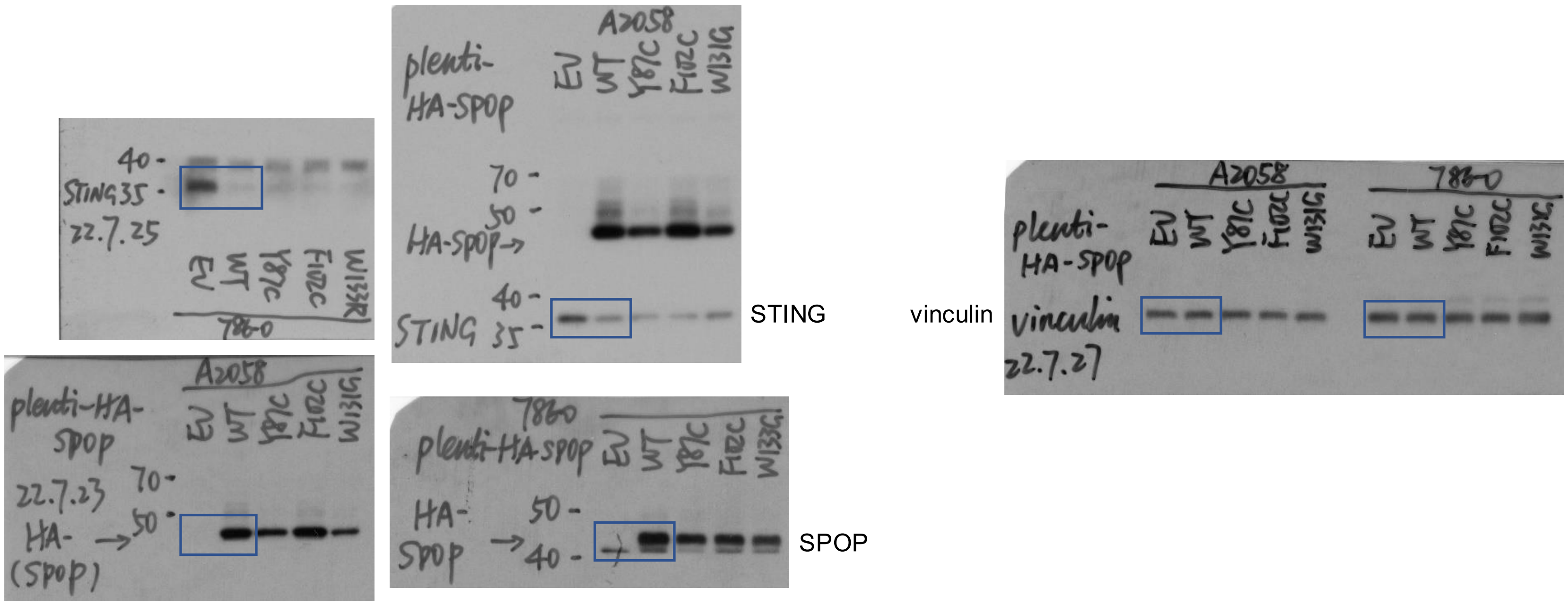

Full unedited blot for Figure 3I

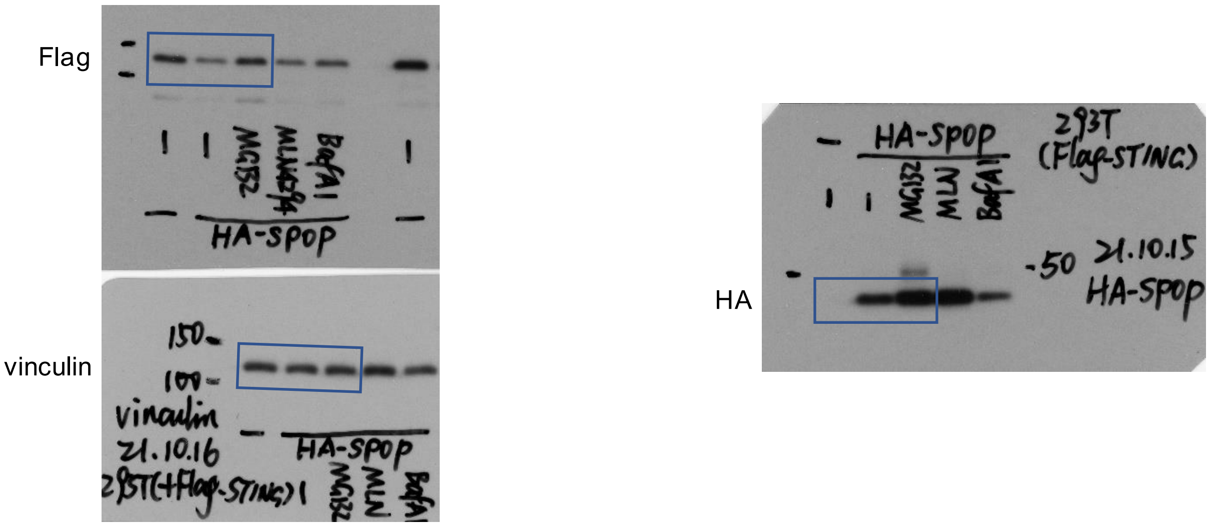

Full unedited blot for Figure 3J

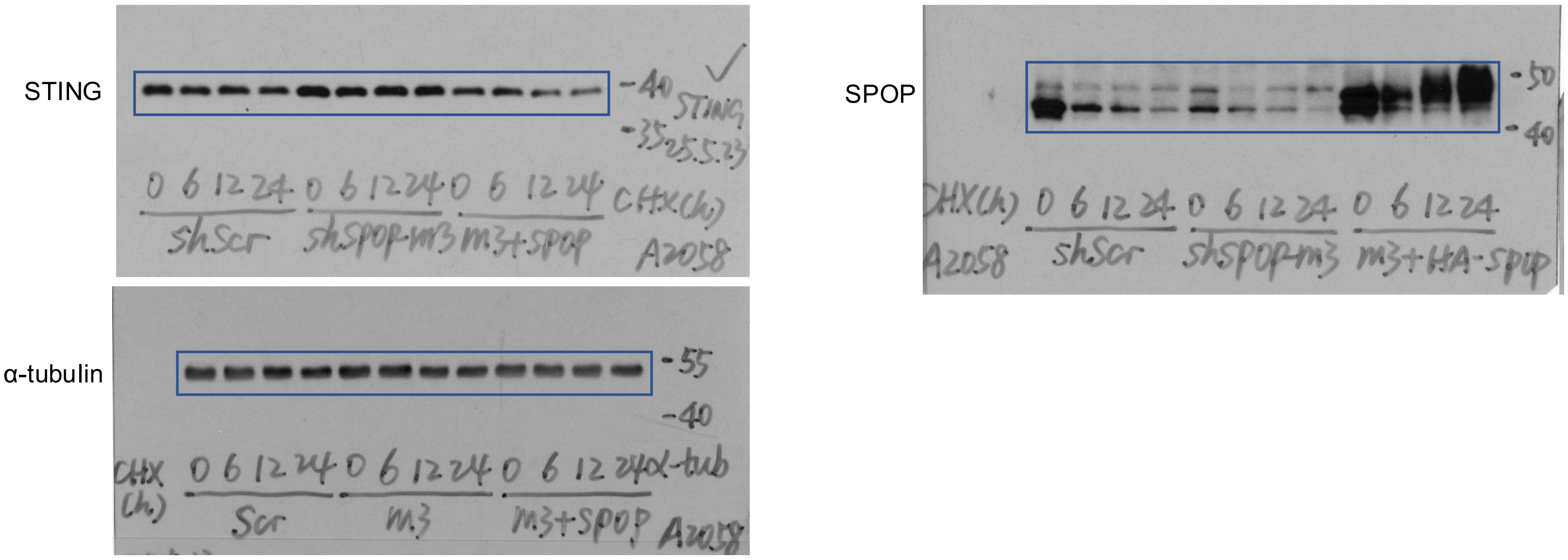

Full unedited blot for Figure 3M

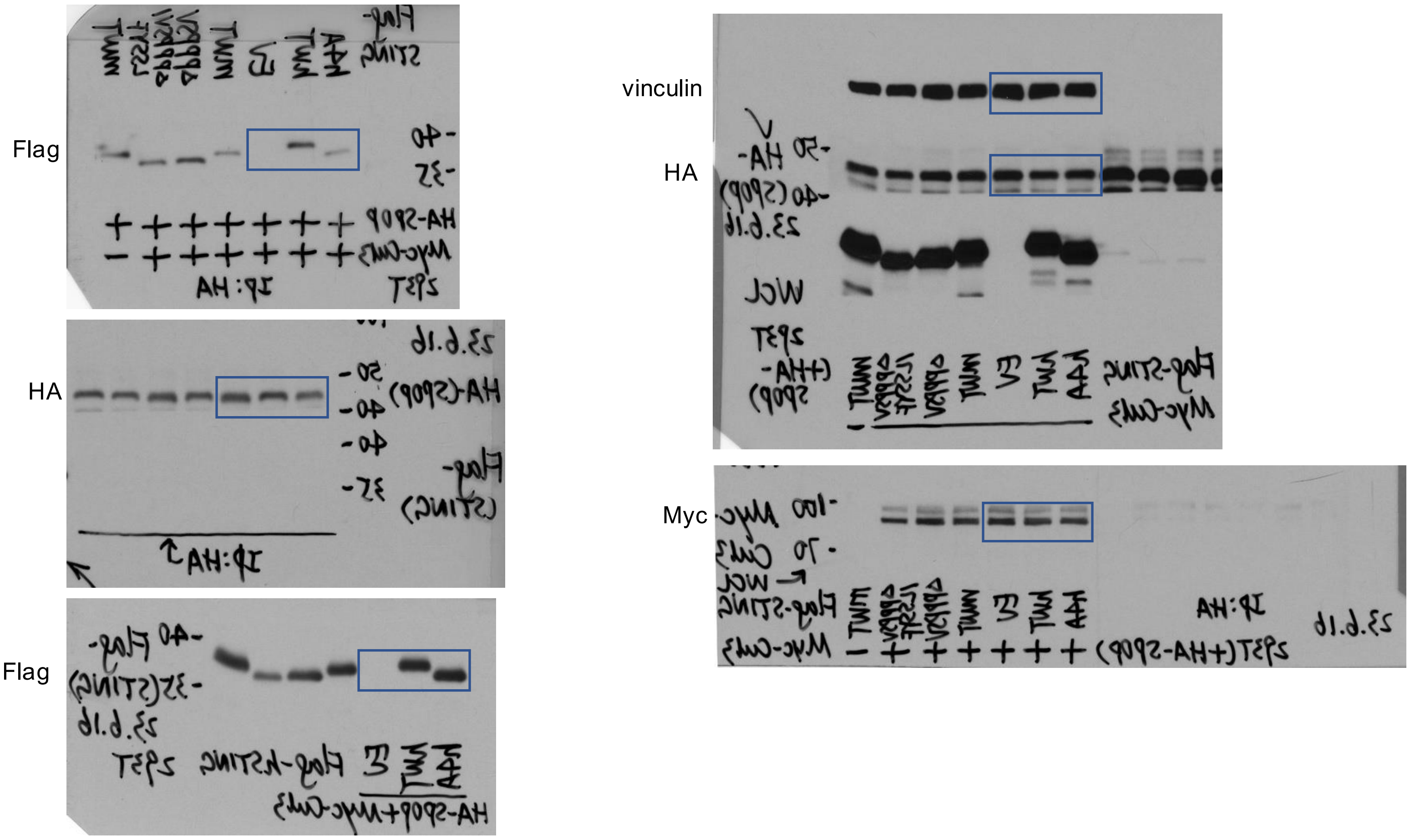

Full unedited blot for Figure 3N

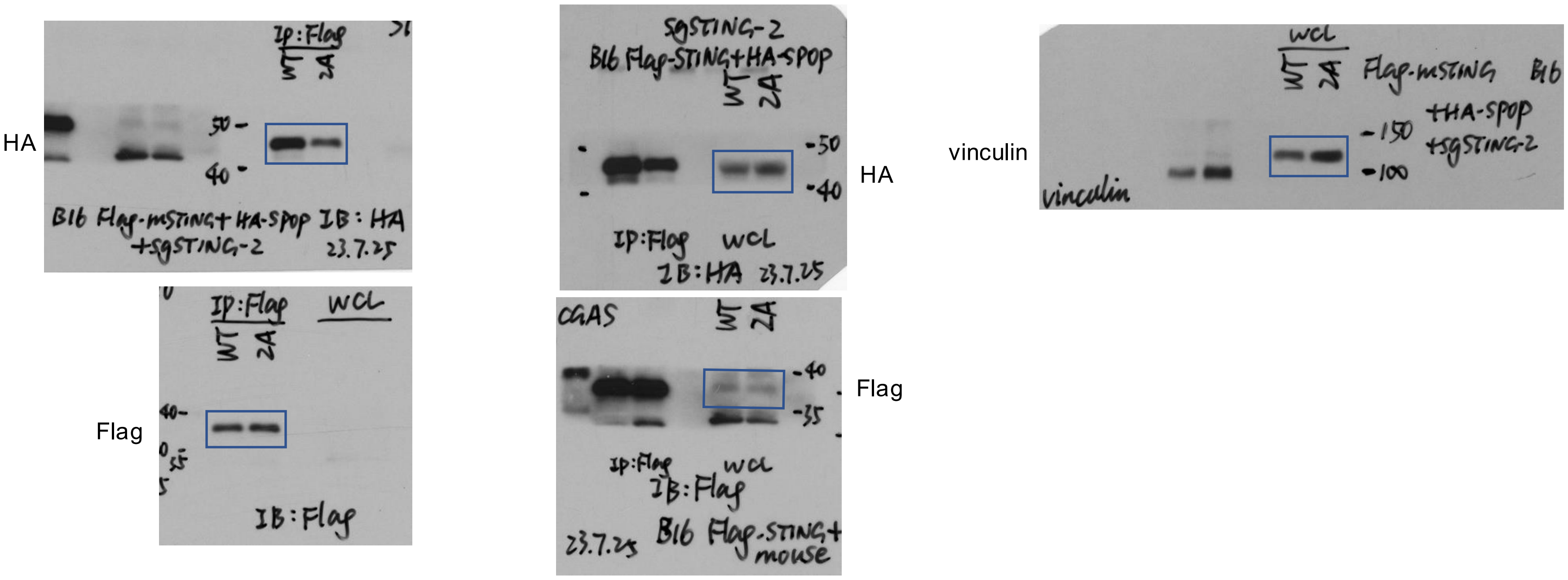

Full unedited blot for Figure 3O

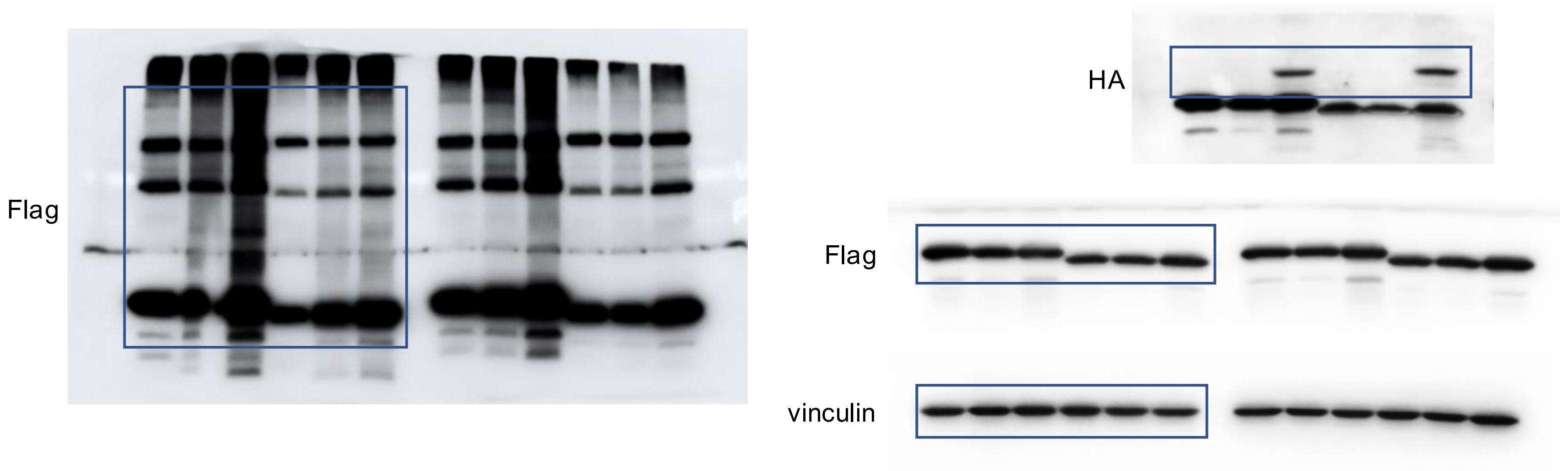

Figure 4

Full unedited blot for Figure 4A

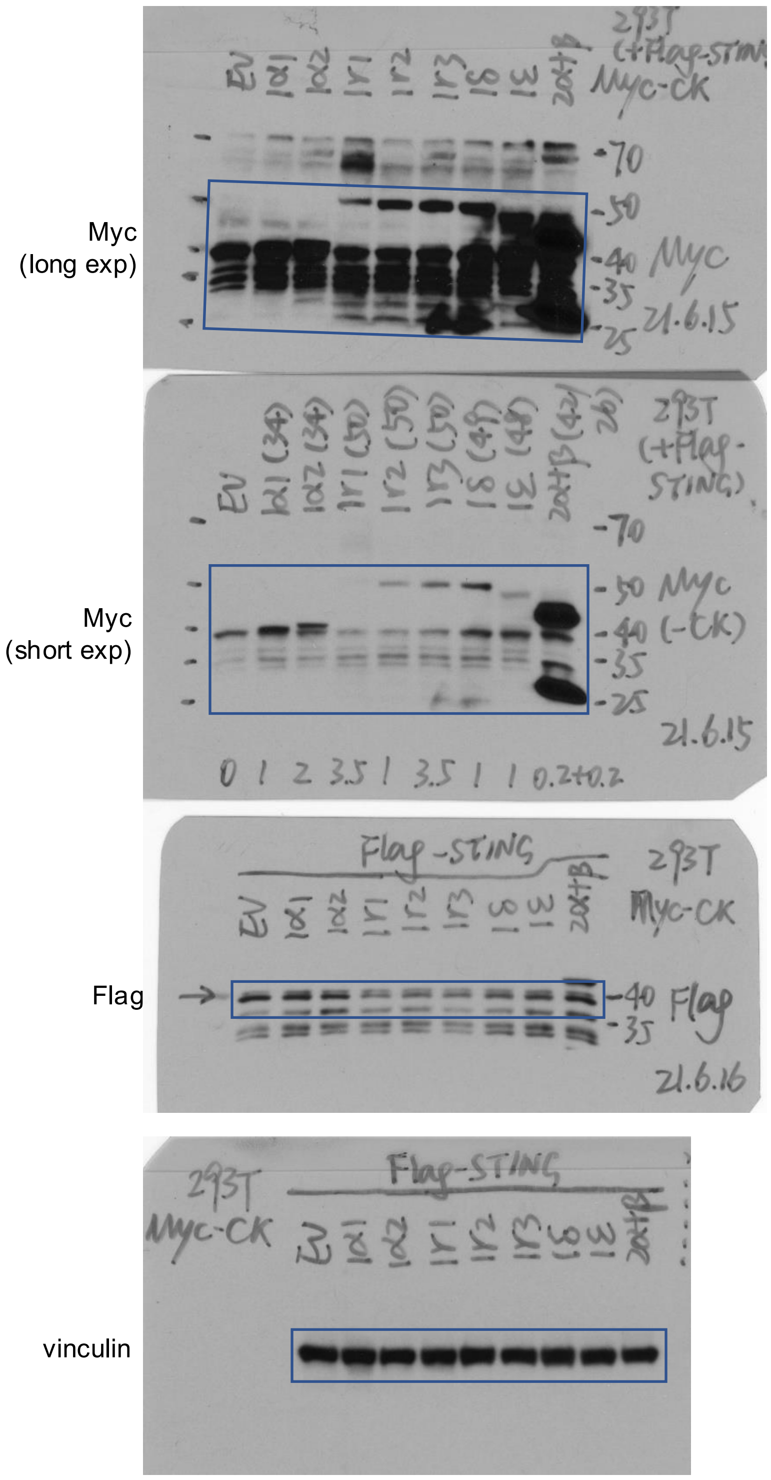

Full unedited blot for Figure 4B

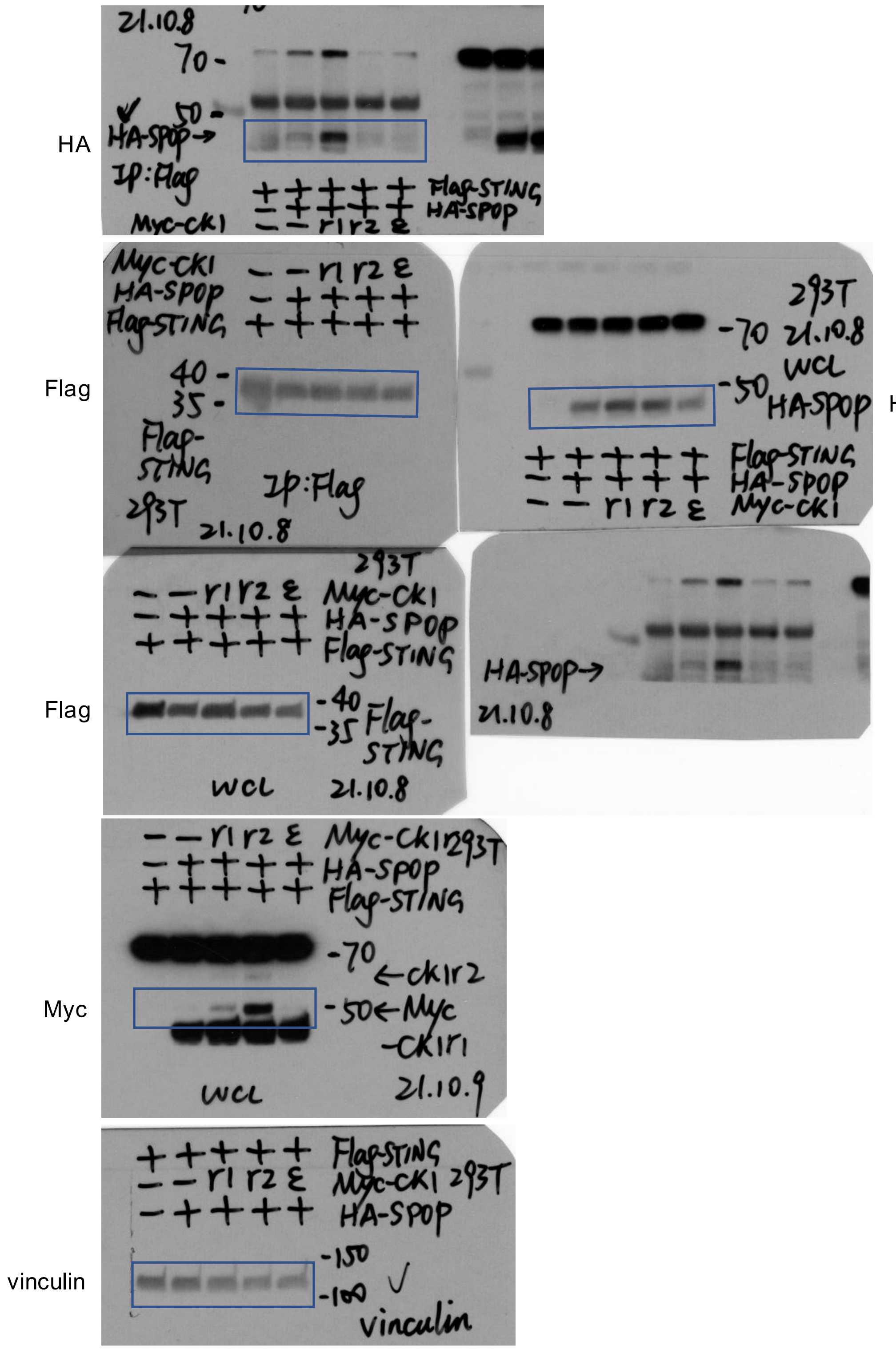

Full unedited blot for Figure 4C

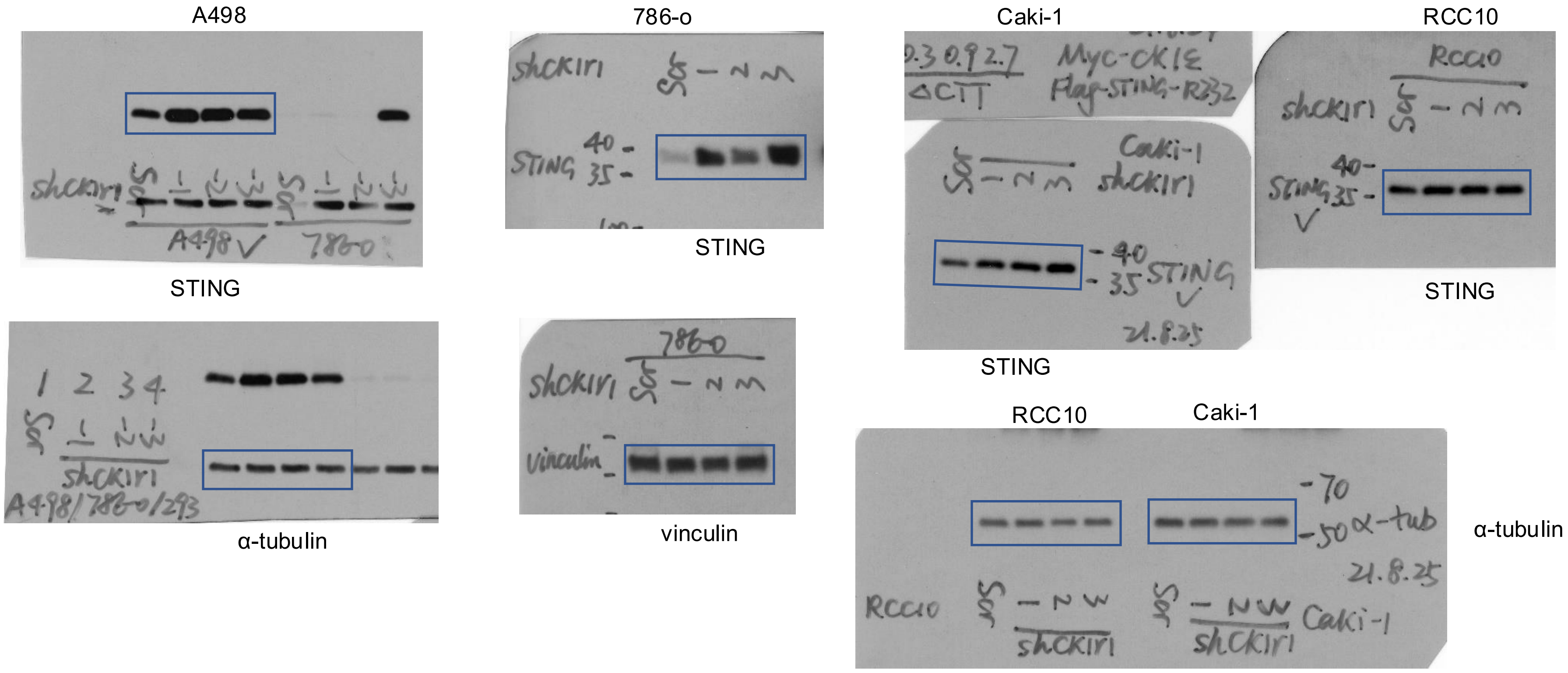

Full unedited blot for Figure 4F

Flag

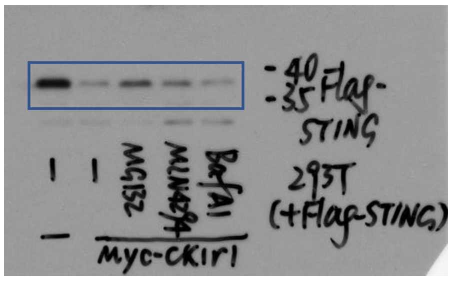

Myc

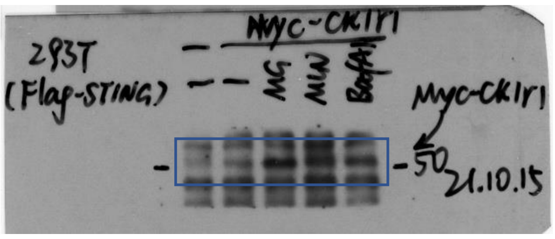

vinculin

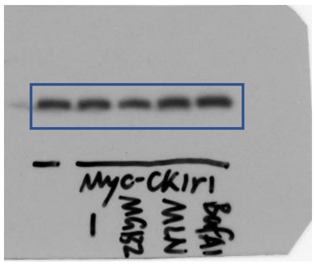

Full unedited blot for Figure 4G

Flag

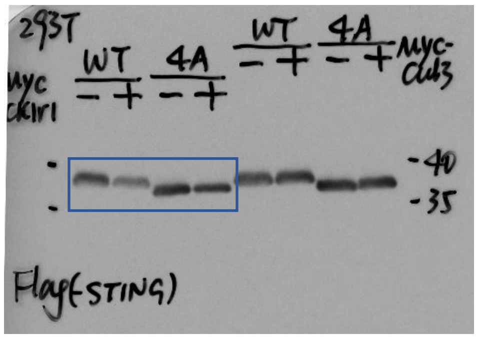

Myc

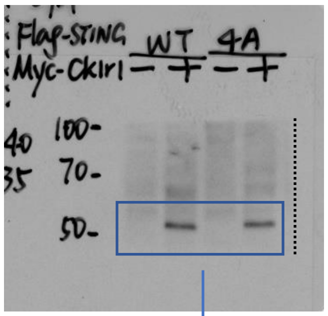

vinculin

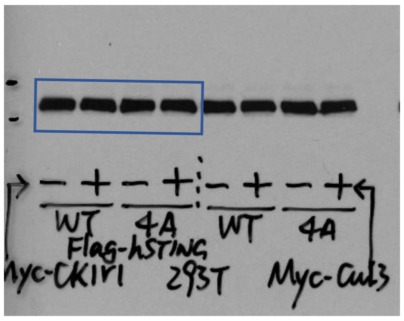

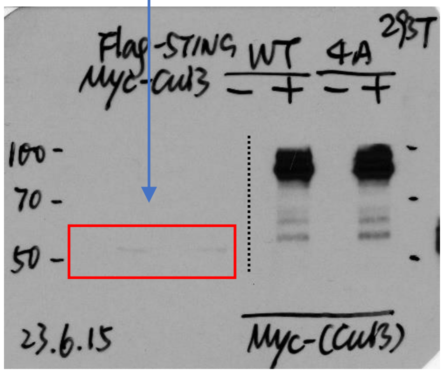

Left half of the membrane was used for longer exposure.

Full unedited blot for Figure 4H

A498

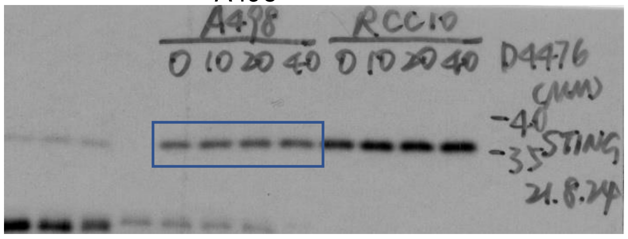

STING

786-o

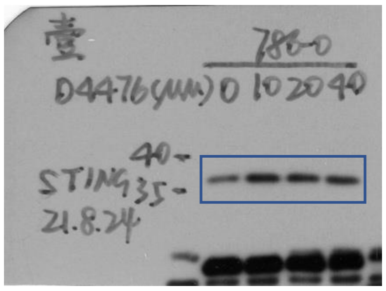

STING

786-o A498

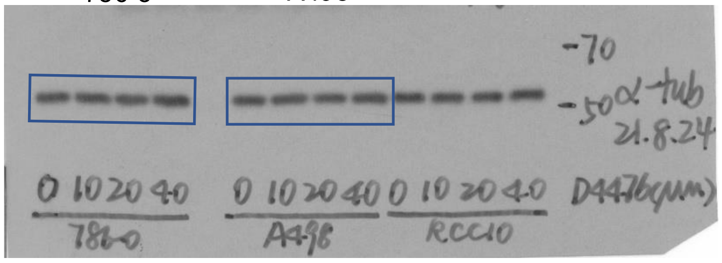

α-tubulin

Full unedited blot for Figure 4I

786-o

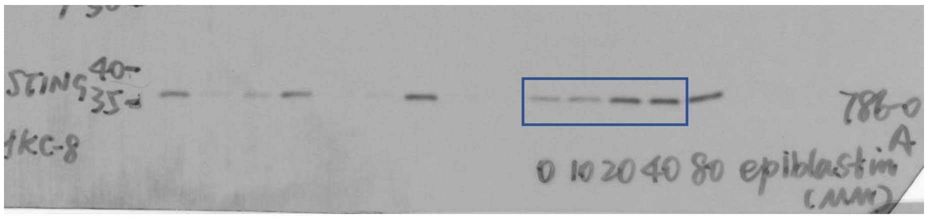

STING

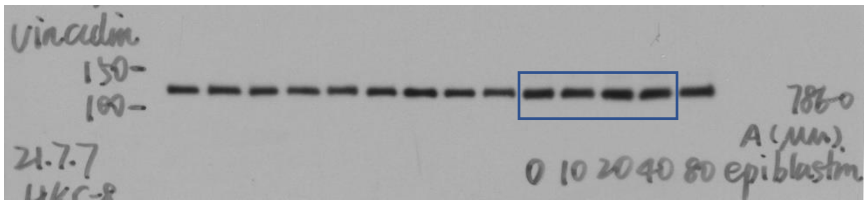

vinculin

HEK293

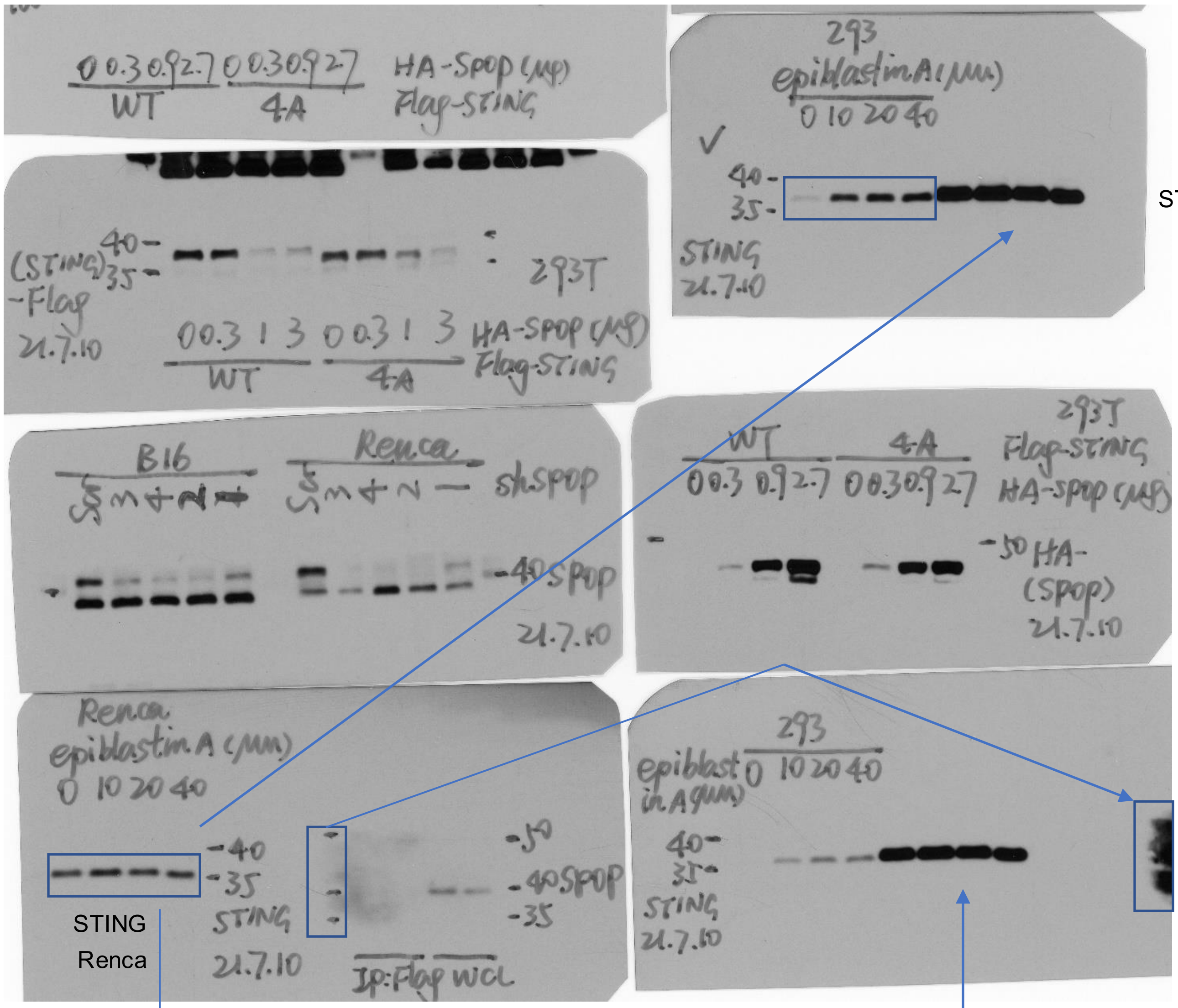

STING

HEK293 part was cut off because of no STING signal in the shorter exposure film.

HEK293 Renca

vinculin

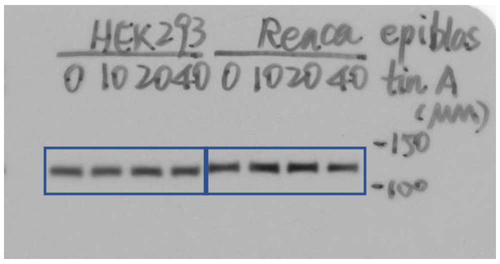

Figure 5

Full unedited blot for Figure 5A

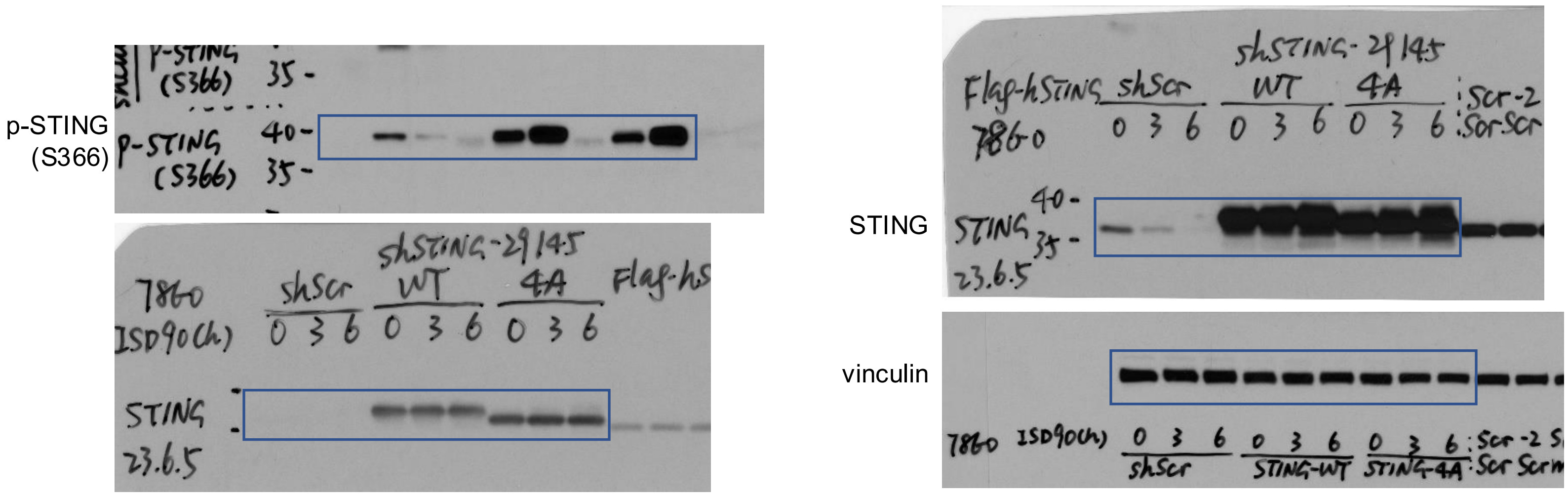

Full unedited blot for Figure 5E

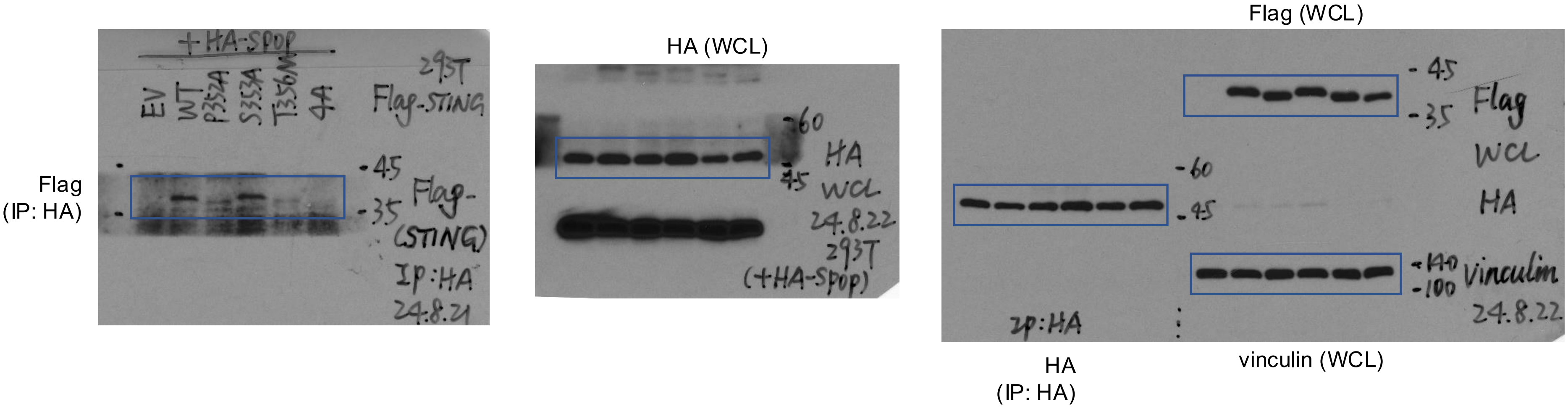

Full unedited blot for Figure 5F

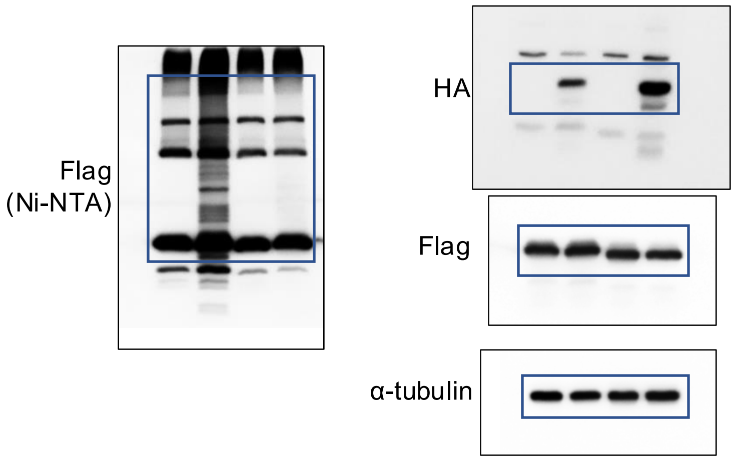

Full unedited blot for Figure 5G

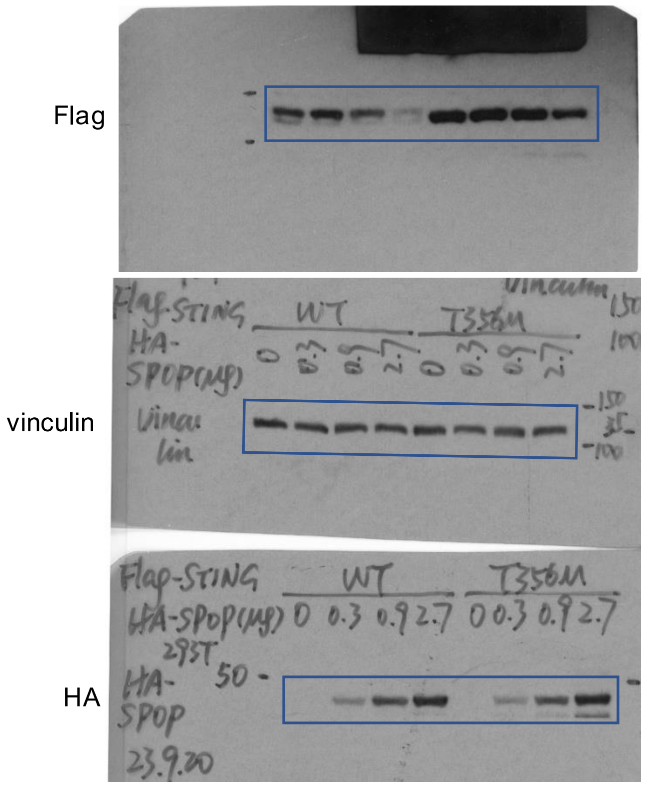

Full unedited blot for Figure 5H

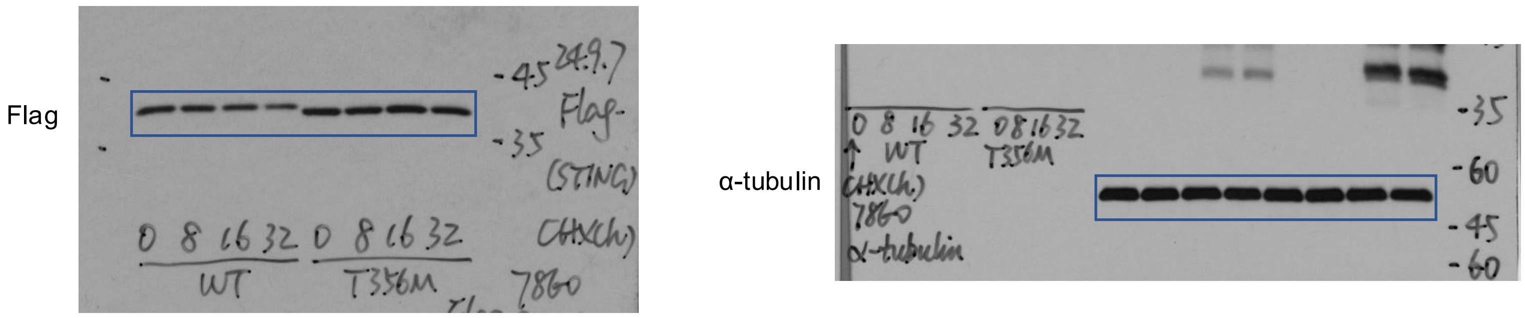

Figure 6

Full unedited blot for Figure 6A

p-STING (S366)

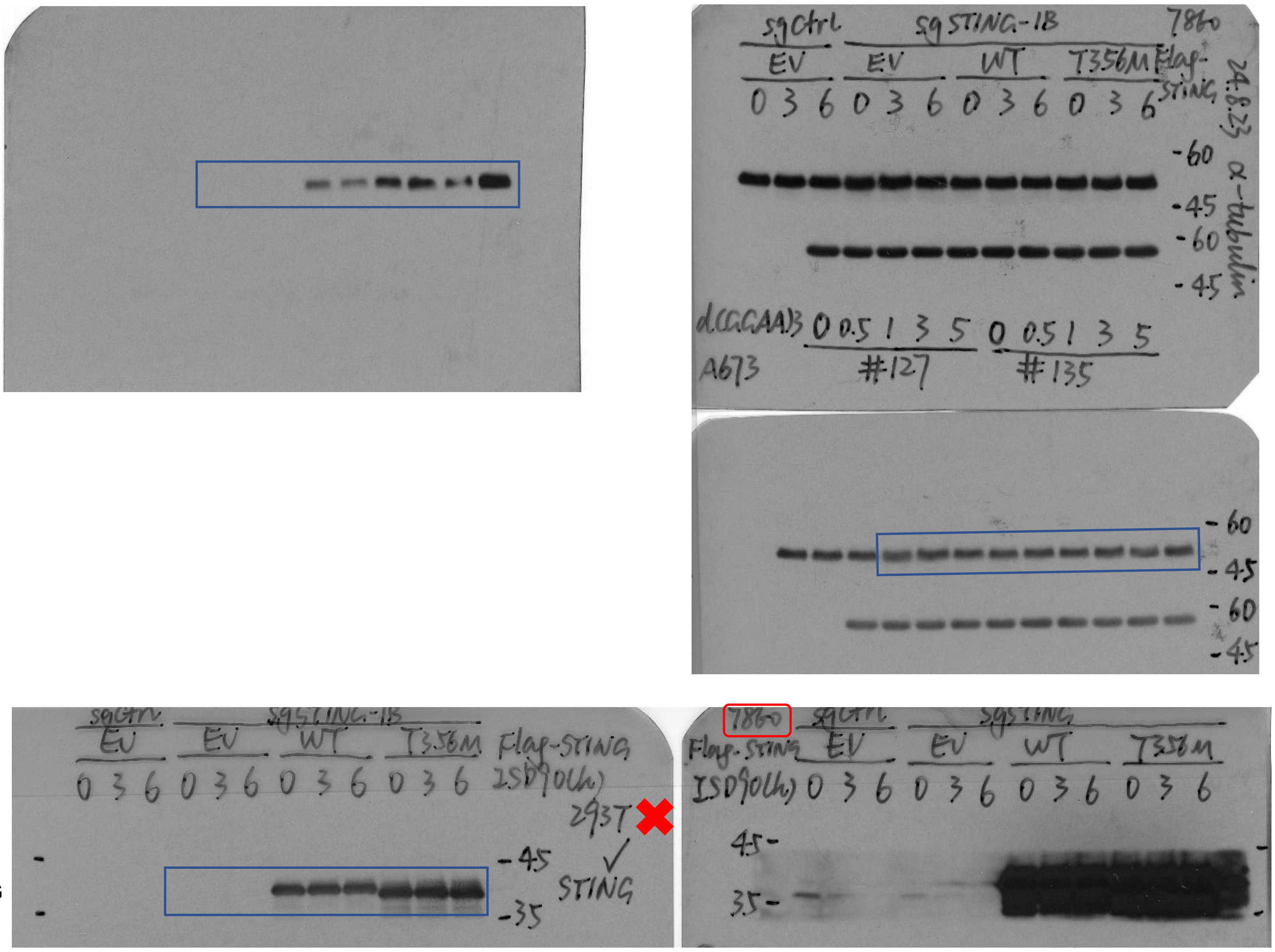

α-tubulin

STING

Cell line name “293T” in the short exposure film was typo error of “786-o”.

Full unedited blot for Figure 6B

p-STING (S366)

p-IRF3 (S386)

Flag

IRF3

vinculin

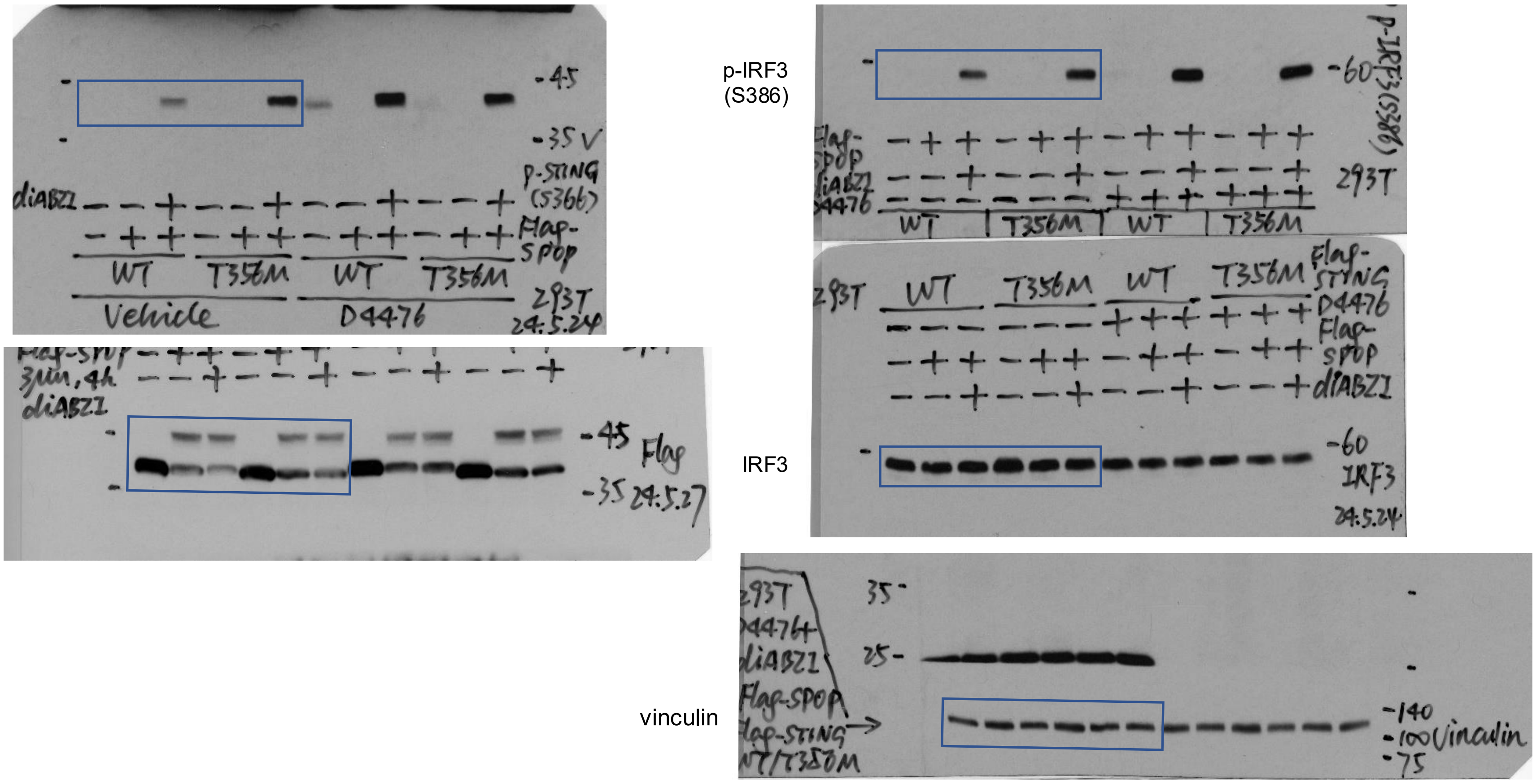

Full unedited blot for Figure 6D

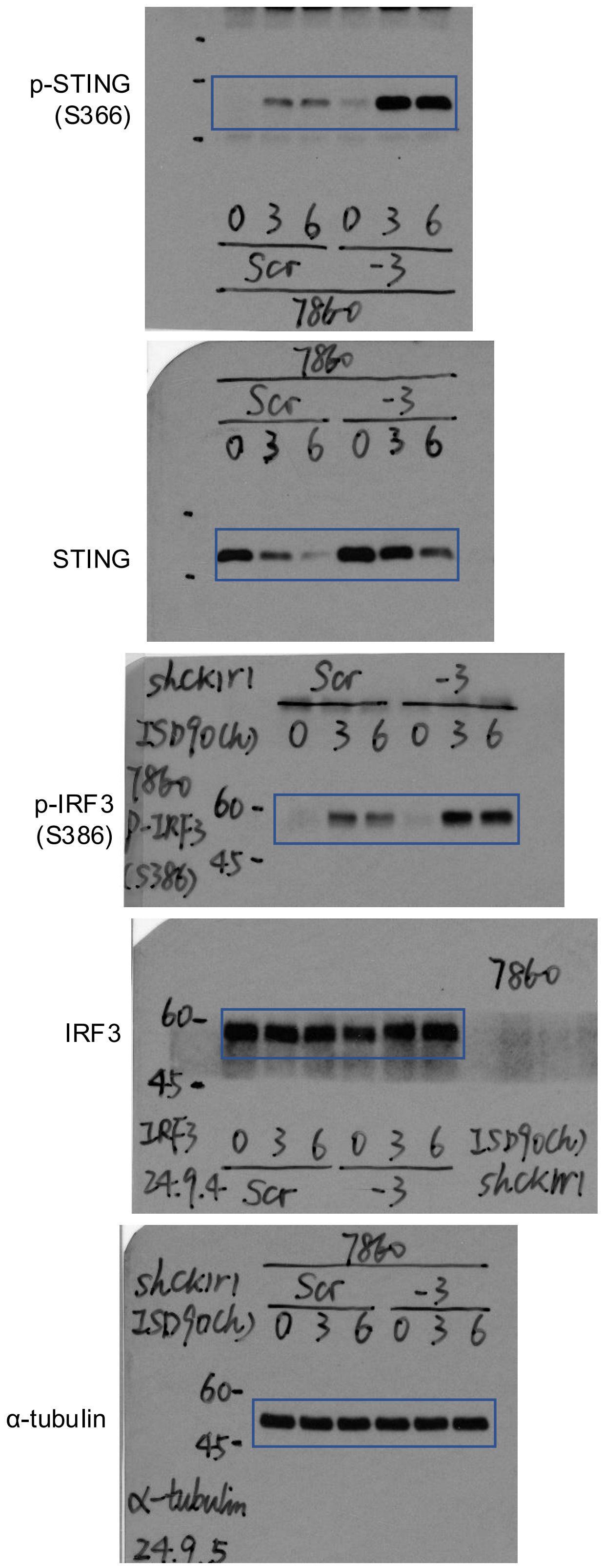

Full unedited blot for Figure 6E

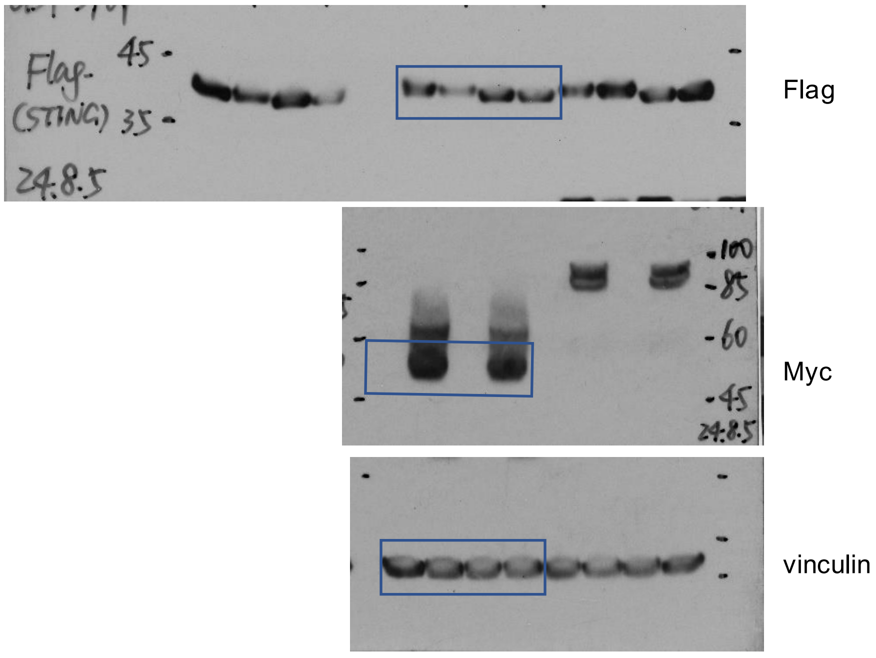

Full unedited blot for Figure 6F

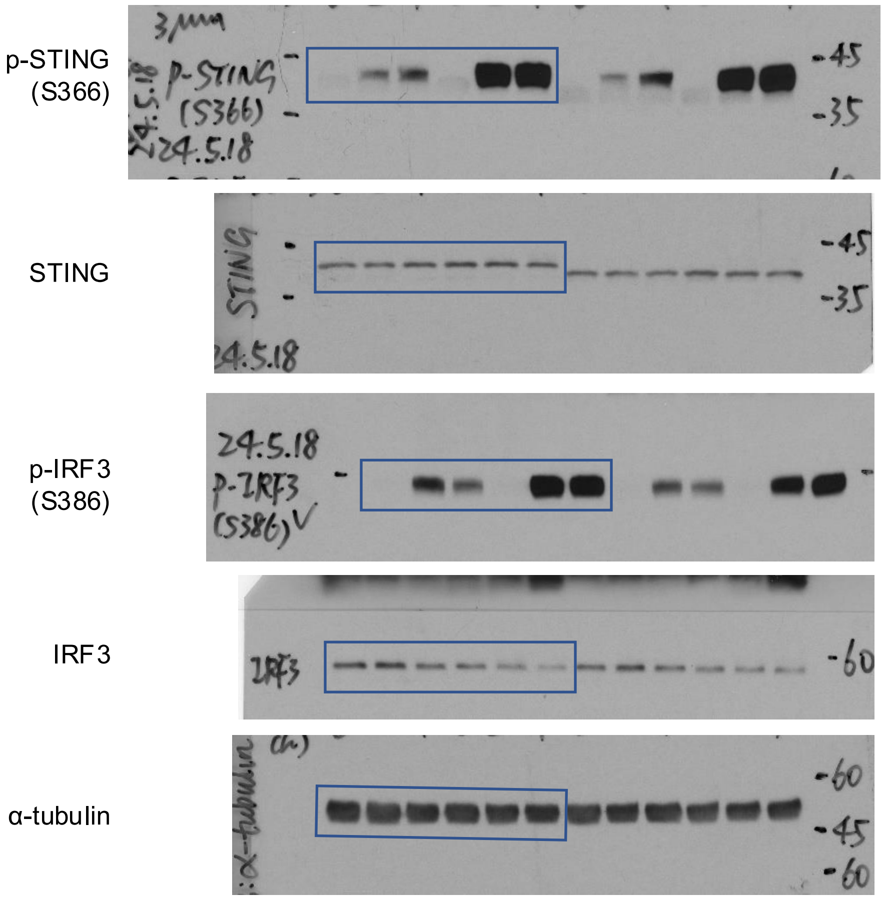

Figure 7

Full unedited blot for Figure 7A

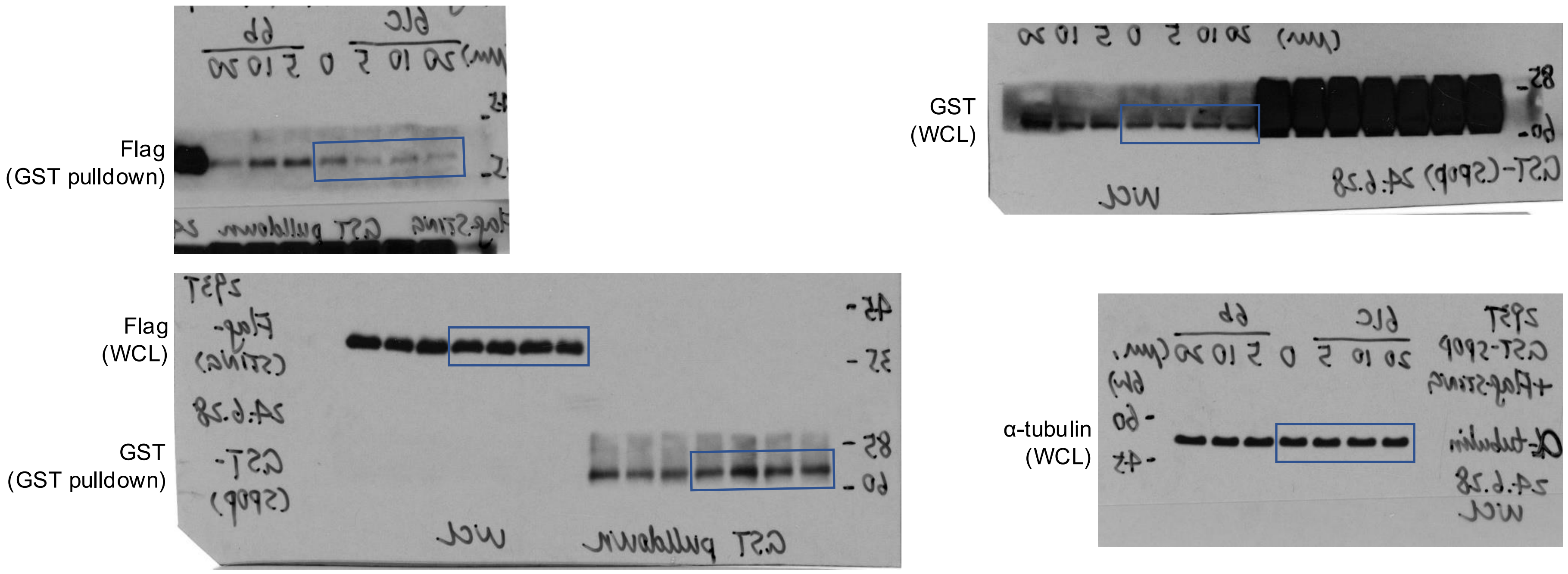

Full unedited blot for Figure 7B

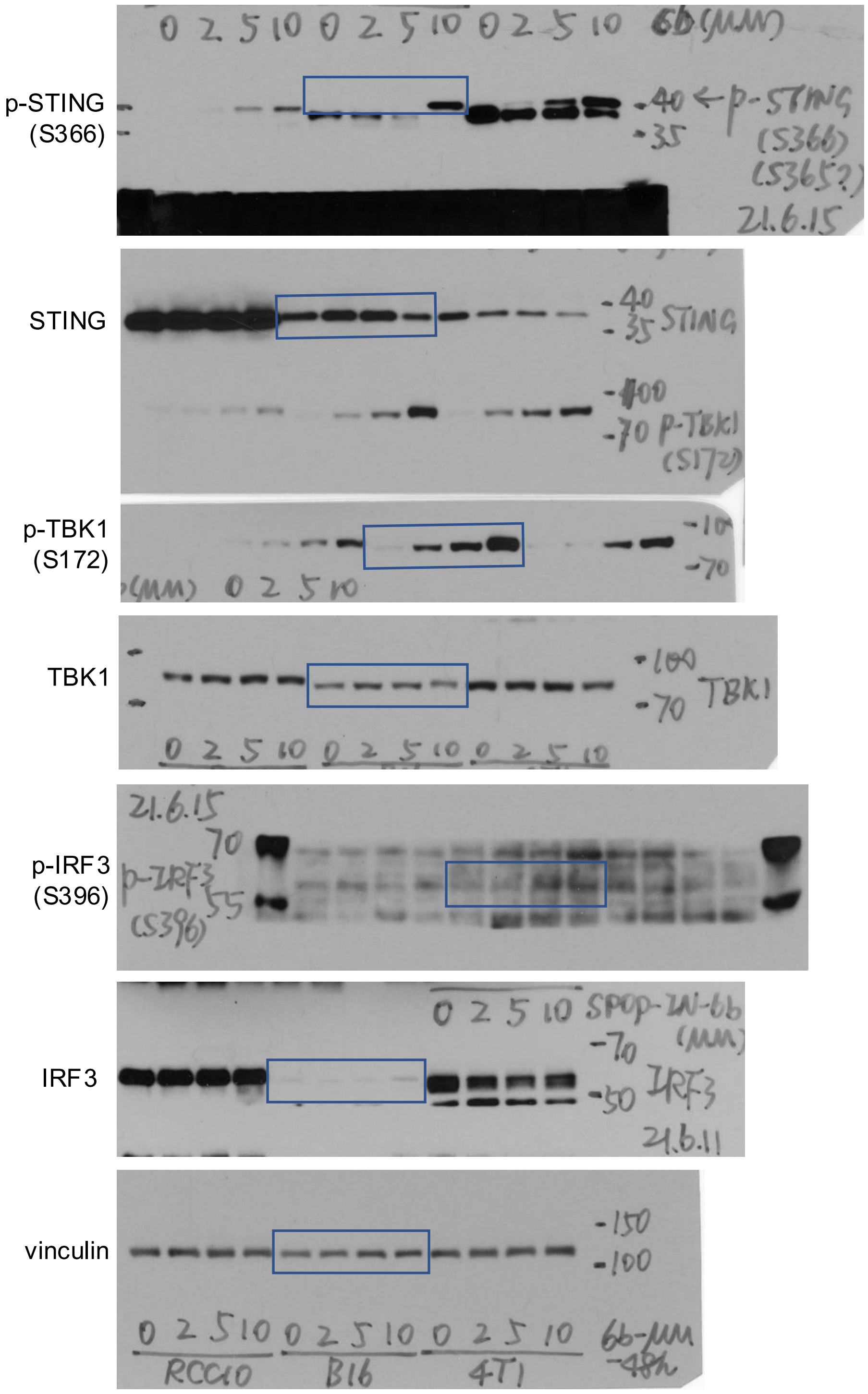

Full unedited blot for Figure 7C

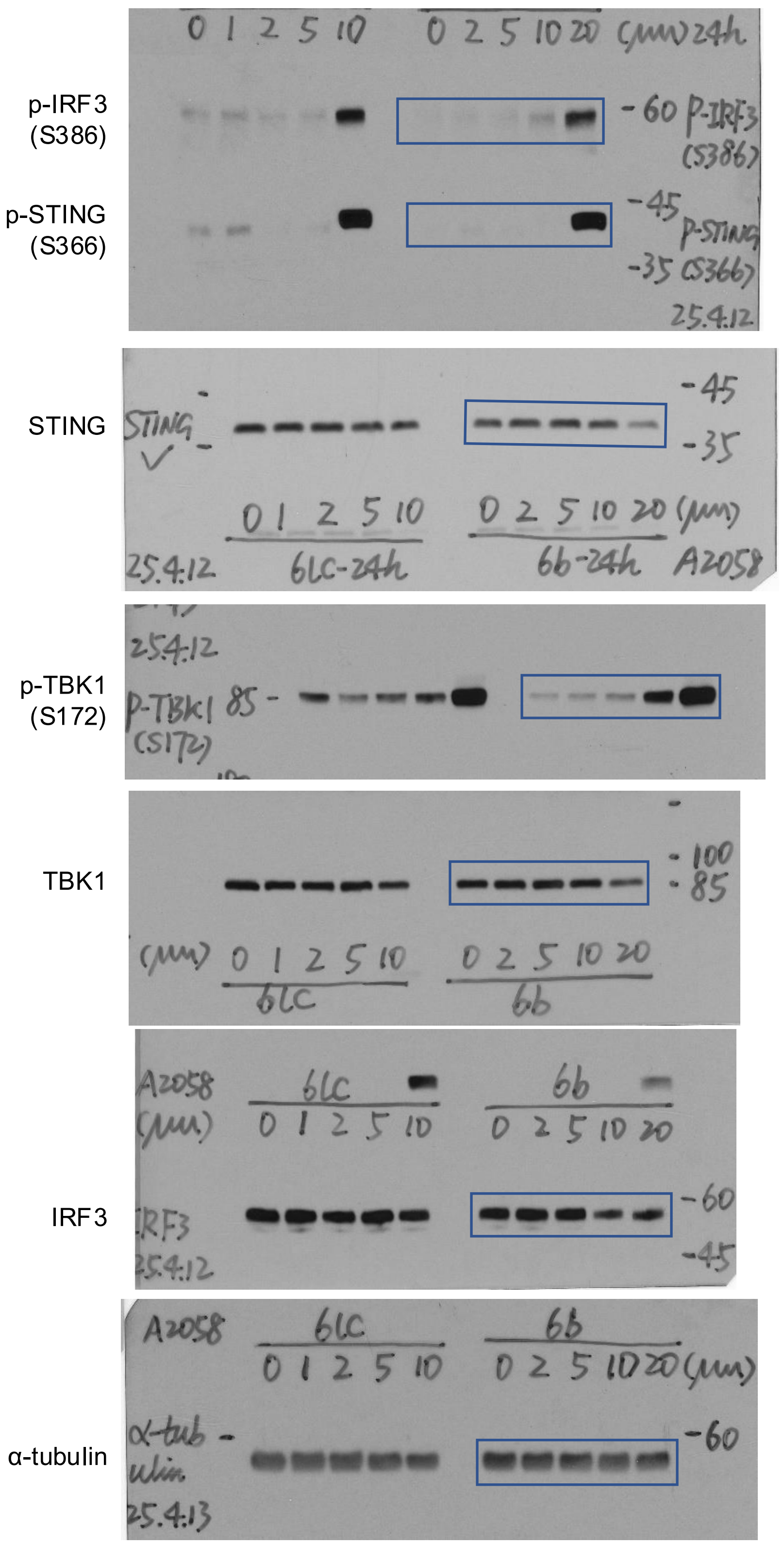

Figure 9

Full unedited blot for Figure 9D

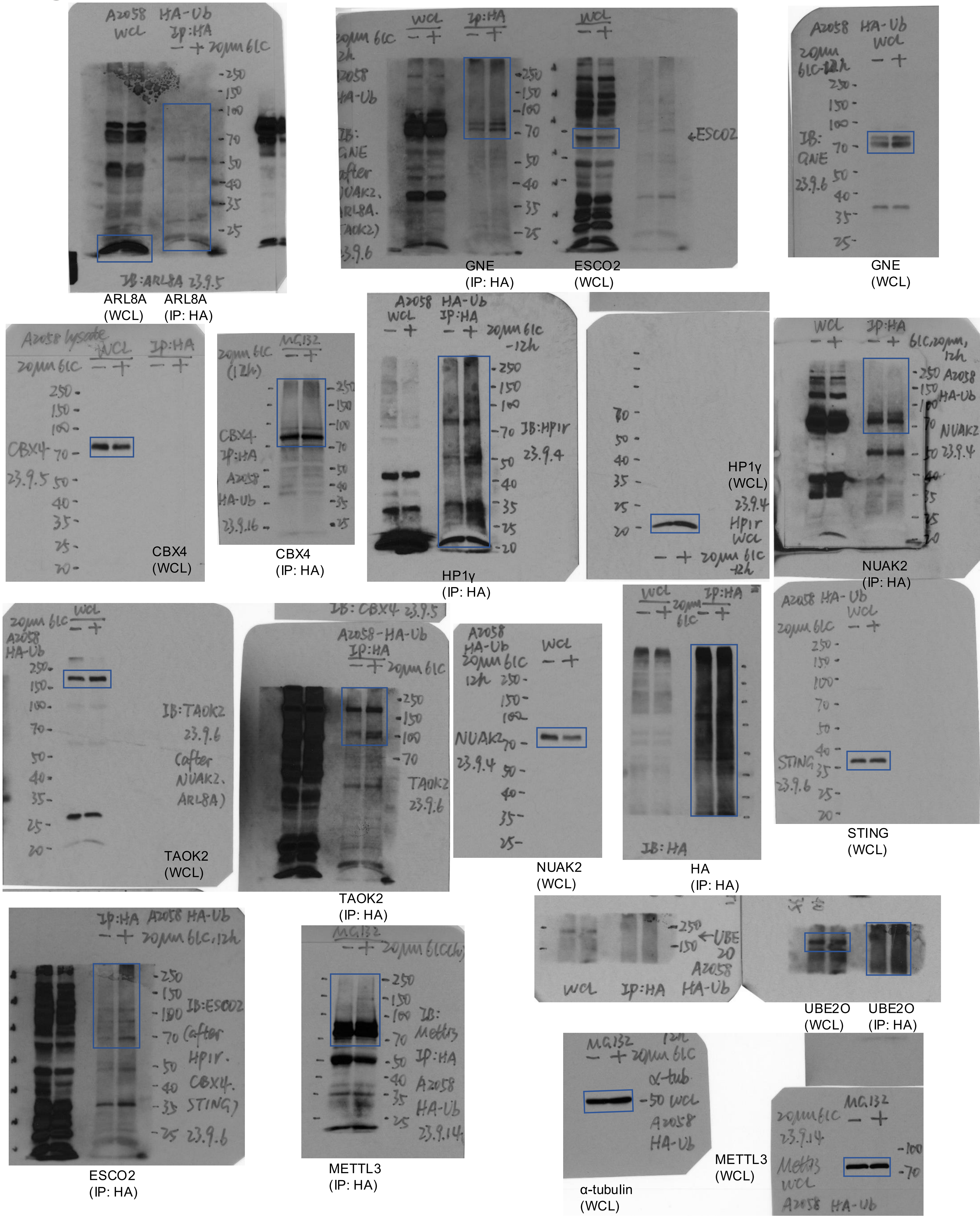

Figure 10

Full unedited blot for Figure 10A

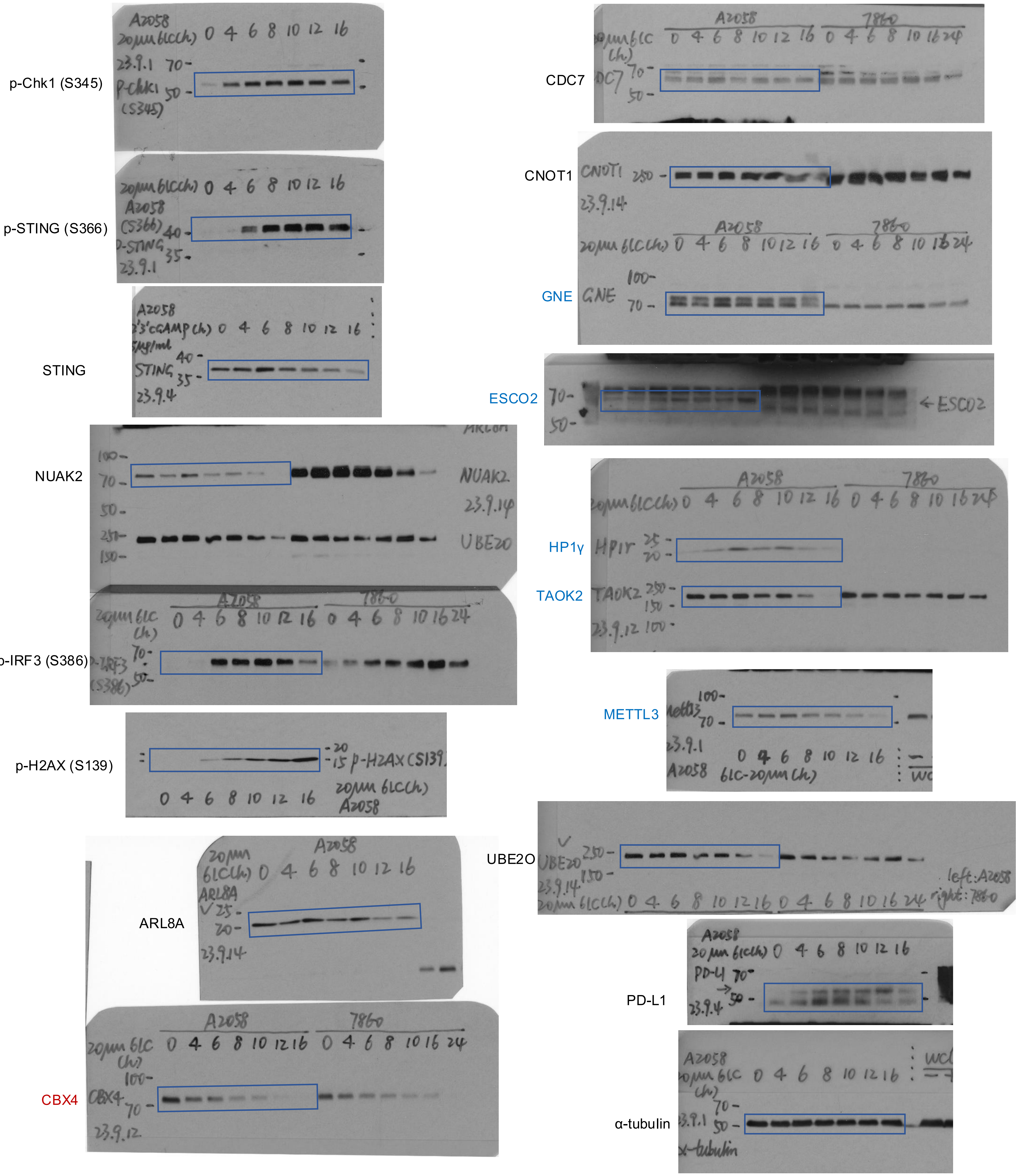

Full unedited blot for Figure 10B

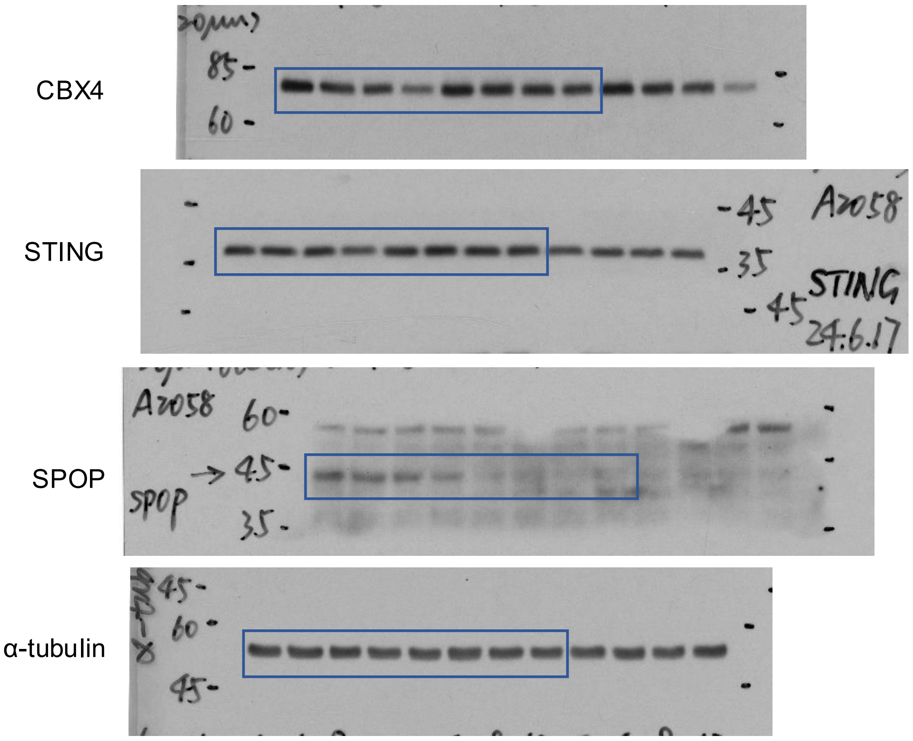

Full unedited blot for Figure 10C

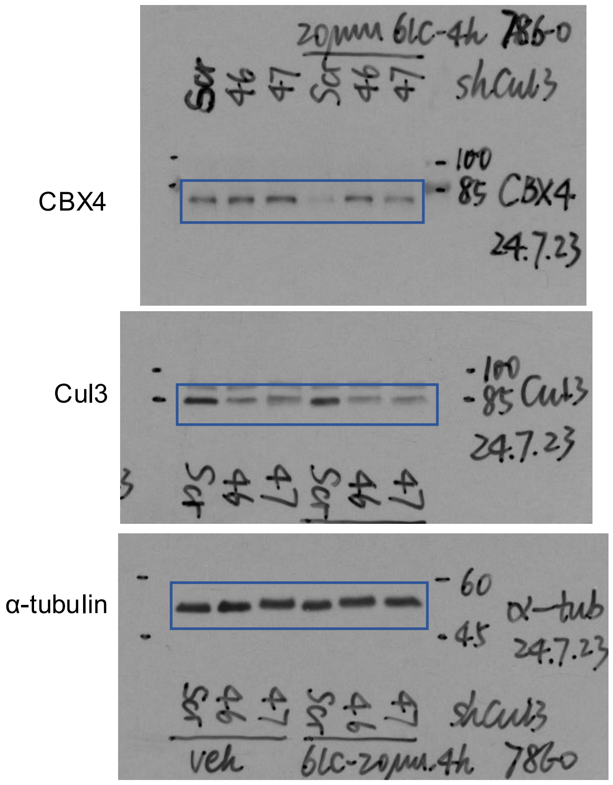

Full unedited blot for Figure 10D

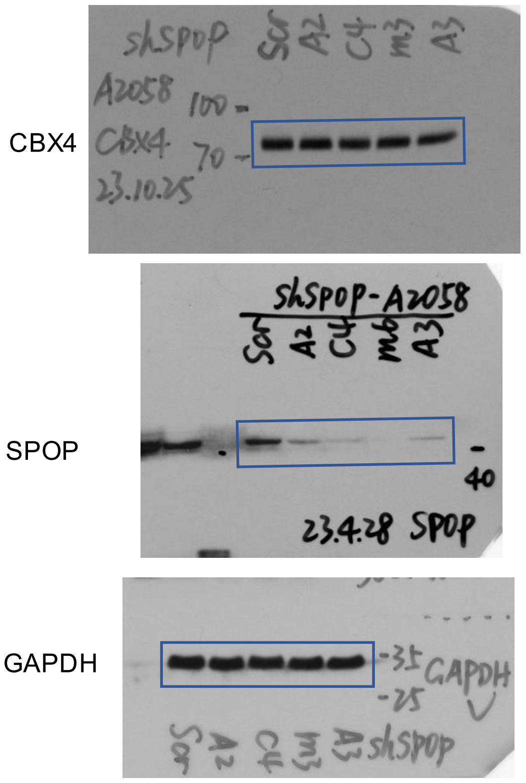

Full unedited blot for Figure 10F

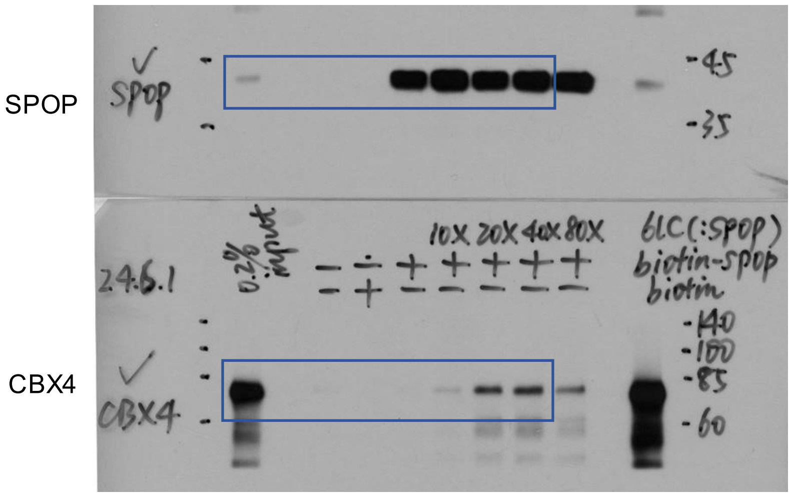

Full unedited blot for Figure 10H

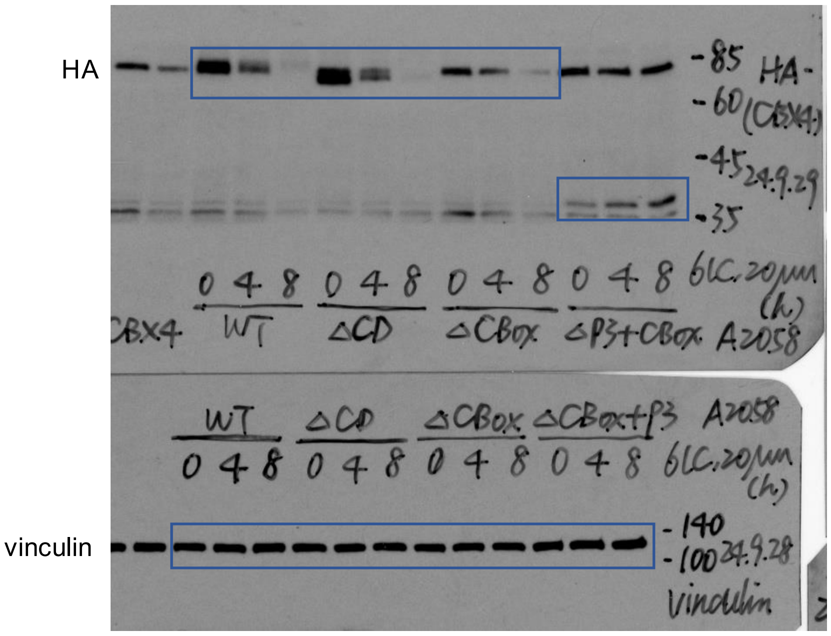

Figure 11

Full unedited blot for Figure 11A and 11B

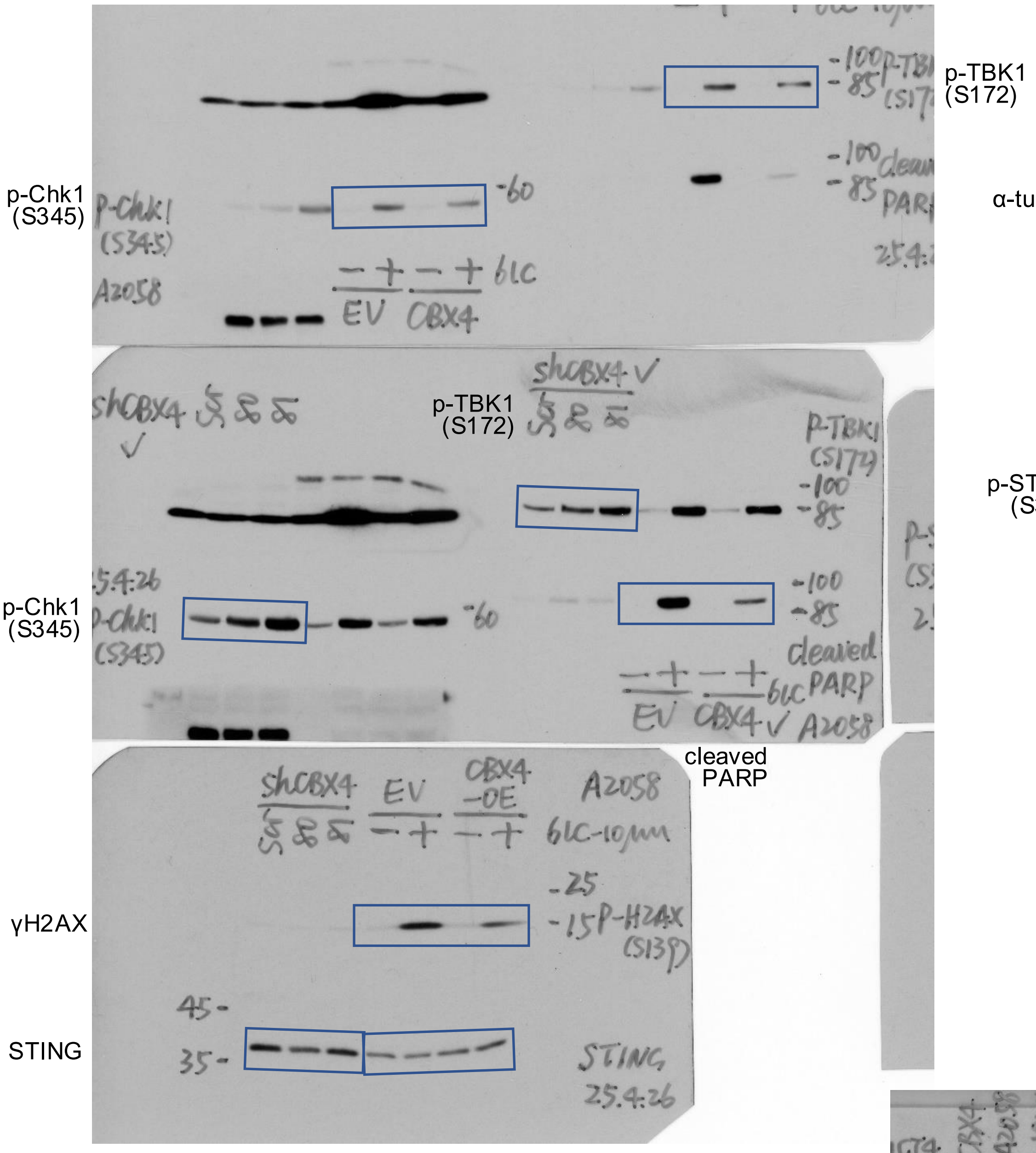

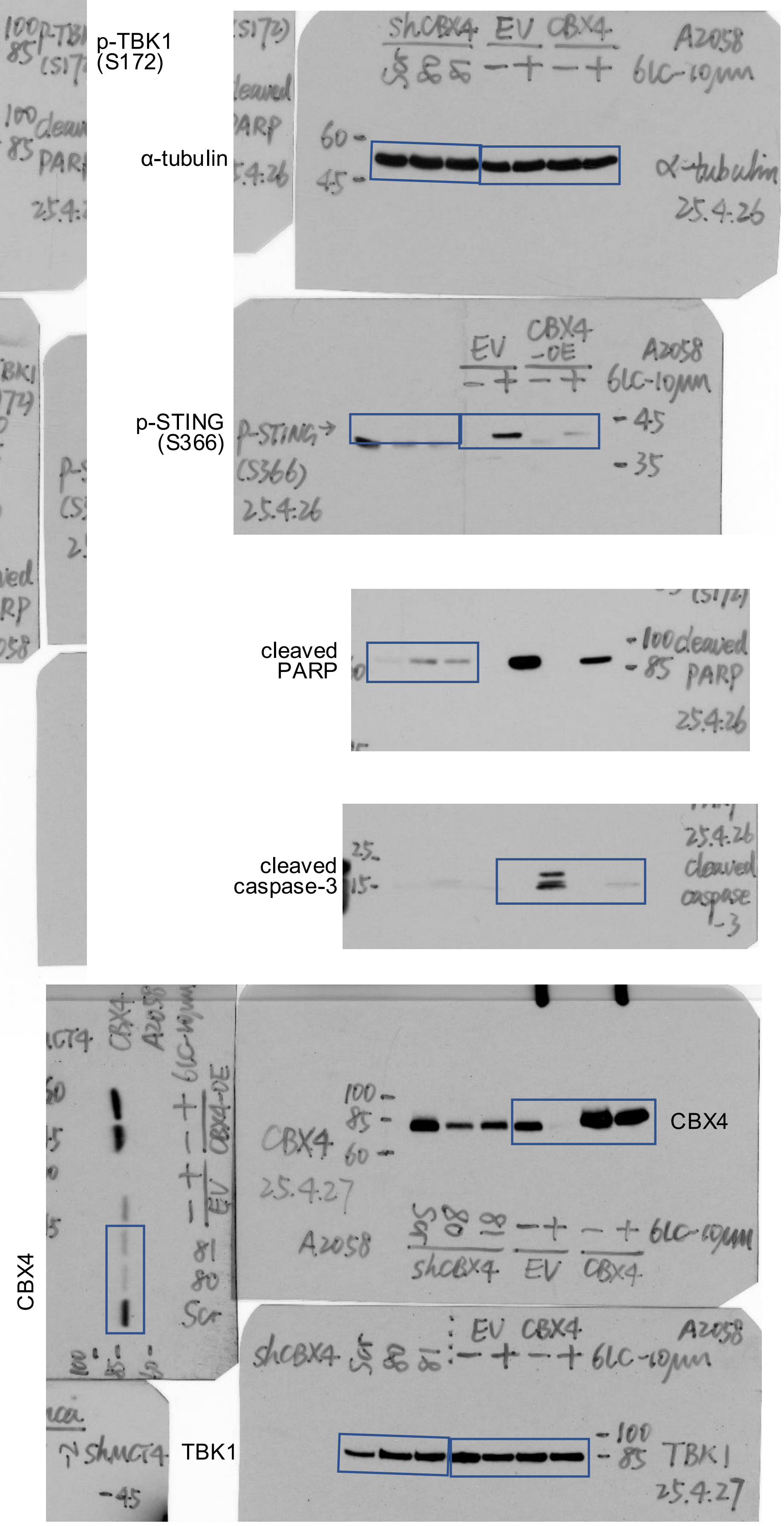

I run 7 samples (lane 1-3 were shScr and shCBX4-1/2 in Fig 6A; lane 4-7 were EV and CBX4-OE with or without 6lc treatment in Fig 6B) in several gels, cut membranes into multiple pieces and detected proteins as indicated on 04/26/2025 and 04/27/2025.

Full unedited blot for Figure 11K

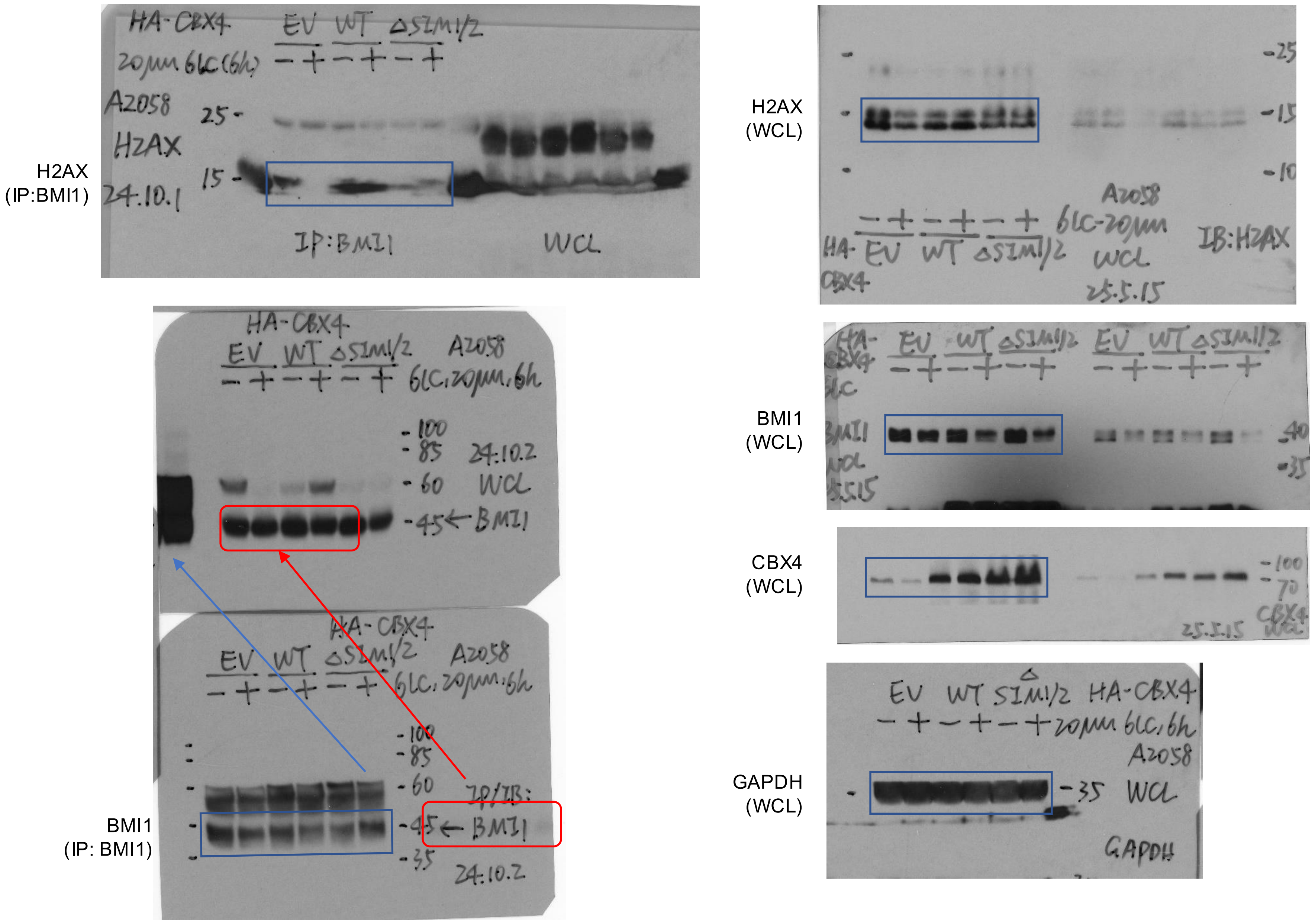

IP and WCL samples were prepared and analyzed in 10/2024. H2AX, BMI1 and CBX4 in WCL were detected again on 05/15/2025 per reviewer's request.

Figure S1

Full unedited blot for Figure S1K

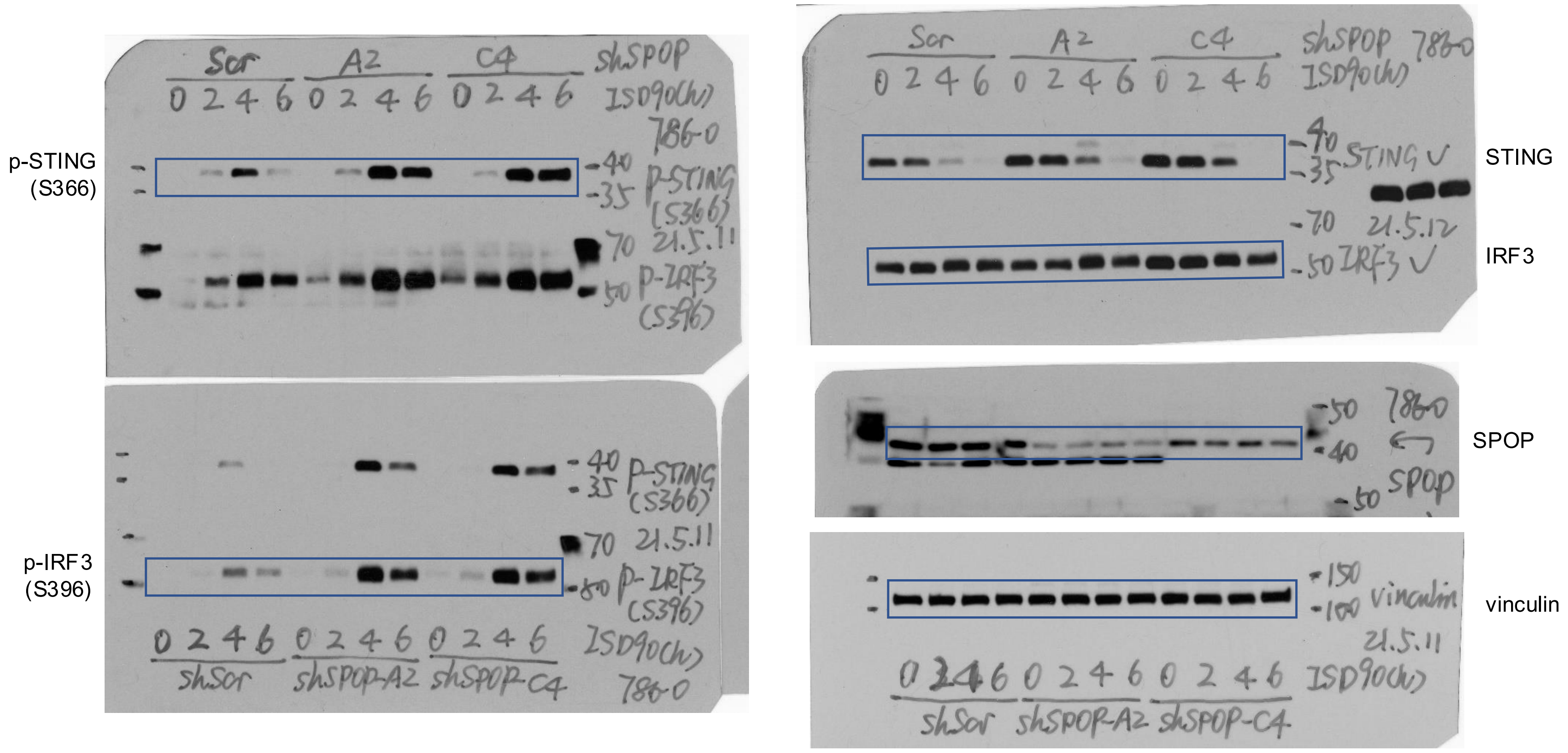

Full unedited blot for Figure S1Z

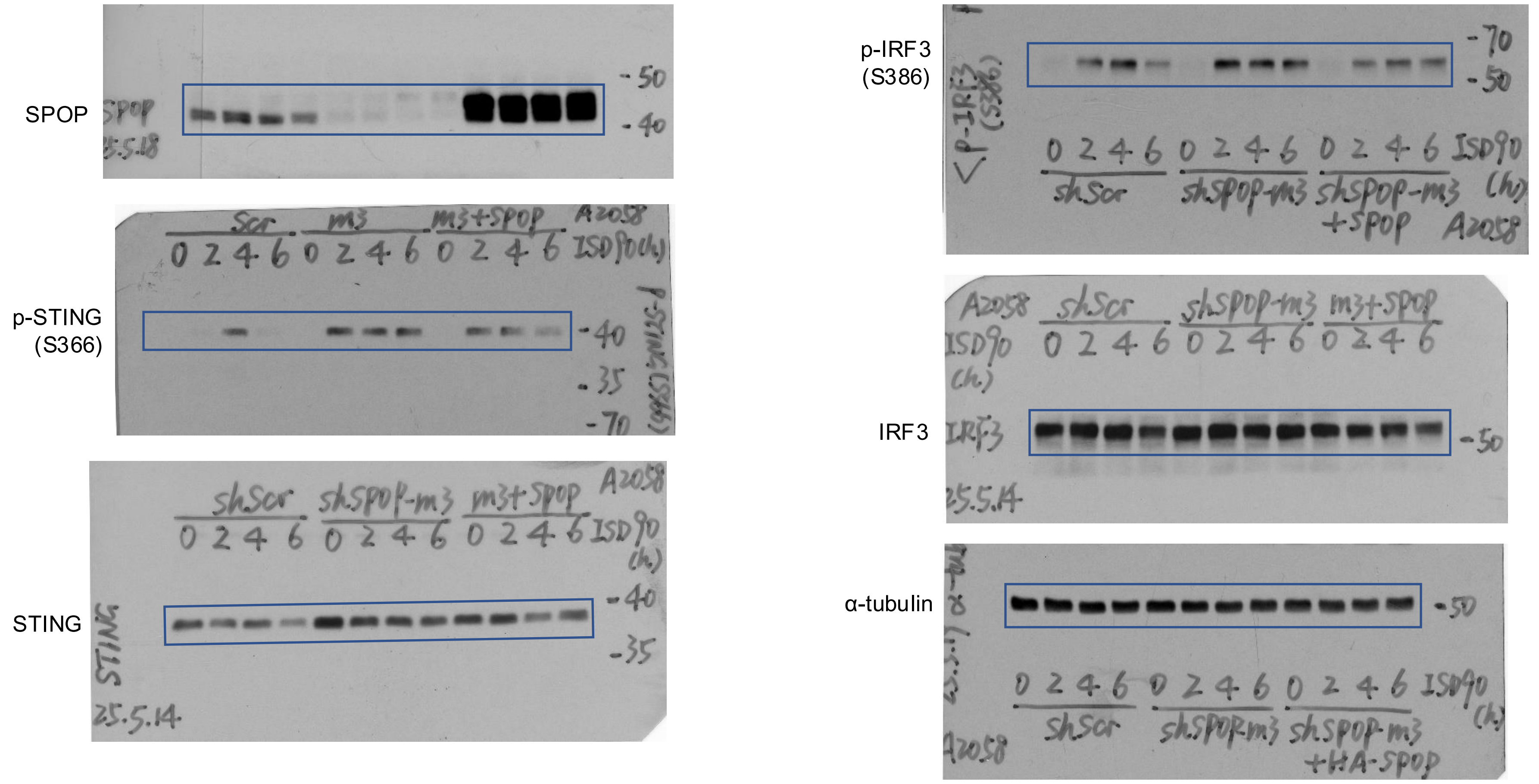

Full unedited blot for Figure S1Z1

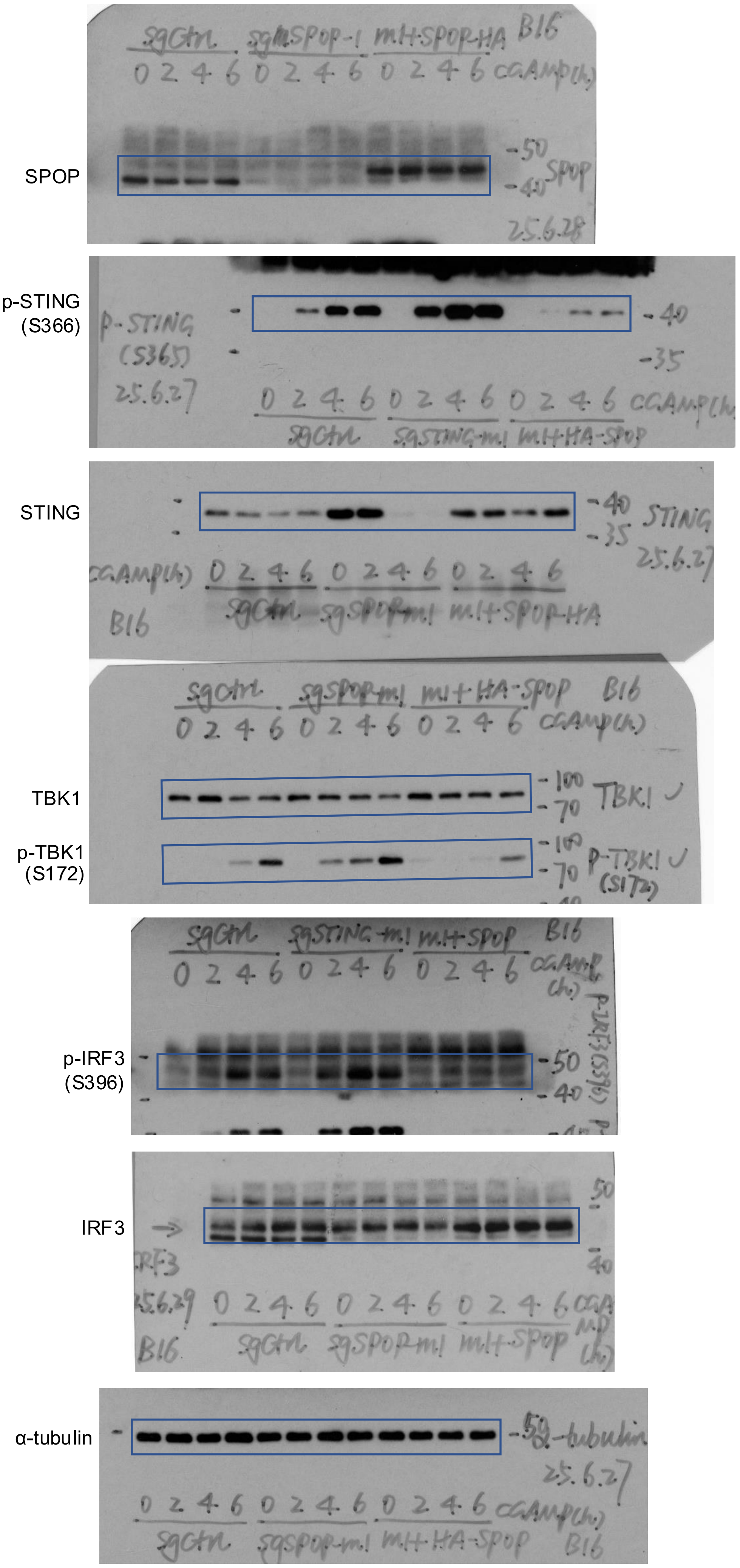

Figure S2

Full unedited blot for Figure S2A and 2F

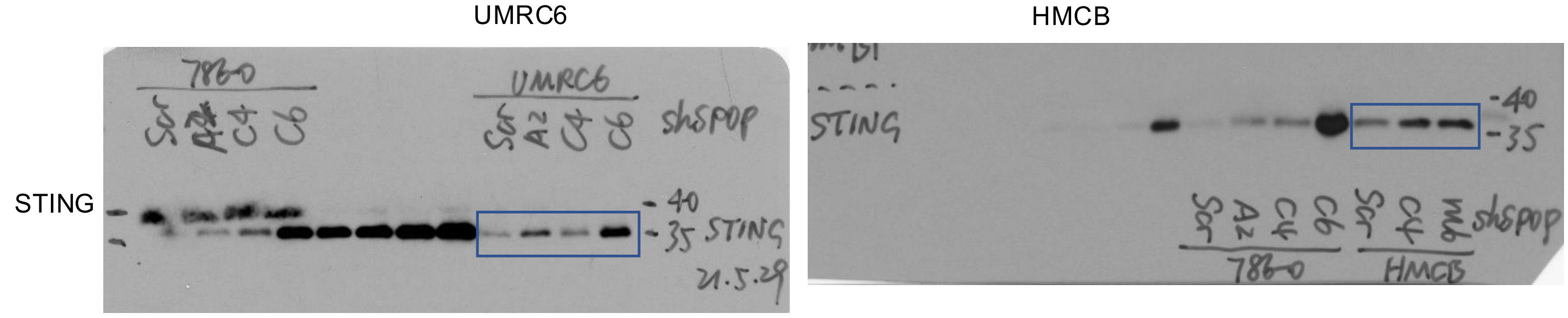

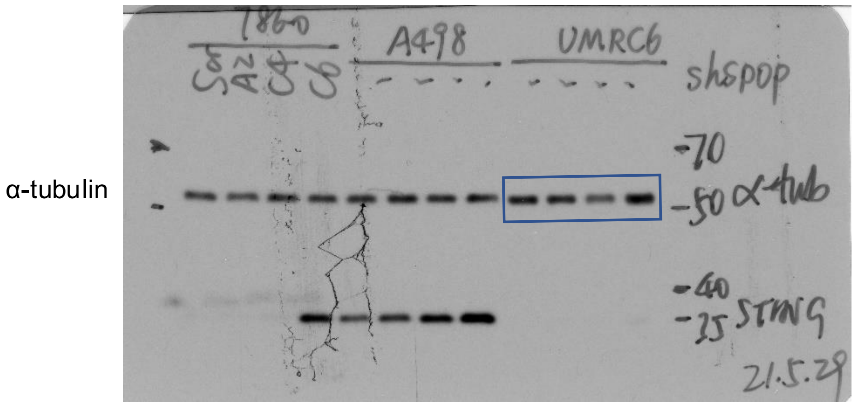

In Fig 2F, STING and α-tubulin were detected on 05/29/2021; other proteins were detected on 09/29-10/01/2022, with membrane stripped.

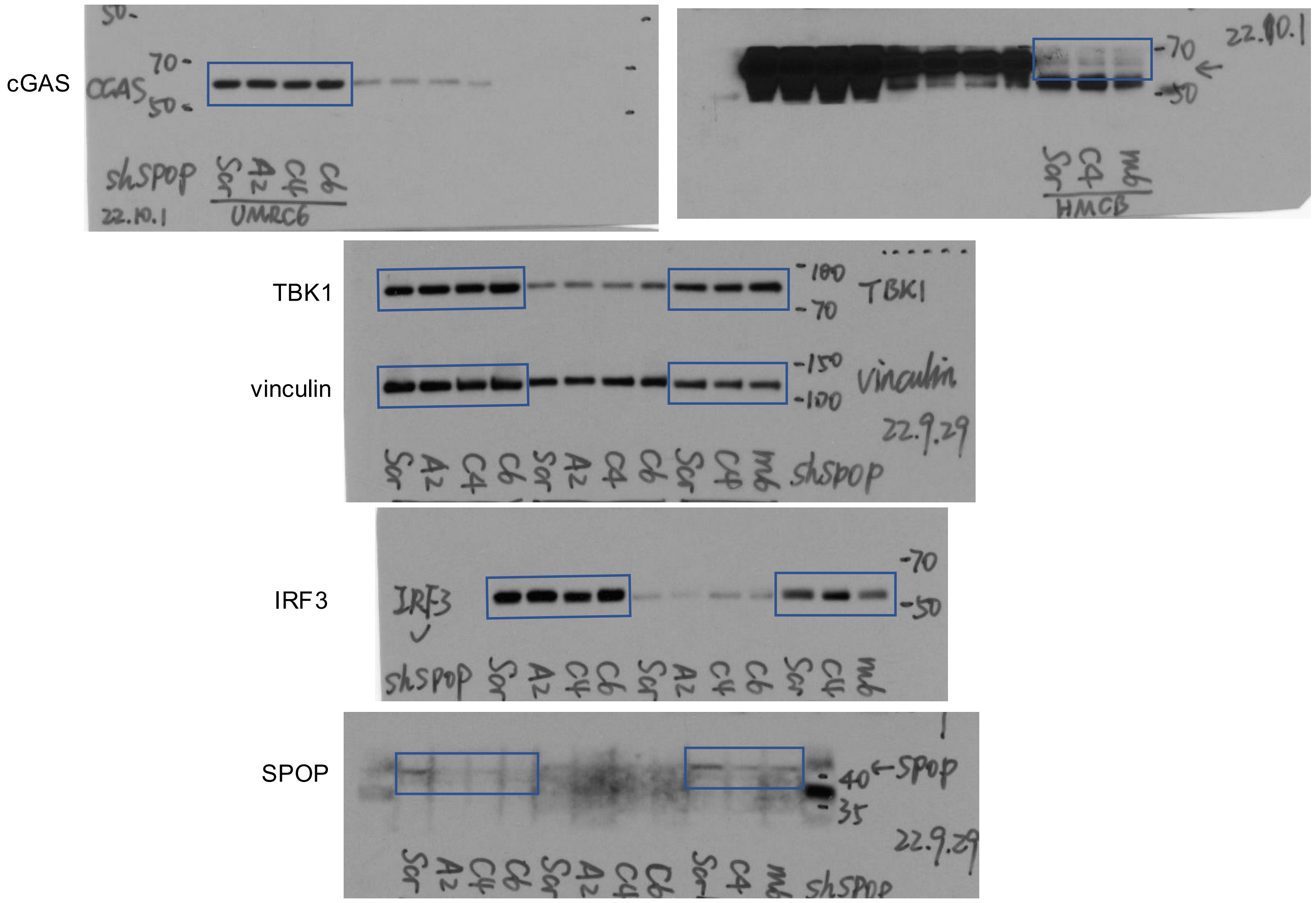

Full unedited blot for Figure S2B

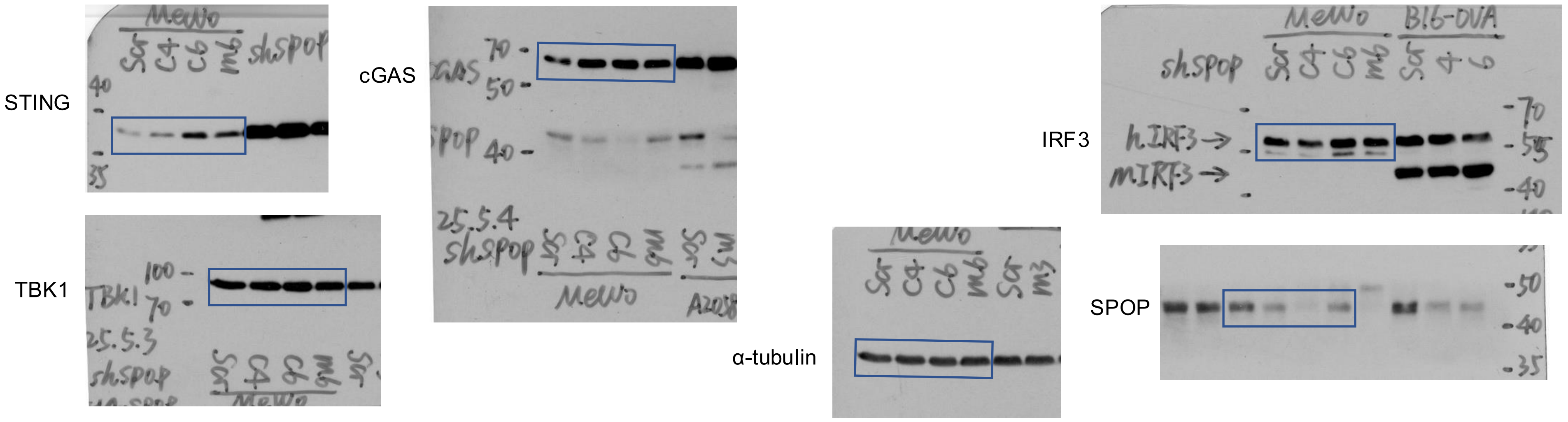

Full unedited blot for Figure S2C

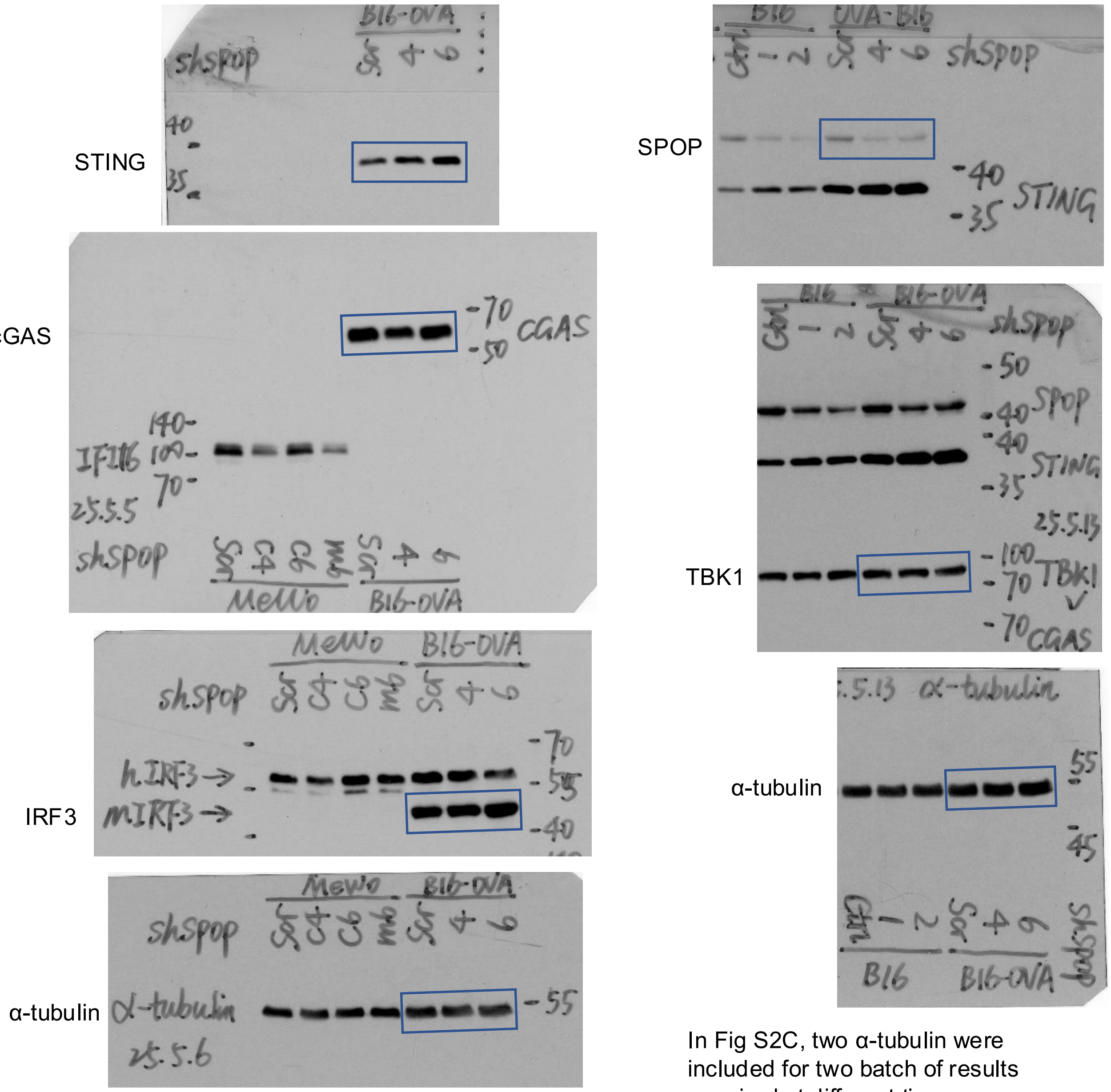

In Fig S2C, two α-tubulin were included for two batch of results acquired at different time.

Full unedited blot for Figure S2D

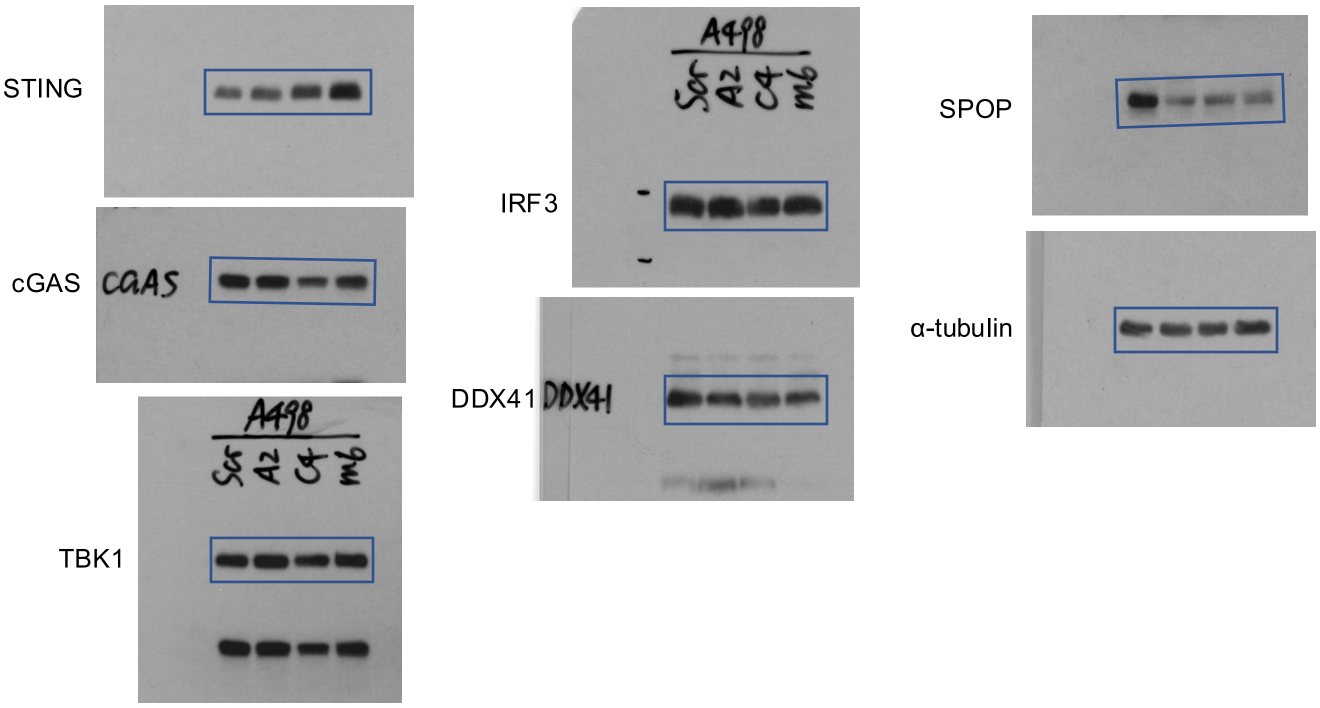

Full unedited blot for Figure S2E

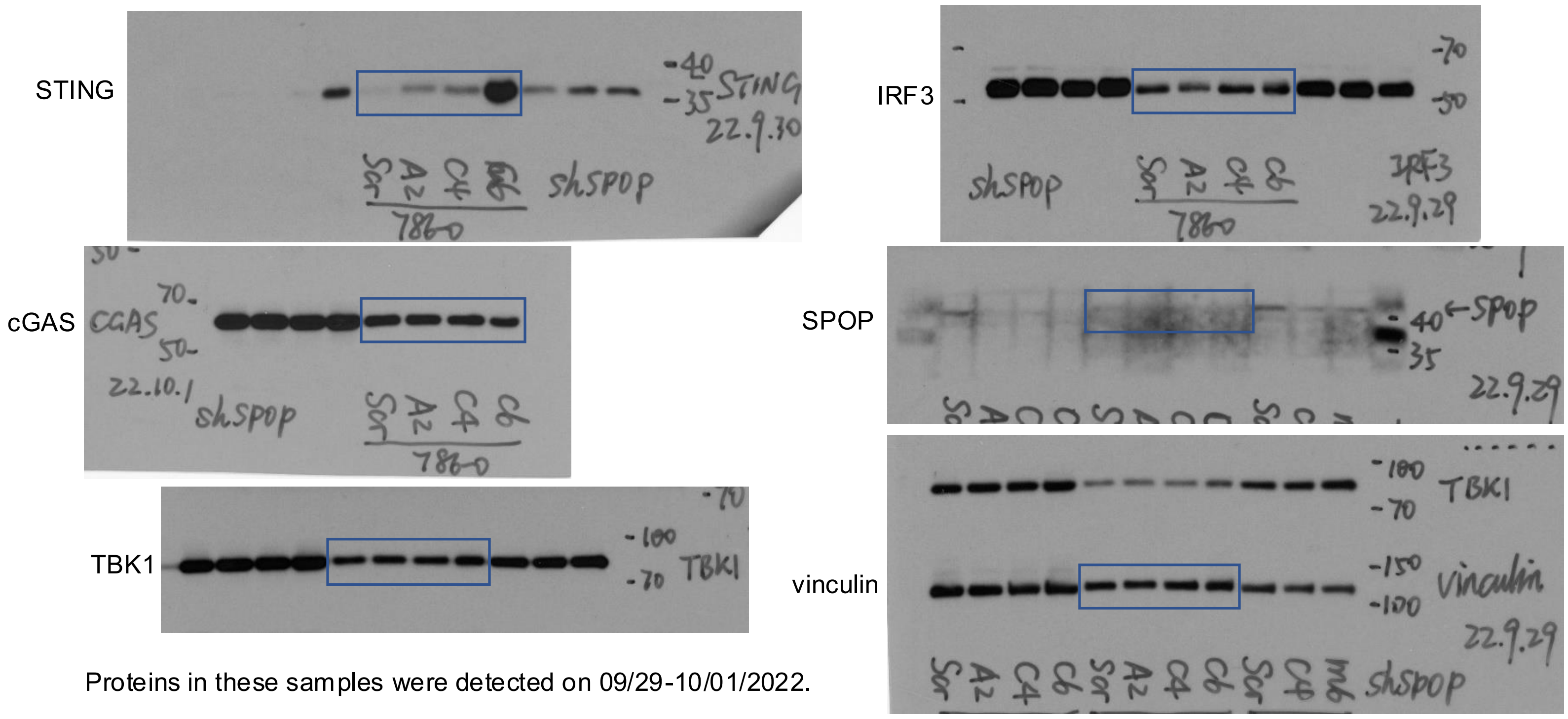

Proteins in these samples were detected on 09/29-10/01/2022.

Full unedited blot for Figure S2G

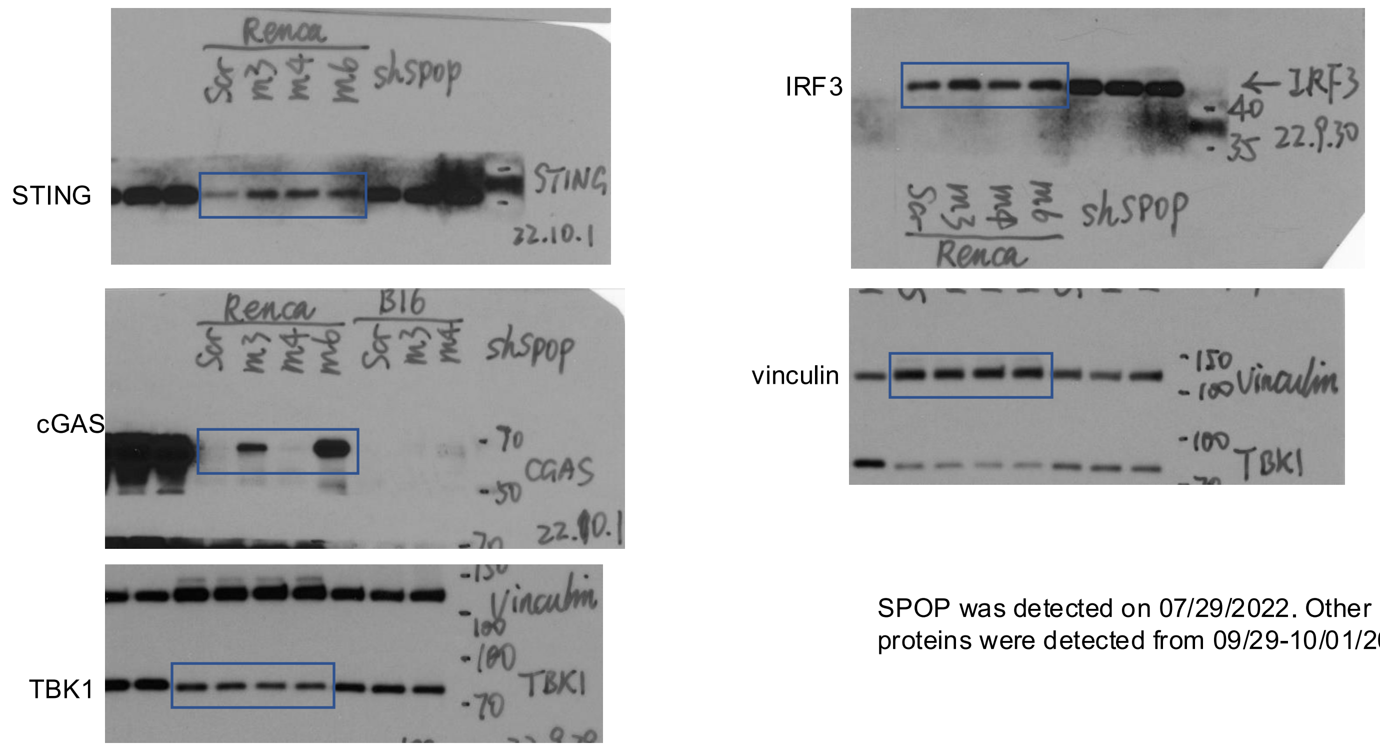

SPOP was detected on 07/29/2022. Other proteins were detected from 09/29-10/01/2022.

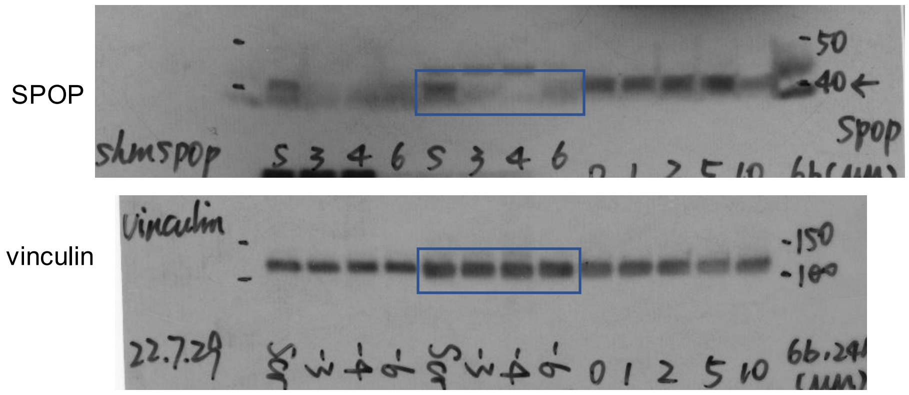

Full unedited blot for Figure S2H

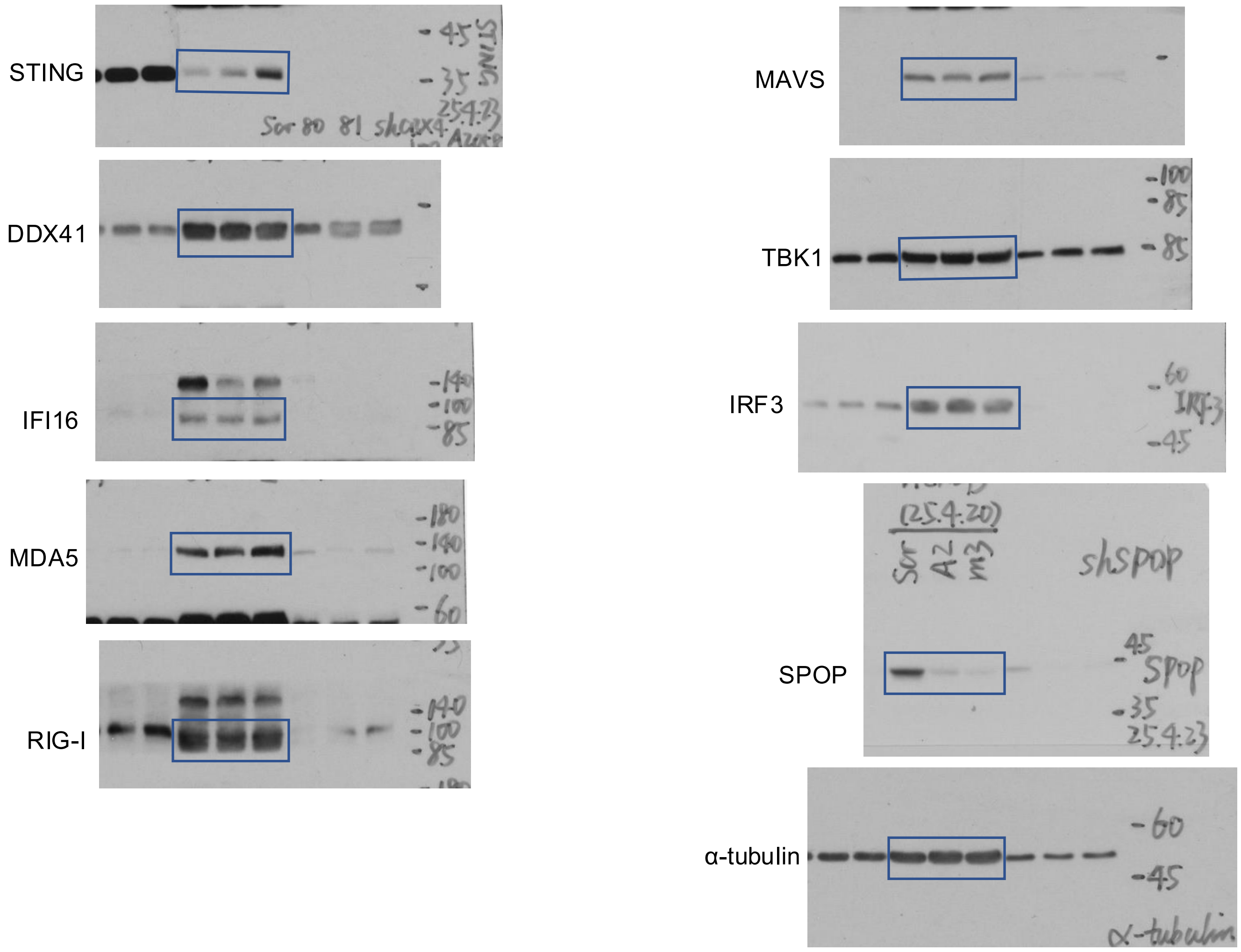

Full unedited blot for Figure S2J

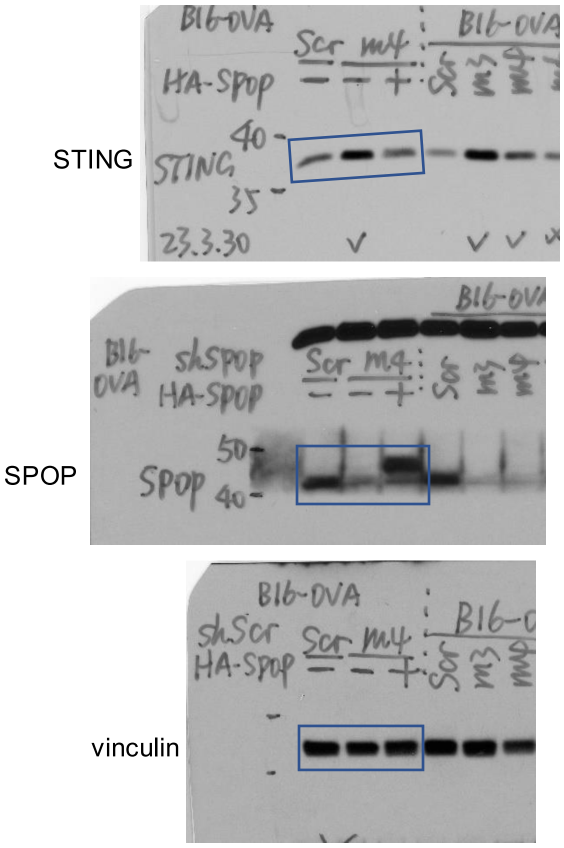

Full unedited blot for Figure S2K

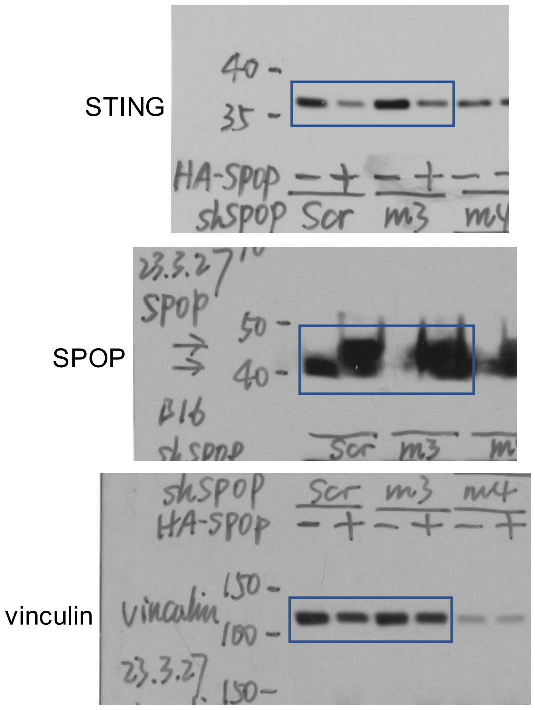

Full unedited blot for Figure S2L

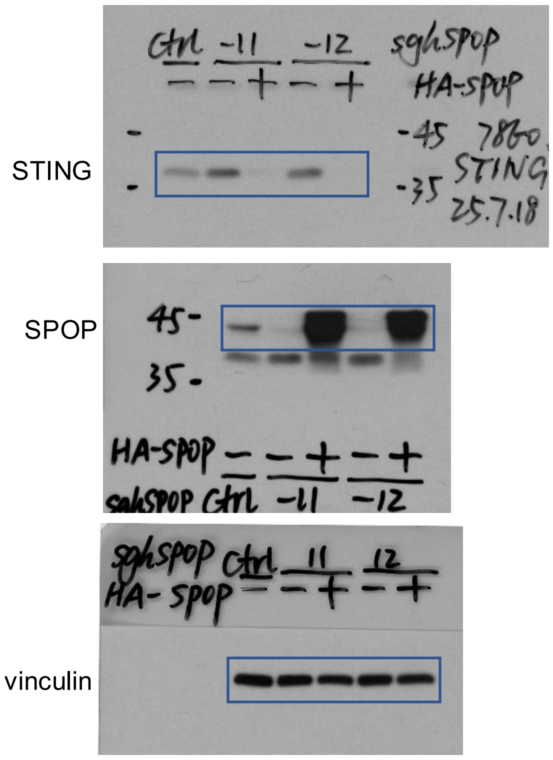

Full unedited blot for Figure S2M

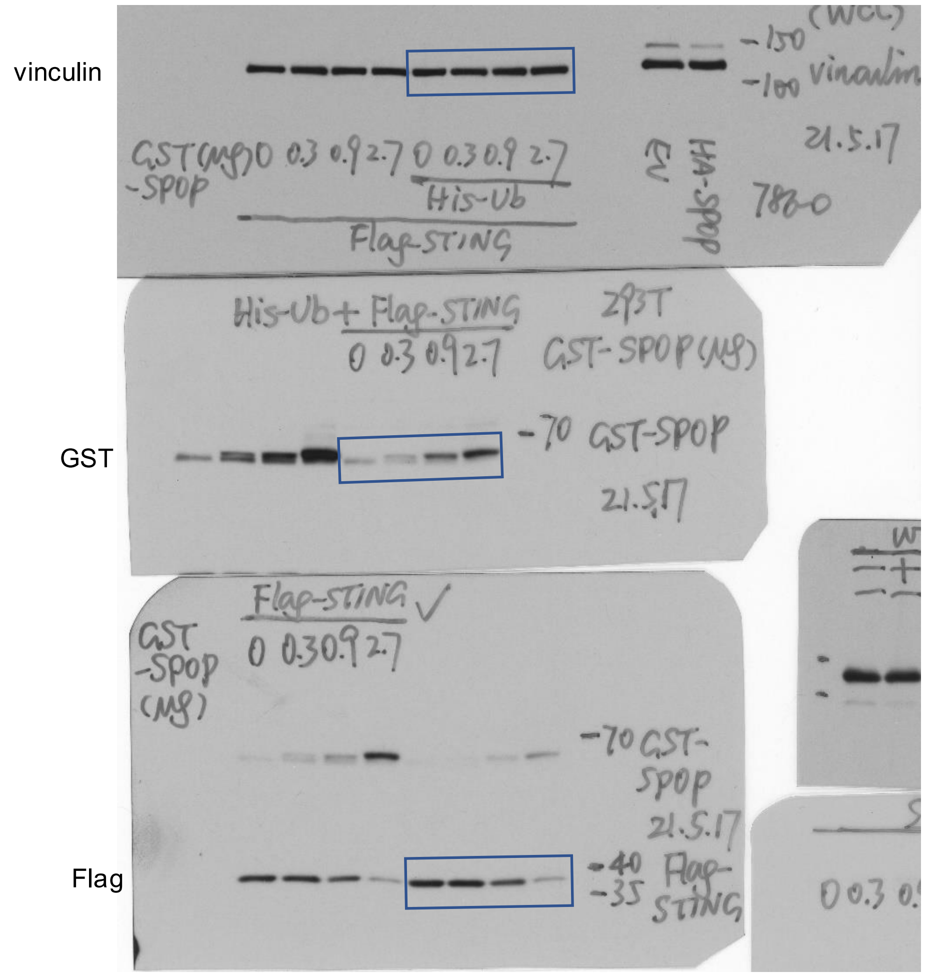

Full unedited blot for Figure S2N

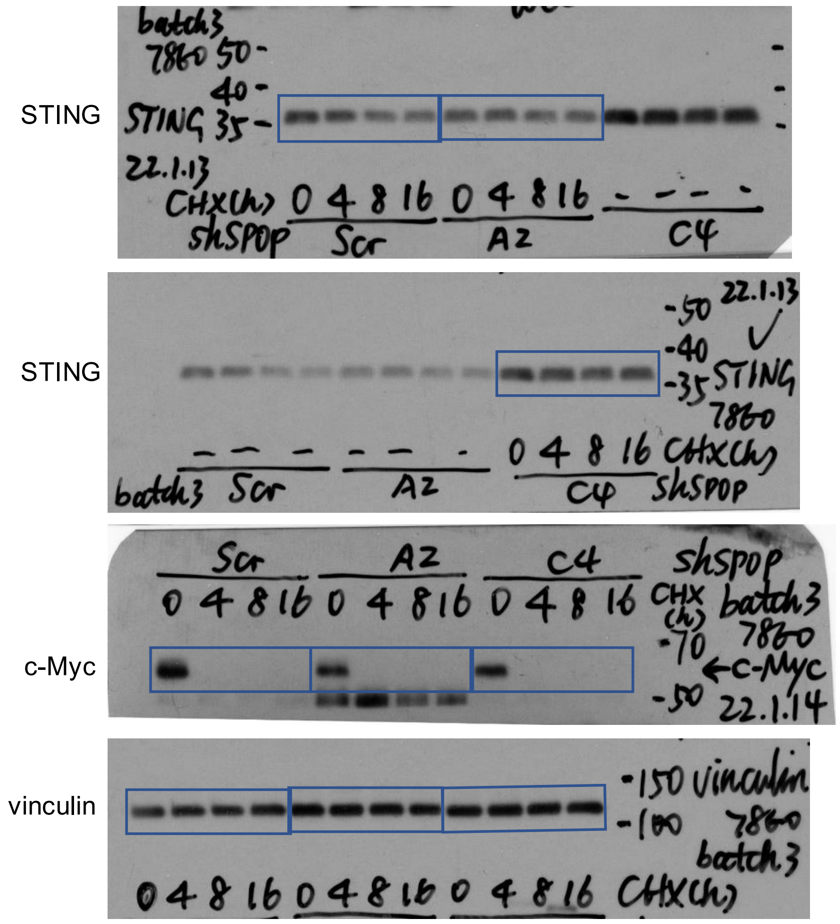

Full unedited blot for Figure S2P

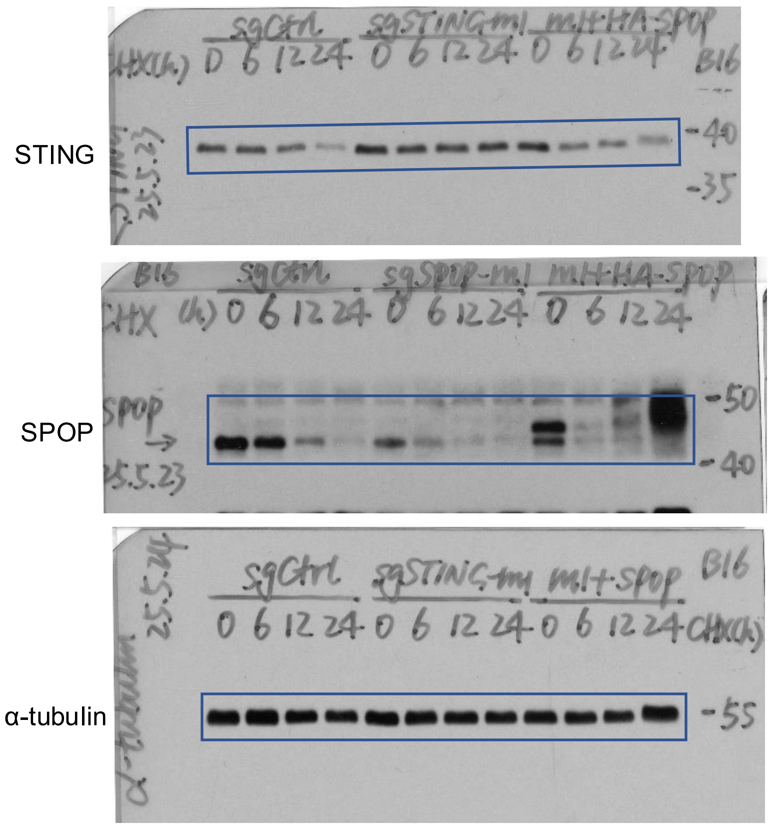

Full unedited blot for Figure S2R

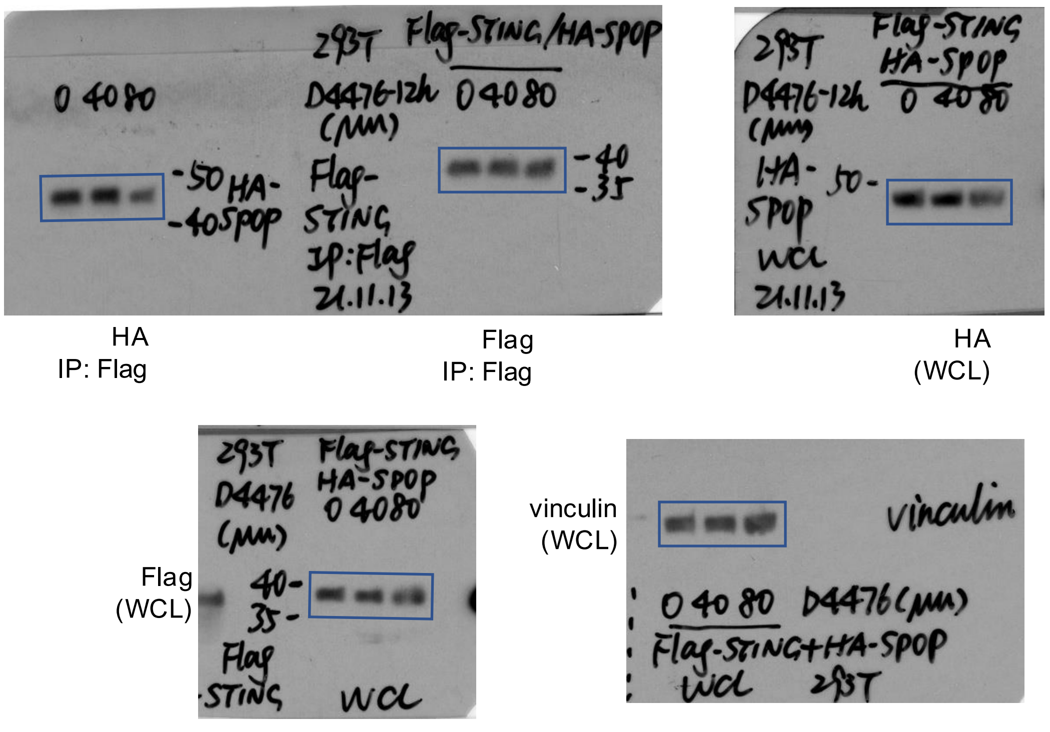

Figure S3

Full unedited blot for Figure S3E

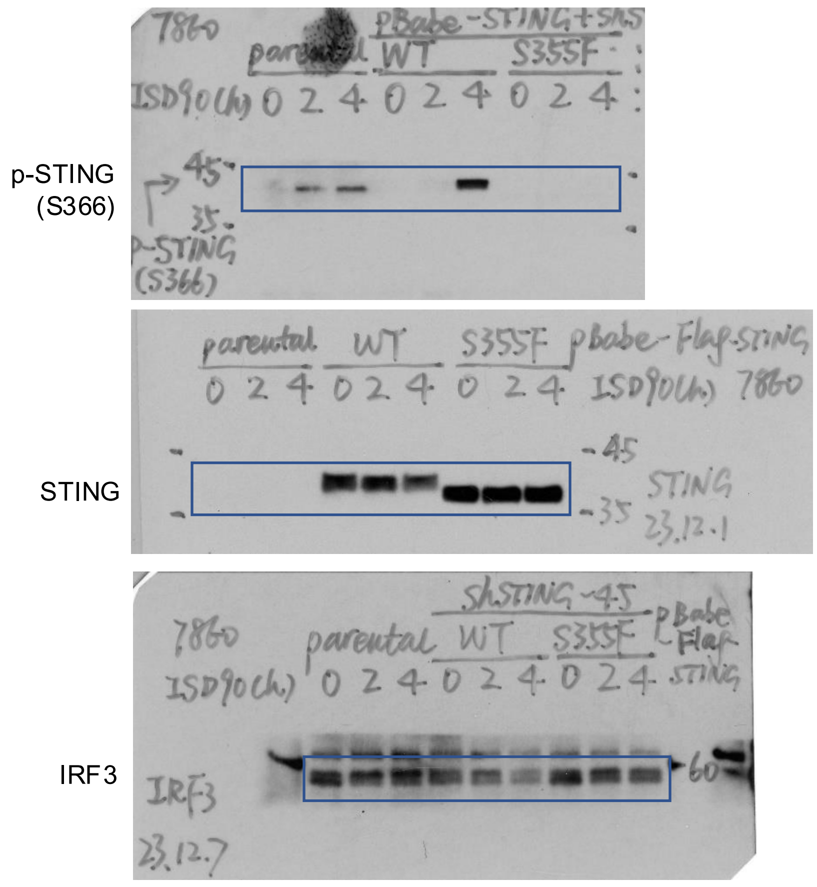

Full unedited blot for Figure S3F

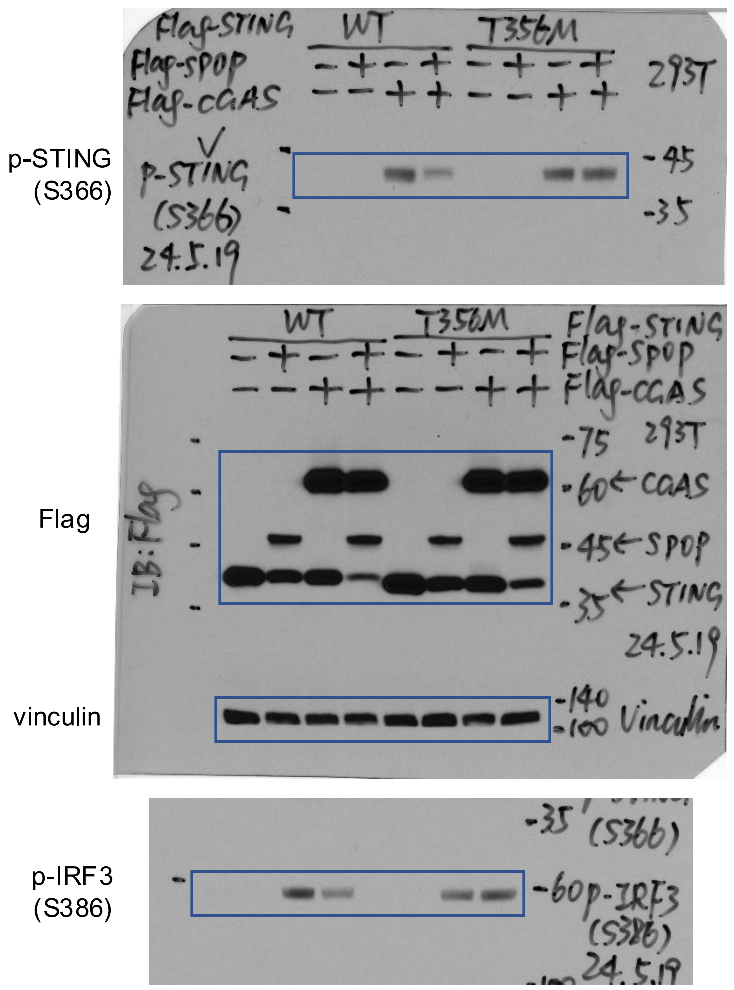

Full unedited blot for Figure S3G

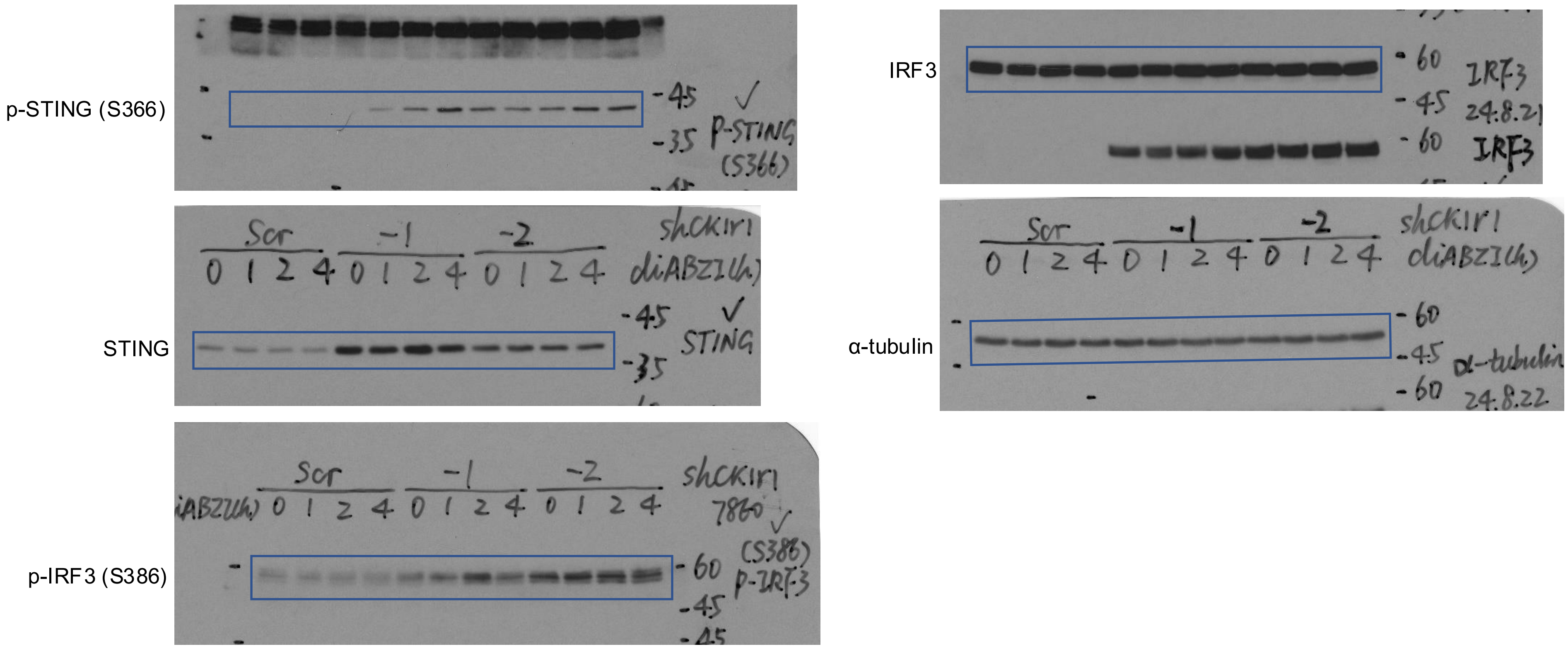

Full unedited blot for Figure S3H

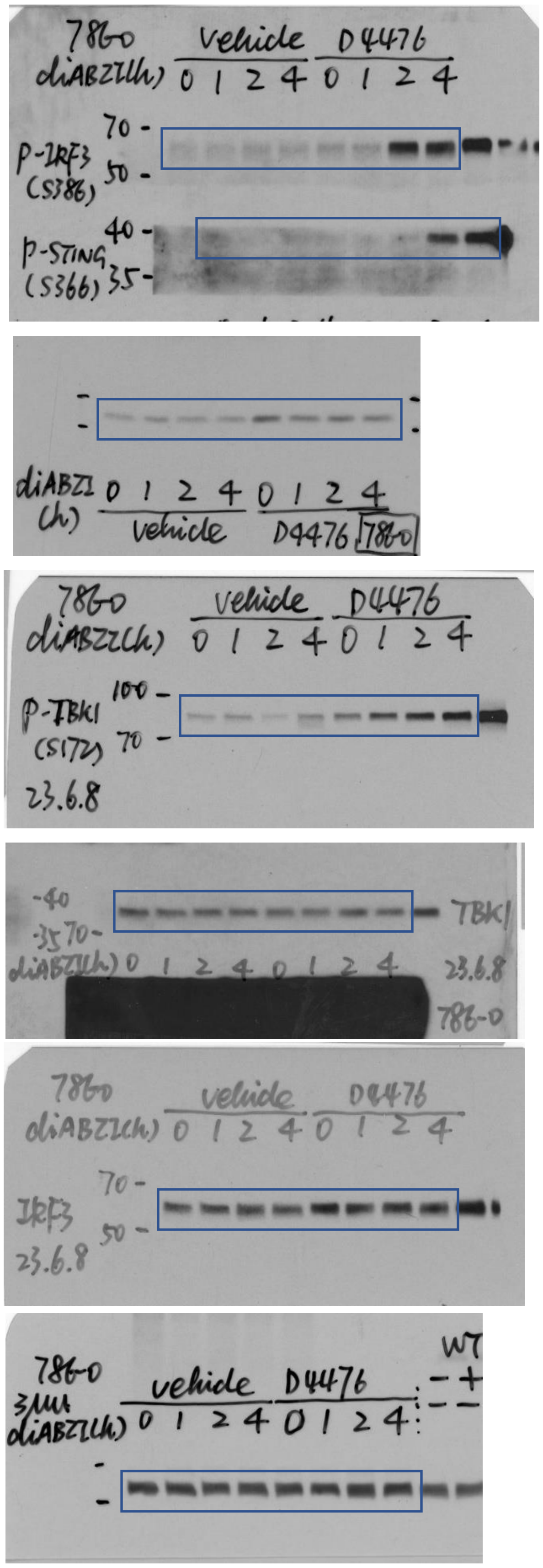

p-IRF3 (S386)

p-STING (S366)

STING

p-TBK1 (S172)

TBK1

IRF3

vinculin

Figure S4

Full unedited blot for Figure S4A

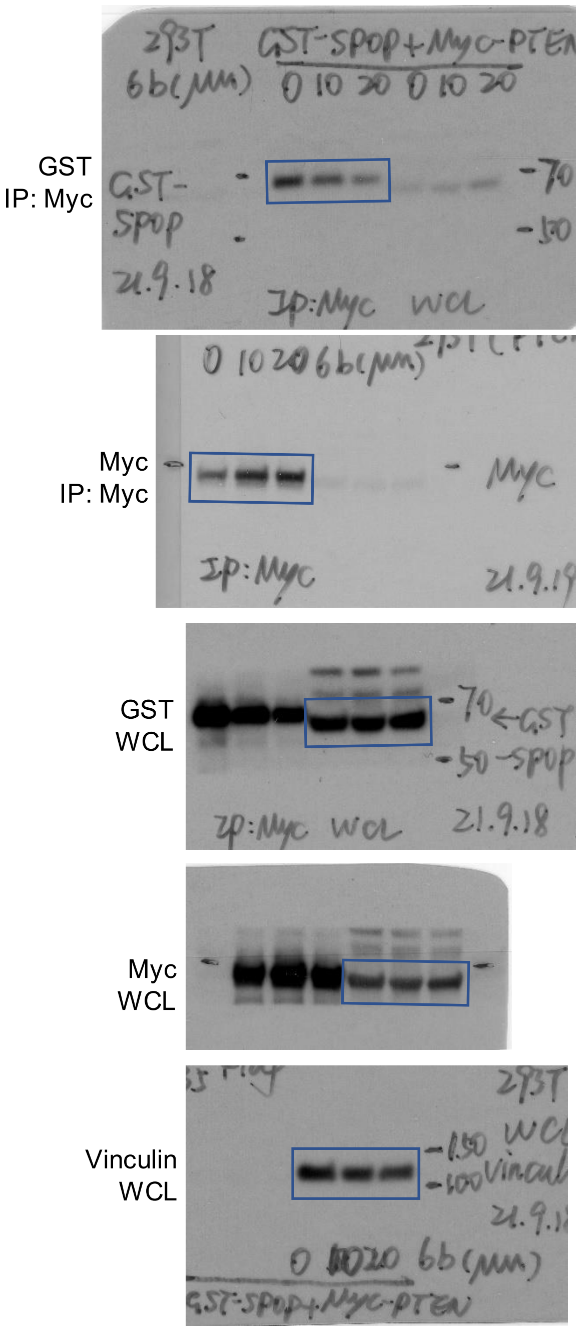

Full unedited blot for Figure S4B

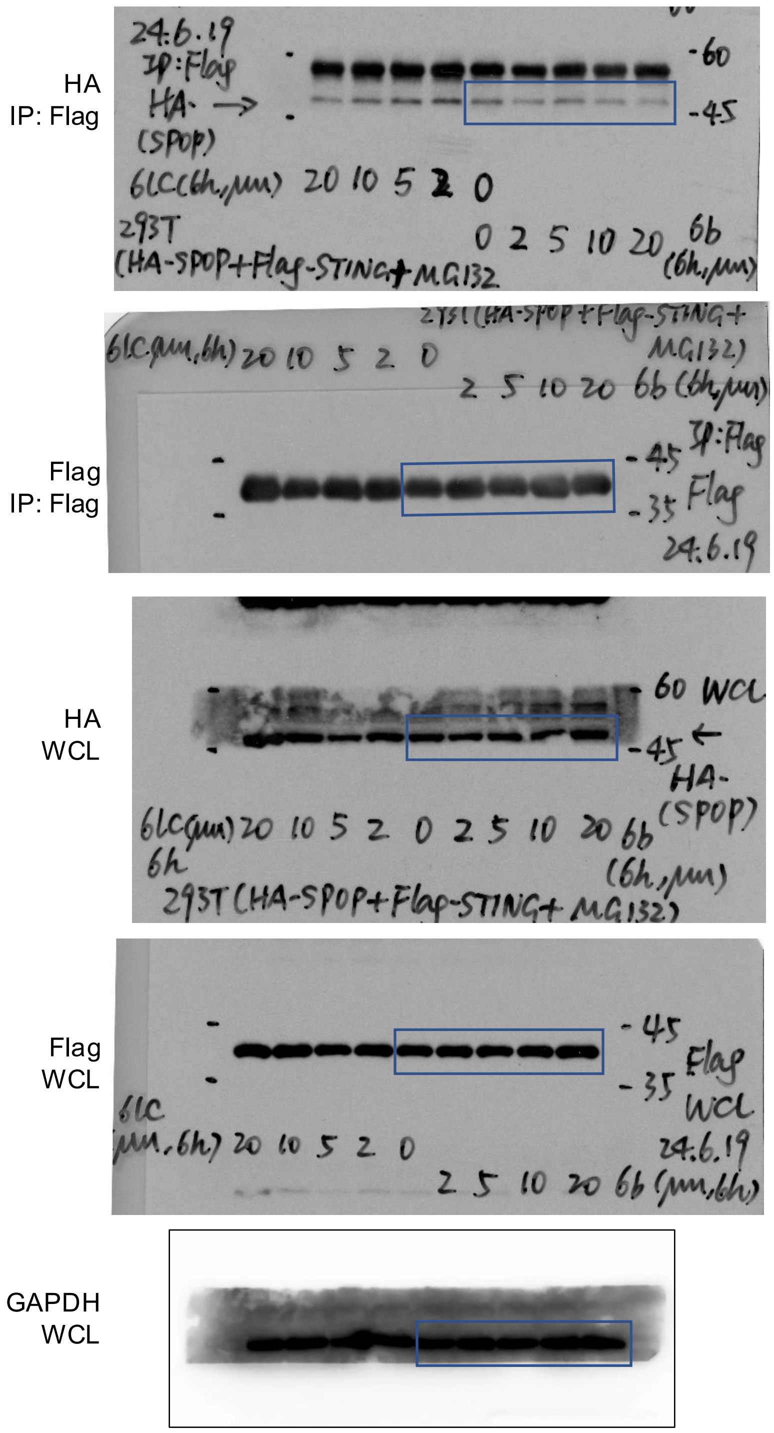

Full unedited blot for Figure S4C

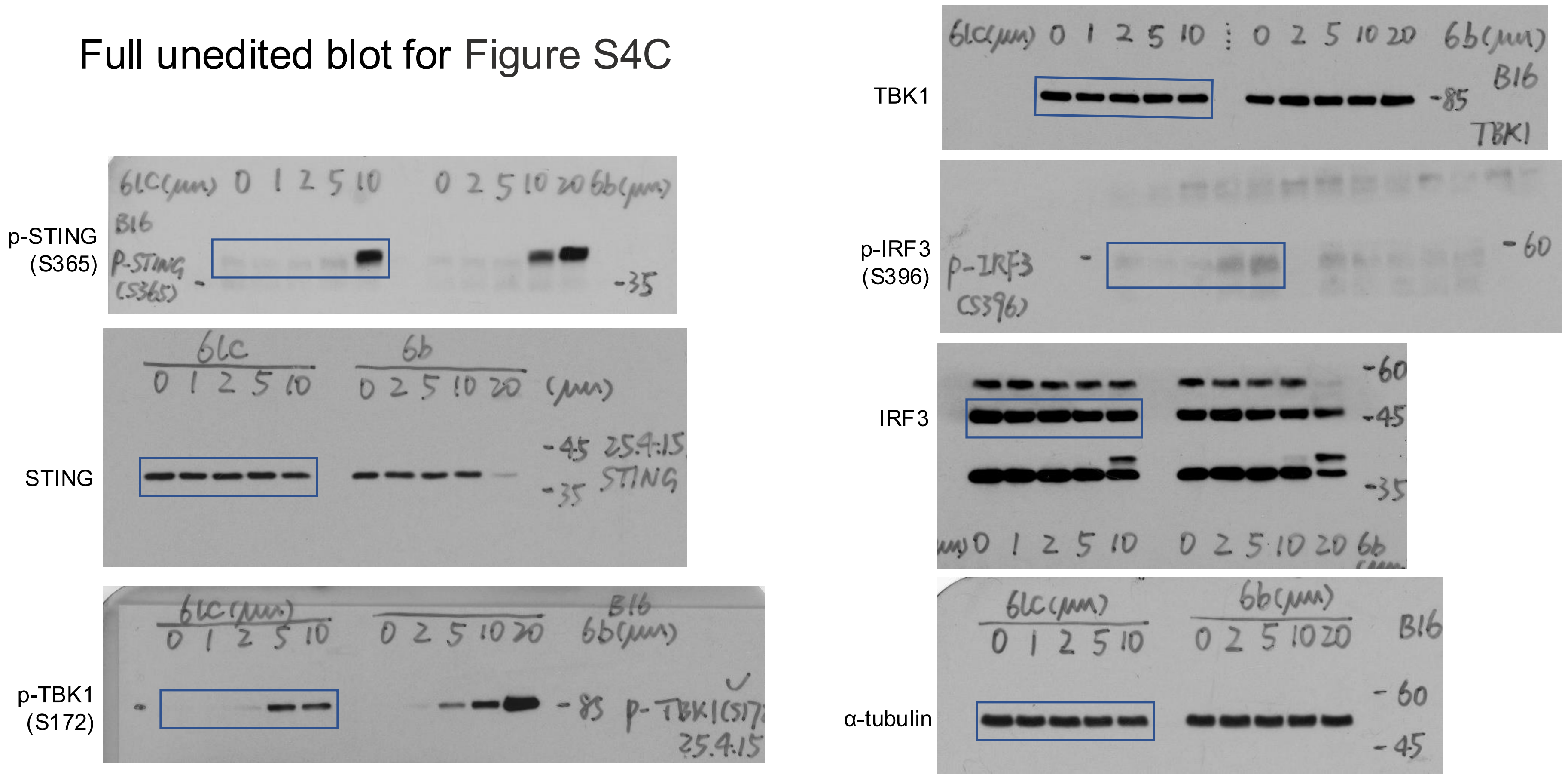

Full unedited blot for Figure S4D

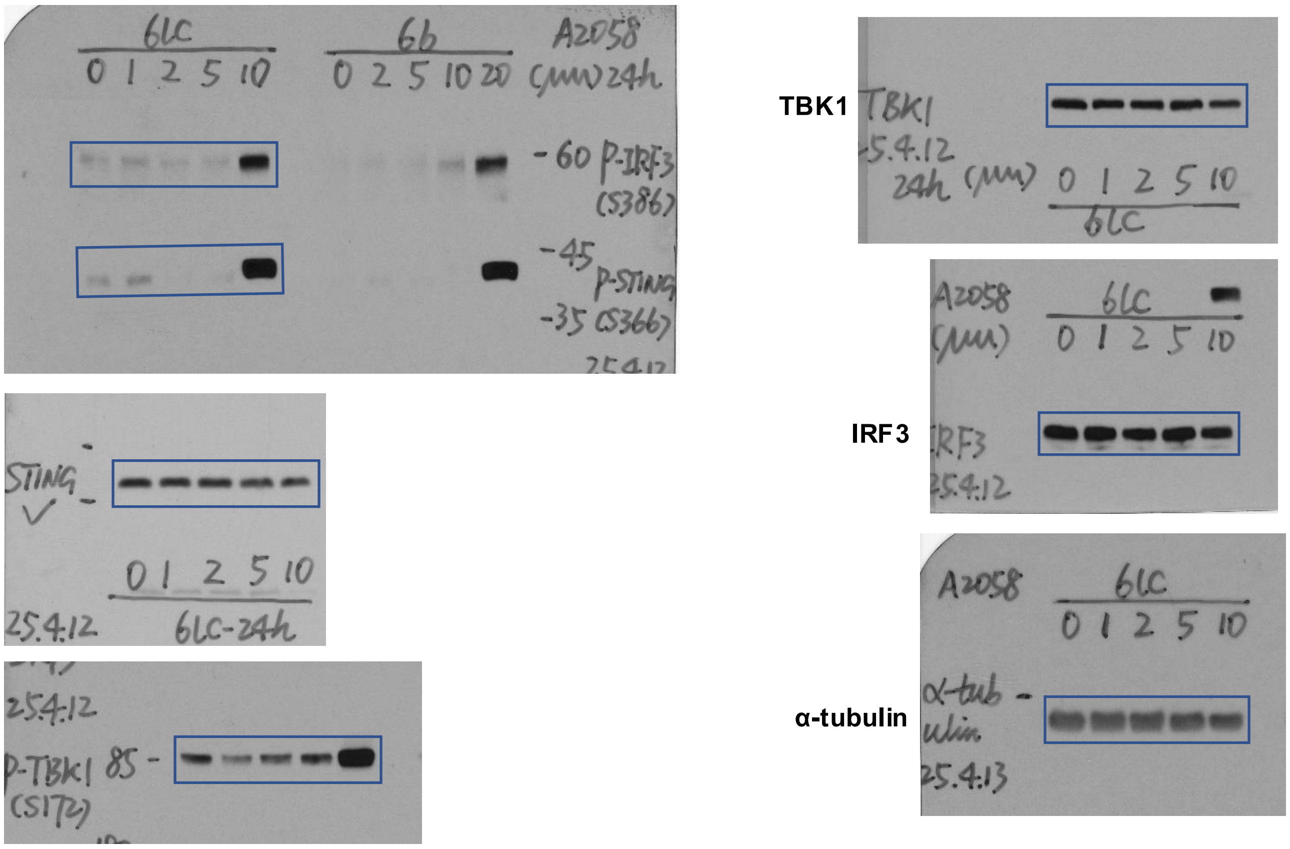

p-IRF3 (S386)

p-STING (S366)

STING

p-TBK1 (S172)

TBK1

IRF3

α-tubulin

Full unedited blot for Figure S4E

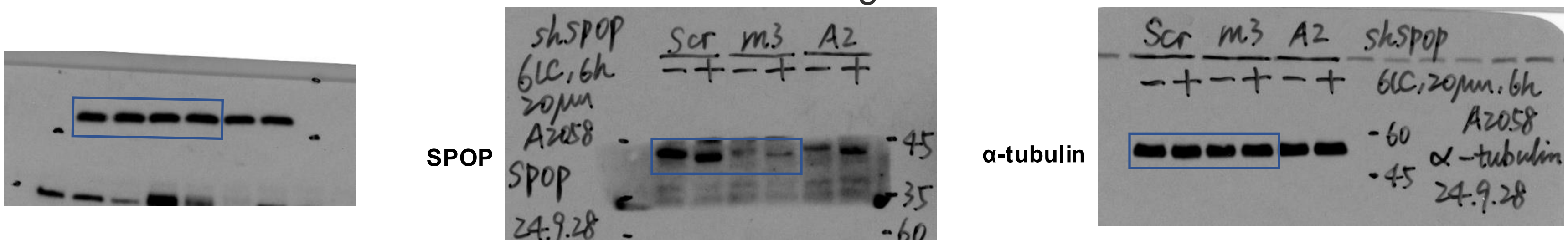

STING

SPOP

α-tubulin

Full unedited blot for Figure S4F

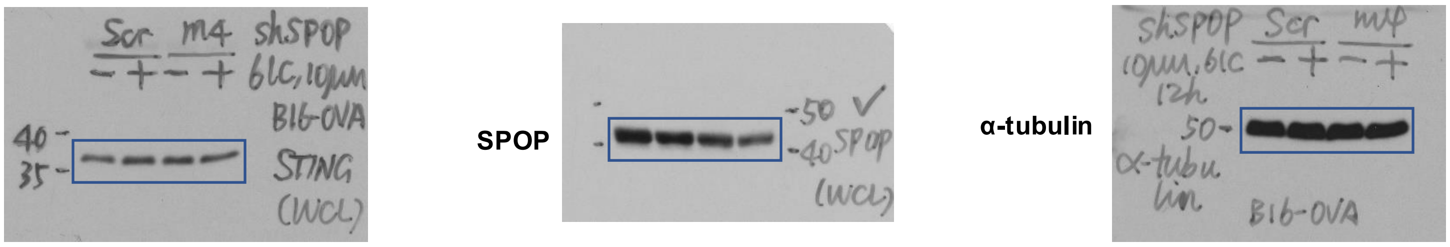

STING

SPOP

α-tubulin

Full unedited blot for Figure S4L

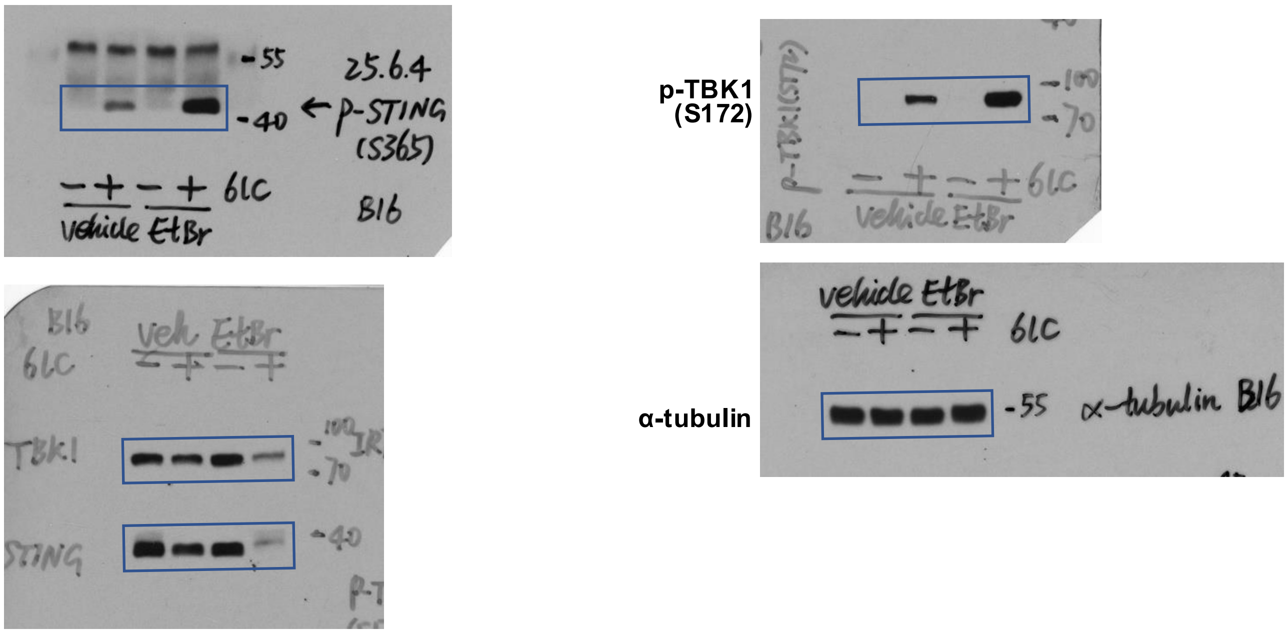

p-STING (S365)

p-TBK1 (S172)

TBK1

STING

α-tubulin

Full unedited blot for Figure S4P

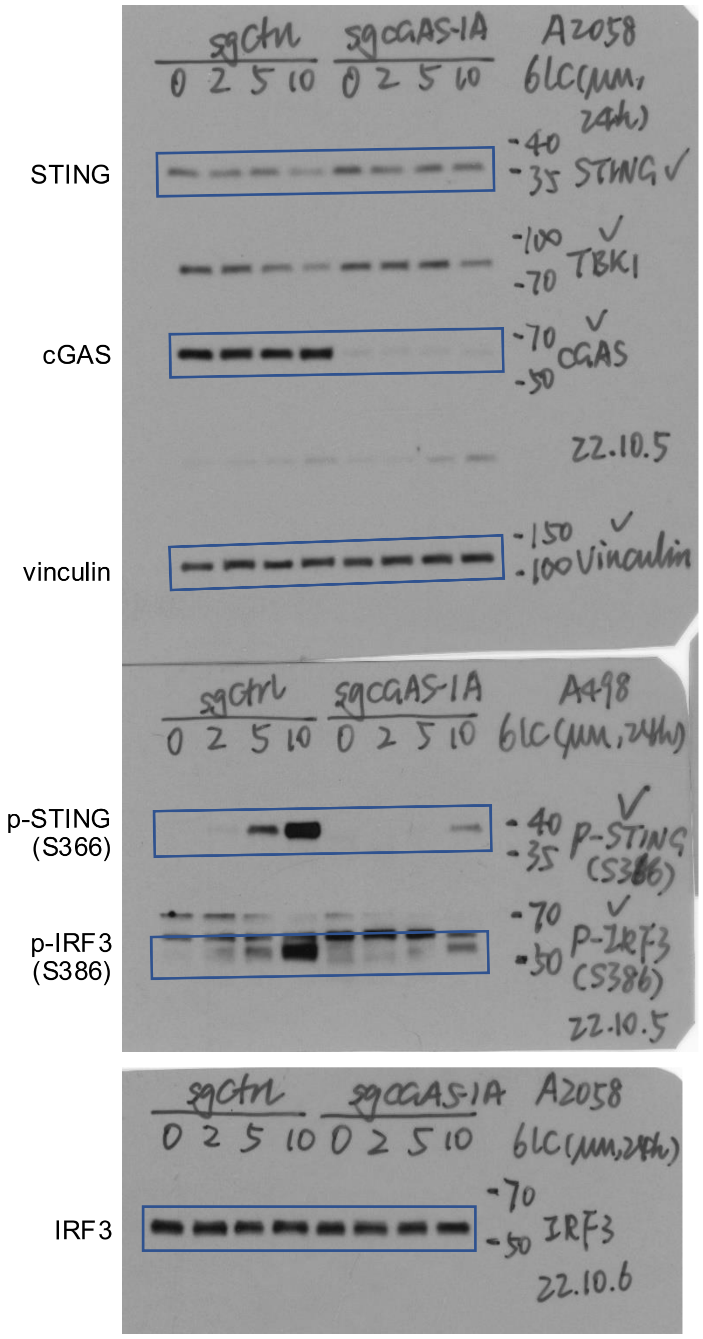

Full unedited blot for Figure S4Q

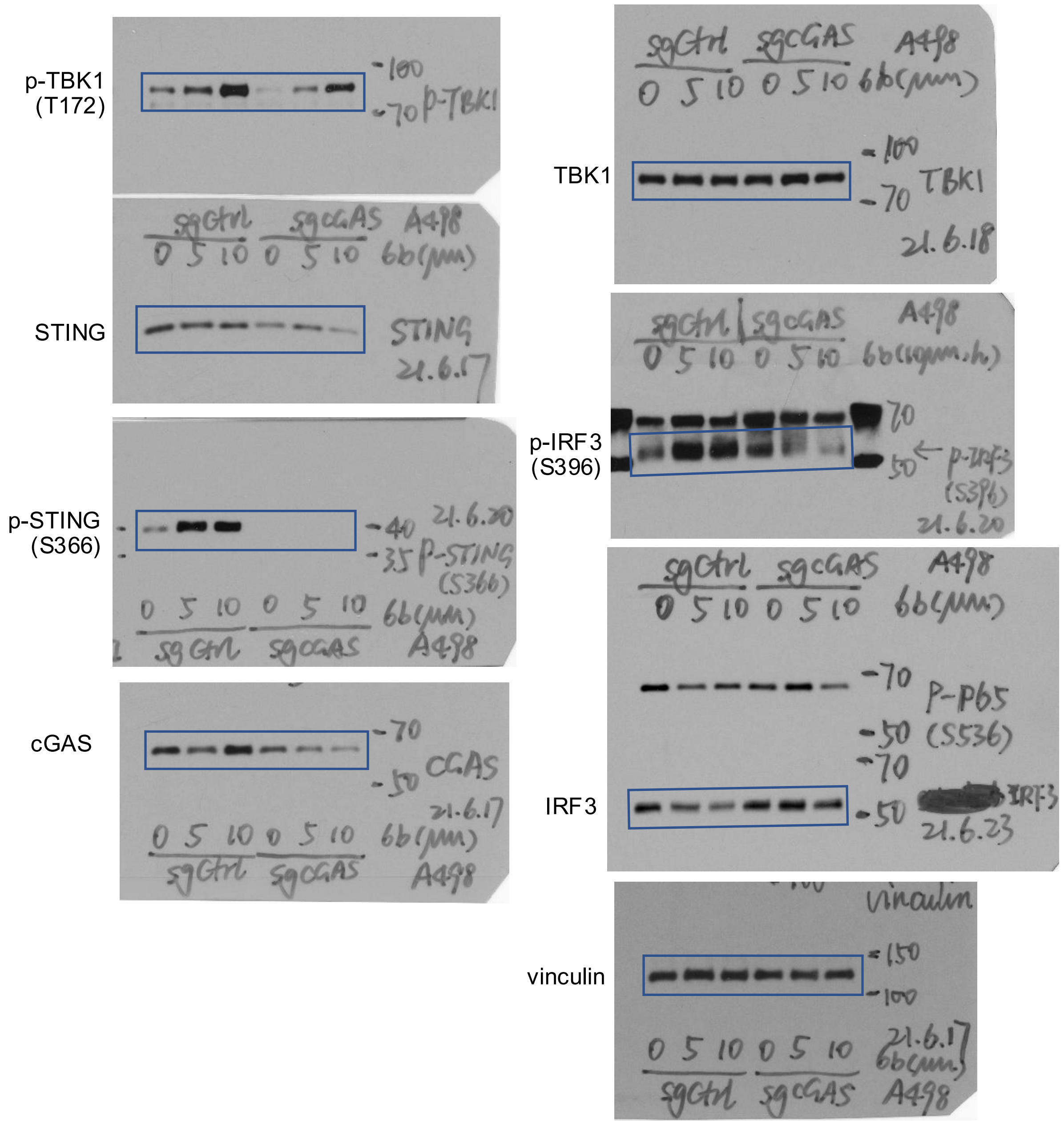

Full unedited blot for Figure S4R

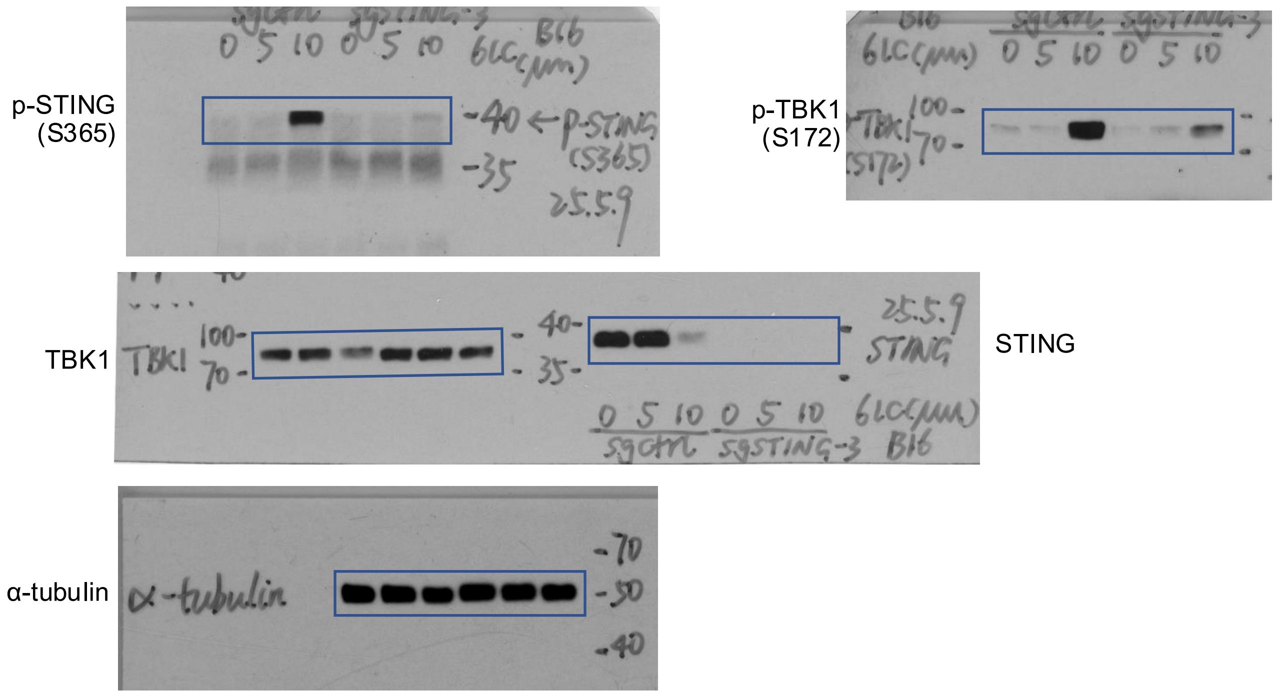

Figure S5

Full unedited blot for Figure S5A

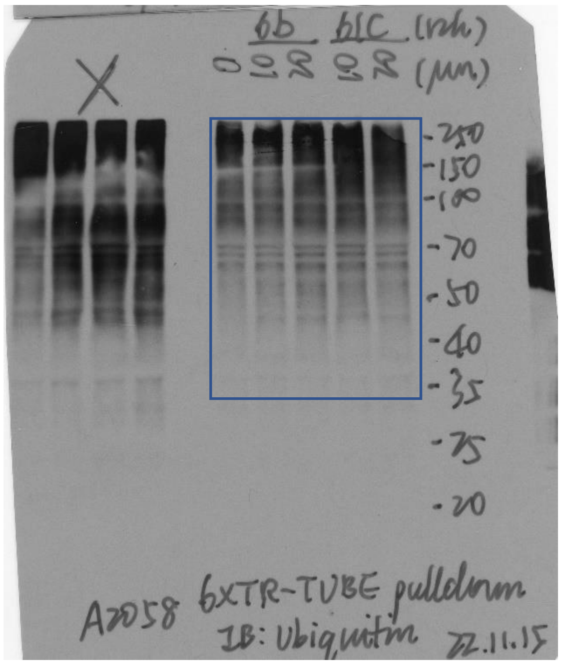

ubiquitin

Full unedited blot for Figure S5C

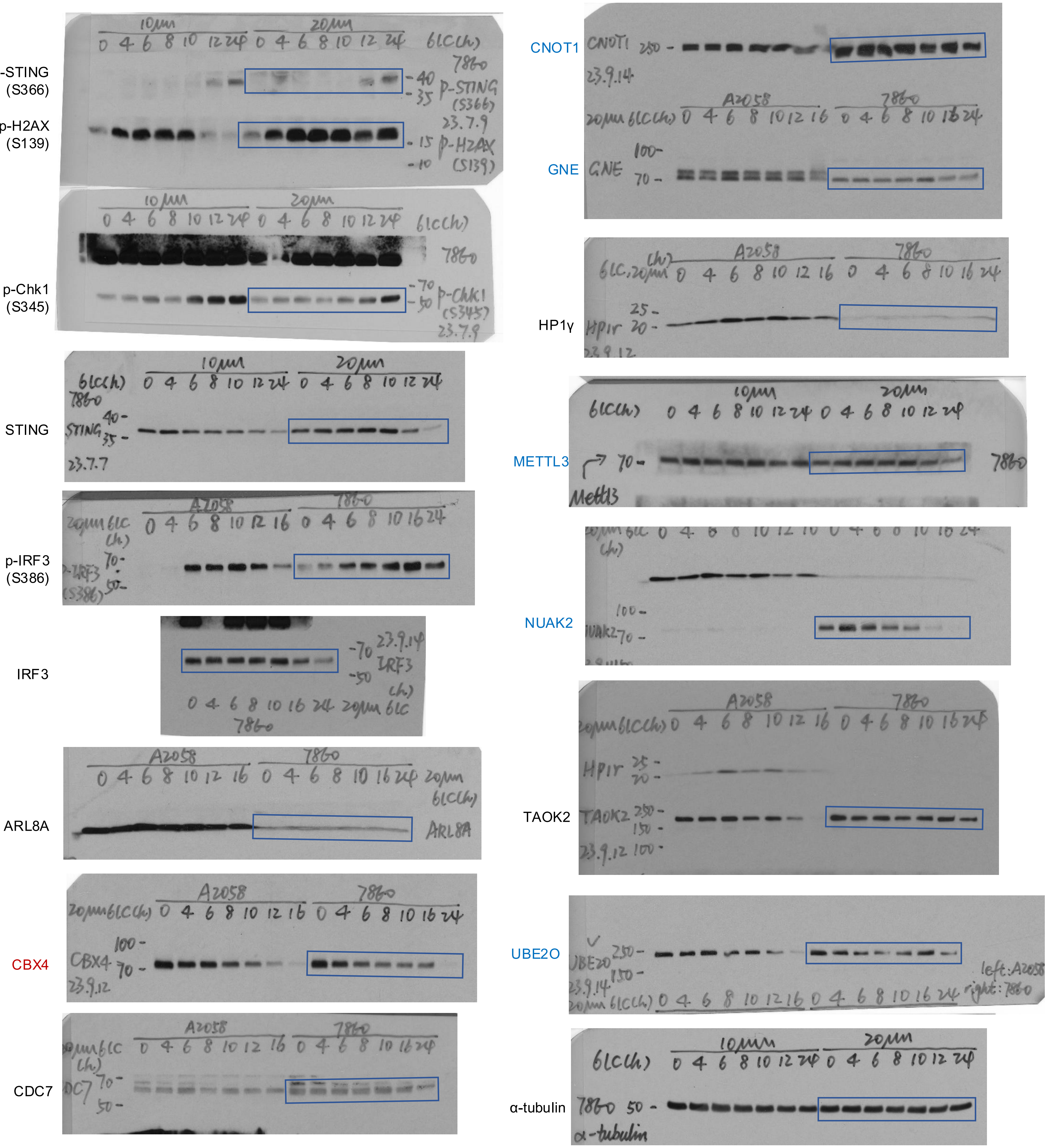

Full unedited blot for Figure S5D

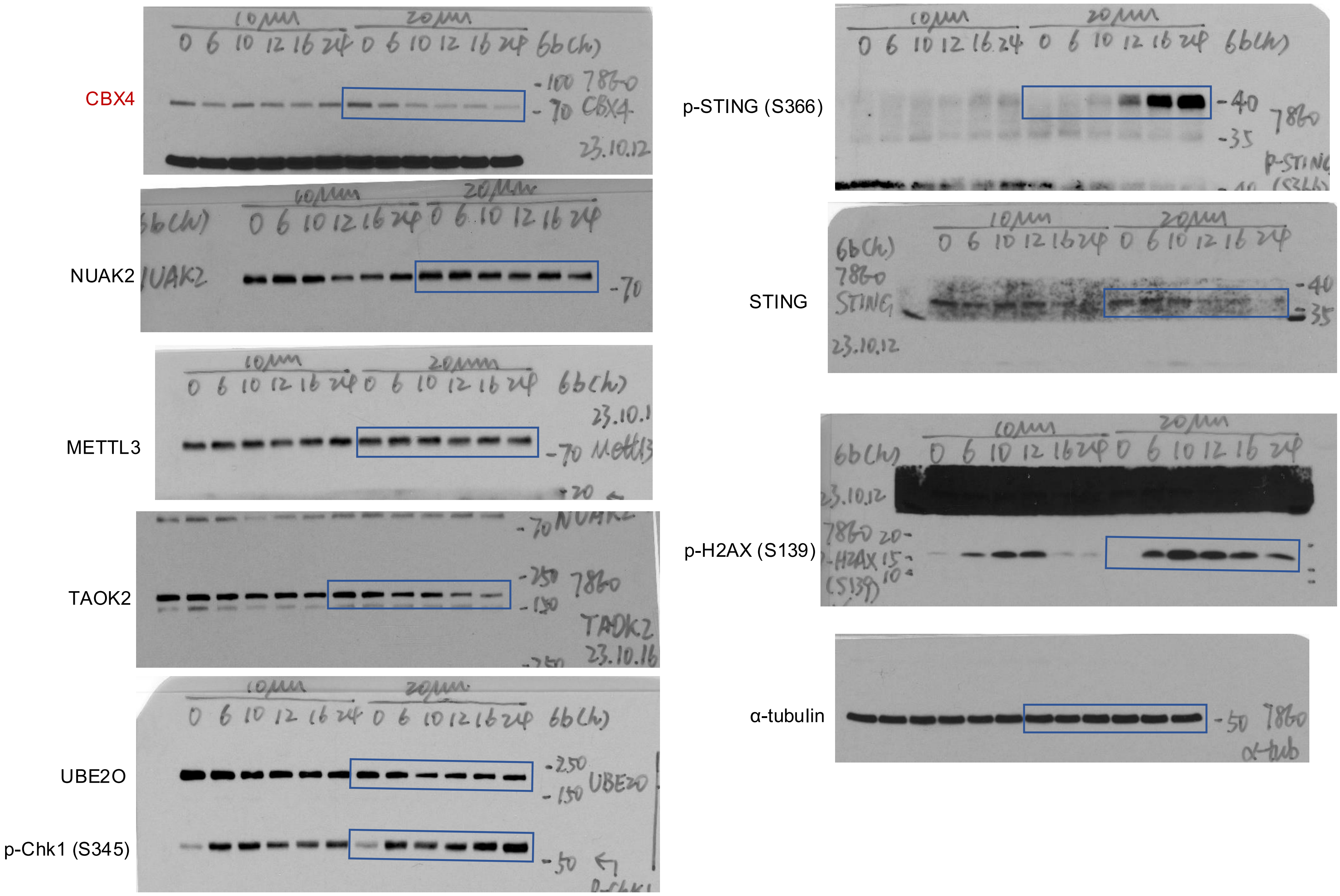

Full unedited blot for Figure S5E

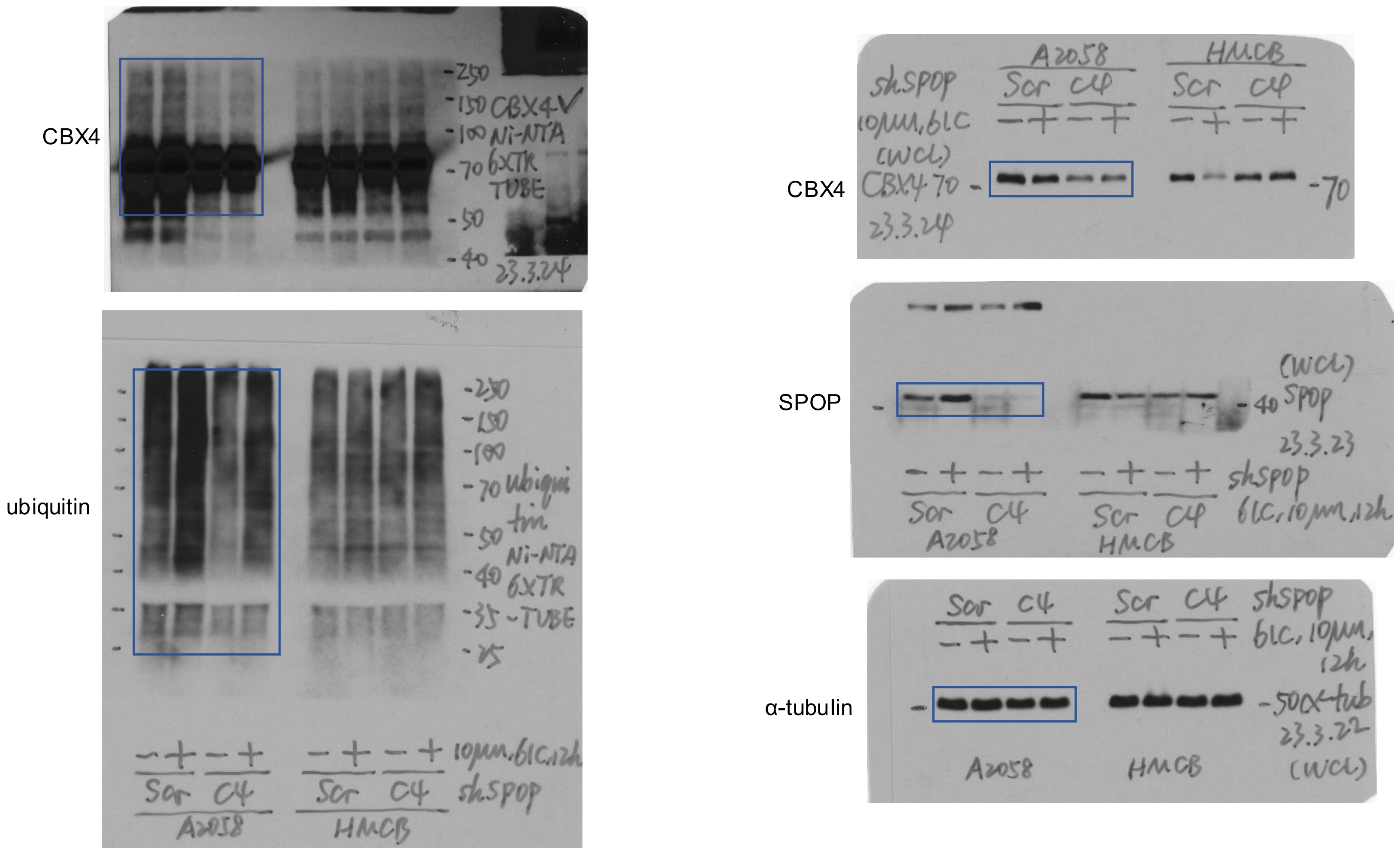

Full unedited blot for Figure S5F

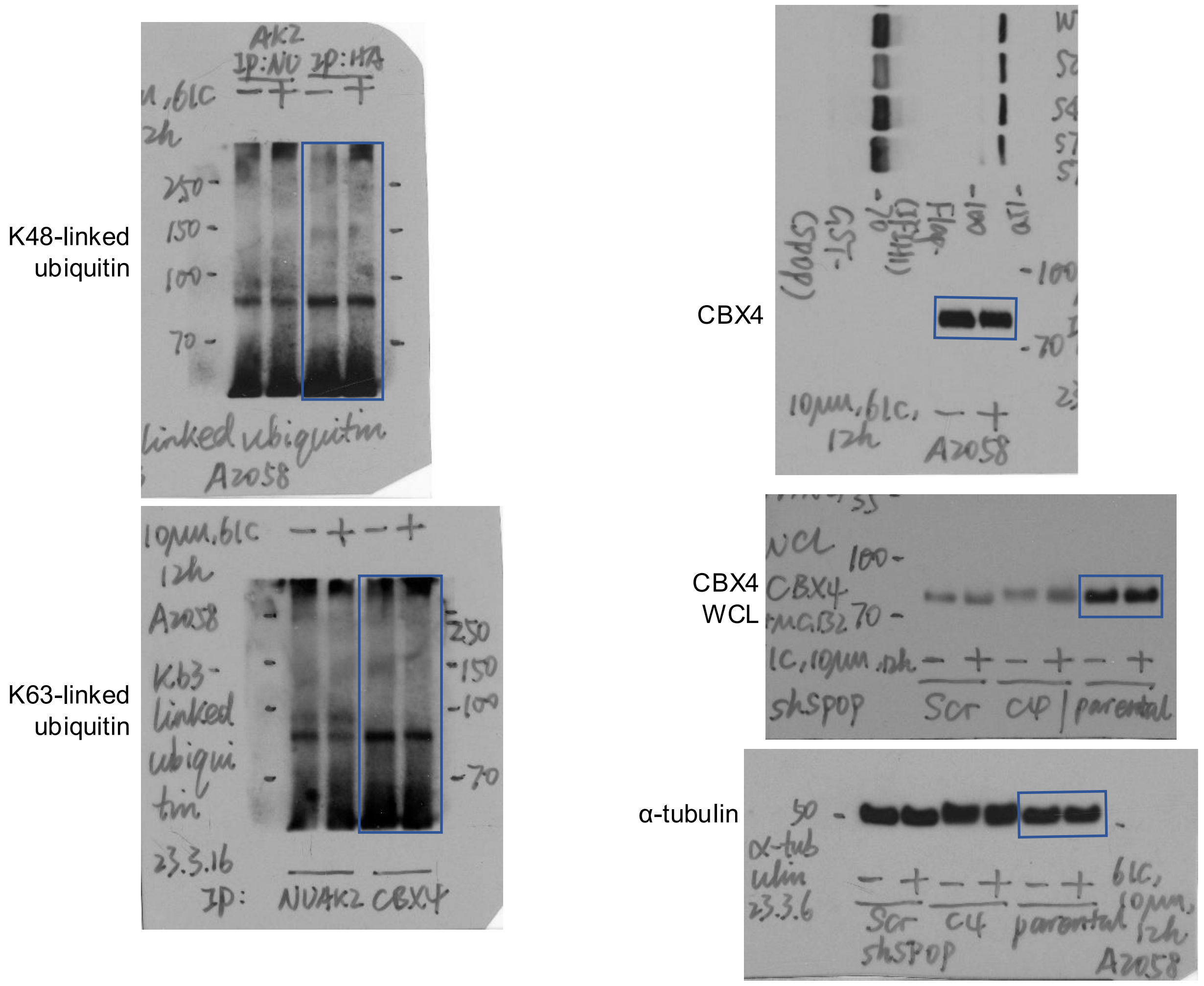

Full unedited blot for Figure S5I

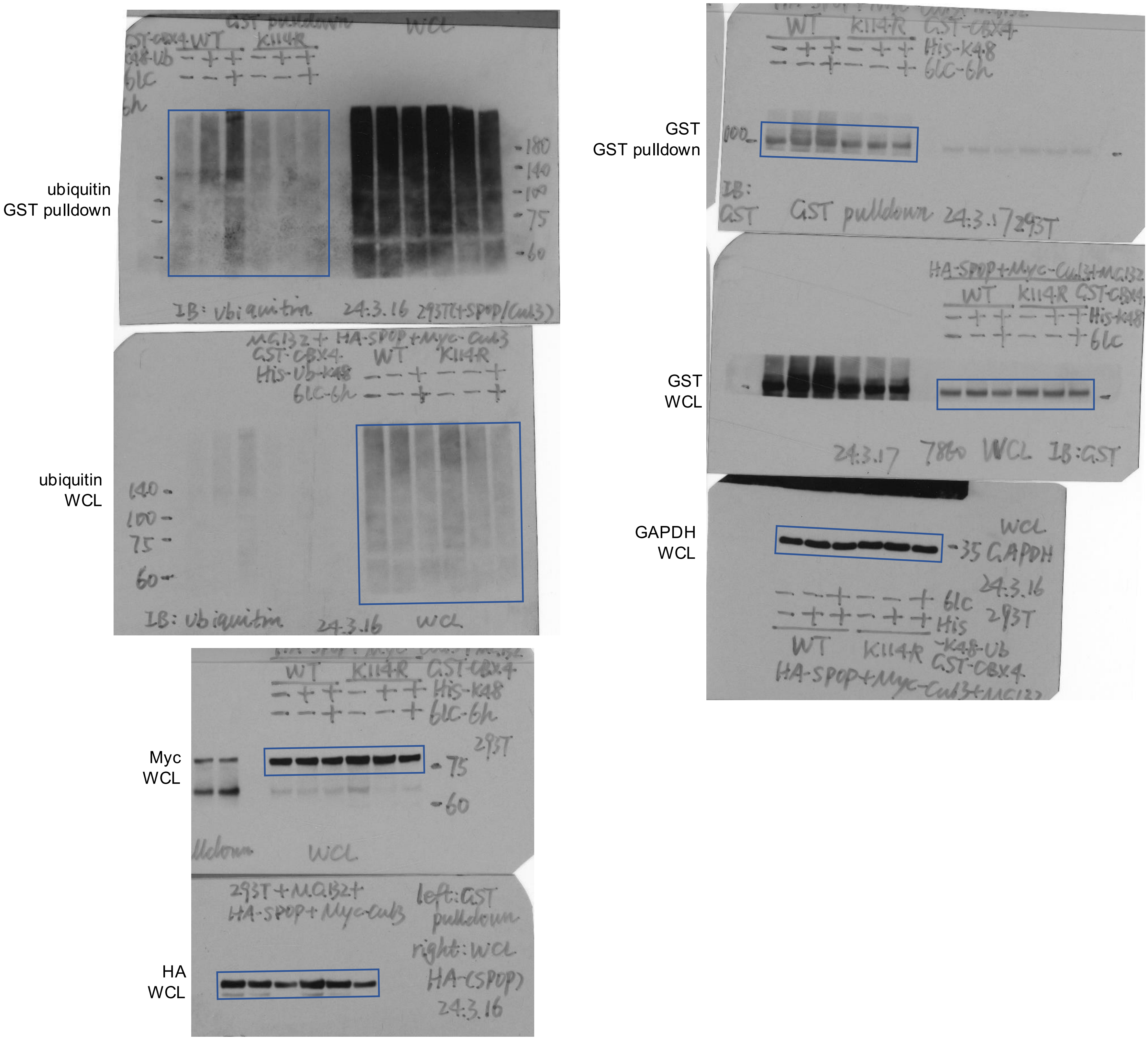

Full unedited blot for Figure S5J

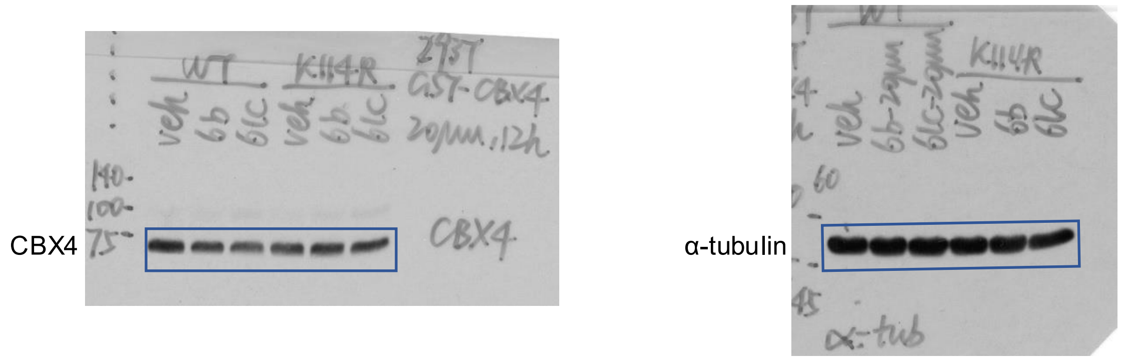

Supplement: Unedited blot and gel images [file jci-135-191772-s248.pdf]
